# Nitric oxide triggers a transient metabolic reprogramming in Arabidopsis

**DOI:** 10.1038/srep37945

**Published:** 2016-11-25

**Authors:** José León, Álvaro Costa, Mari-Cruz Castillo

**Affiliations:** 1 Instituto de Biología Molecular y Celular de Plantas (Consejo Superior de Investigaciones Científicas–Universidad Politécnica de Valencia), CPI Edificio 8E, Avda. Ingeniero Fausto Elio s/n, 46022 Valencia, Spain

## Abstract

Nitric oxide (NO) regulates plant growth and development as well as responses to stress that enhanced its endogenous production. Arabidopsis plants exposed to a pulse of exogenous NO gas were used for untargeted global metabolomic analyses thus allowing the identification of metabolic processes affected by NO. At early time points after treatment, NO scavenged superoxide anion and induced the nitration and the S-nitrosylation of proteins. These events preceded an extensive though transient metabolic reprogramming at 6 h after NO treatment, which included enhanced levels of polyamines, lipid catabolism and accumulation of phospholipids, chlorophyll breakdown, protein and nucleic acid turnover and increased content of sugars. Accordingly, lipid-related structures such as root cell membranes and leaf cuticle altered their permeability upon NO treatment. Besides, NO-treated plants displayed degradation of starch granules, which is consistent with the increased sugar content observed in the metabolomic survey. The metabolic profile was restored to baseline levels at 24 h post-treatment, thus pointing up the plasticity of plant metabolism in response to nitroxidative stress conditions.

Nitric oxide (NO) is endogenously produced in diverse living organisms and regulates a wide array of biological processes. In plants, NO regulates developmental transitions such as seed germination^1^, photomorphogenesis^2^,^3^, flowering^4^,^5^), fruit ripening^6^ and leaf senescence^7^,^8^. NO is also a key regulatory molecule in the response of plants to environmental stress^9^,^10^. As a free radical, NO is prone to react with free radical oxygen species and also with metals^11^. It has been reported that NO can enhance or reduce the redox status of the plants depending on either acting in a chronic or acute mode^12^. NO has the potential for altering the function, activity, stability and subcellular localization of many target proteins through post-translational modifications (PTMs). NO induces PTMs such as S-nitrosylation of C and nitration of Y^13^ but also ubiquitylation of K and phosphorylation of S, T and Y^14^. PTMs alter the activity or the function and also the stability of target proteins through regulation of proteolytic degradation or subcellular re-localization^15^.

The major sources of NO in the atmosphere are those derived from industrial activity and car engines^16^ as well as the microbial-related release from soils^17^. Because of the spontaneous conversion of NO to NO_2_ under aerobic conditions, it is frequent to talk about NOx when NO is supplied in an oxygenated environment. Despite NO being released by anthropogenic or by biotic soil microorganism activities, the levels of NO have been increasing continuously in the Earth atmosphere since industrial revolution started^18^. In this context, plants may be exposed to relatively high NO concentrations that would potentially alter their growth and responses to the environment. On the other hand, NO is also endogenously produced in plants through both oxidative and reductive biosynthetic pathways, and its production is enhanced under stress conditions^19^,^20^. The oxidative status generated by reactive oxygen species in stressed plants seems to be alleviated by NO through the improvement of the antioxidant capacity, thus contributing to redox homeostasis^21^.

In contrast to the increasing knowledge of the effect of NO on protein function, our current knowledge on the NO impact on global metabolome of plants is rather scanty. Although targeted metabolic approaches have been reported in addressing the effect of oxidative stress on redox-related metabolites^22^, a global metabolomic approach of NO-triggered effects in plants is still pending. We employed such untargeted metabolomic approaches to identify the metabolic targets of NO in plants exposed to an acute dose, which resembles a plant response to an extreme environmental stress condition. Metabolomic data analyses allowed us to identify polyamines, phospholipids amino acid and sugars as main metabolites involved in the activation of plant responses to nitroxidative stress.

## Results

### Metabolomic analyses reflects a transient reprogramming response to exogenous NO

**Figure 1:**
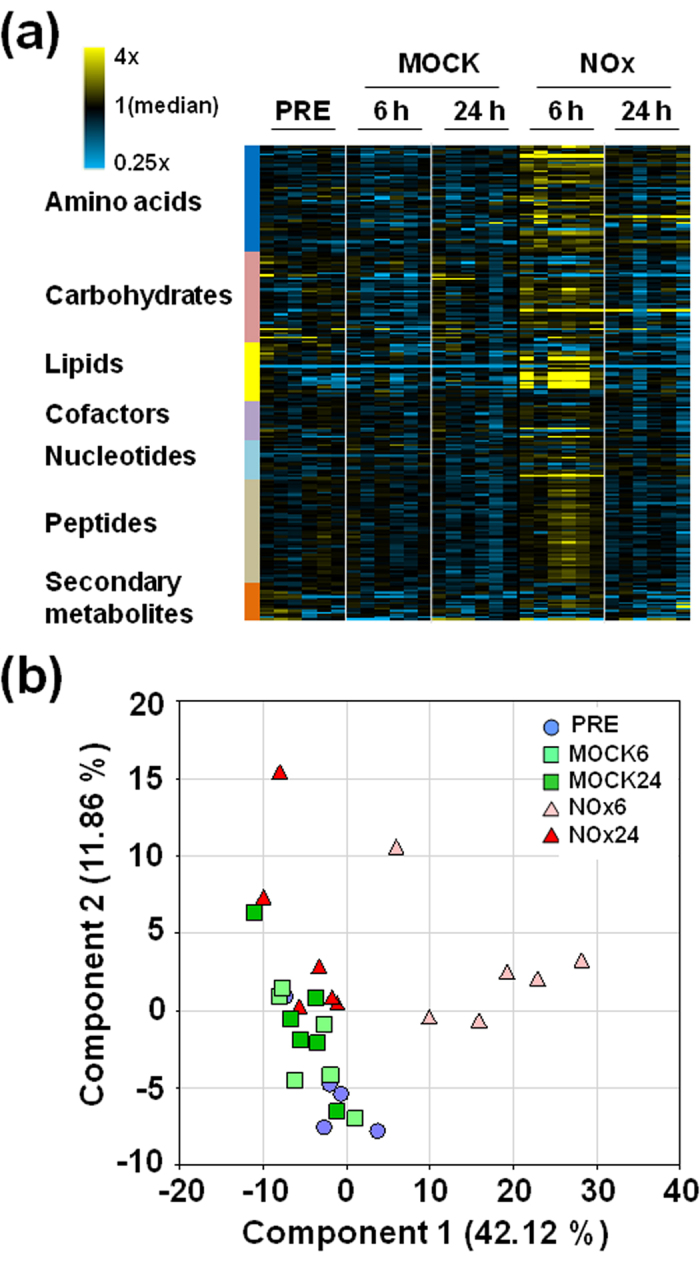
Metabolomic Changes after Applying an NO Pulse to Arabidopsis Plants. (**a**) Heat map showing the increased (yellow) or decreased (blue) concentration of metabolites at the indicated times after treatment of plants (NO) or untreated (MOCK) control. (**b**) Principle Component Analysis (PCA) plot. Blue circles represent the samples before treatment, green squares the mock-treated samples and red triangles to NO treated samples at the indicated times after pulse.

*Arabidopsis thaliana* seedling exposed to a pulse of NO gas were used as model for mimicking an acute exposure of plants to a peak in environmental levels of NO or to intracellular accumulation of large amounts of NO in the production foci in plants under stressful conditions. Seedlings were exposed to a single dose of 300 ppm NO for 5 min and then, samples were harvested for untargeted metabolomic analyses comprising a total of 232 named biochemicals. Similar analyses were also performed in mock treated plants. Samples were collected at the time of the treatment (PRE), and then at 6 h and 24 h after exposure to NO or mock treatment (Fig. 1a). Plants exposed to NO underwent a transient though remarkable metabolic alteration by 6 h after treatment. A statistically significant increase in the amount of lipid, carbohydrate, amino acid and nucleotide categories of analyzed metabolites was detected in plants by 6 h after exposure to NO (Fig. 1a). By 24 h, the metabolomic status of treated plants was restored to that of untreated plants (Fig. 1a). Supplementary Table S1 includes the complete dataset with pathway heat maps and box and line plots for every analyzed metabolite showing the comparative fold- levels for every metabolite in the different samples as well as the statistical significance for the different comparisons. Only a few metabolites showed a significant difference when PRE and mock 6 h samples were compared, thus indicating only a small proportion of the metabolites analyzed were potentially regulated by circadian-related events in the experiment (Fig. 1a and Supplementary Table S1). By contrast, around 70% of the metabolites showed significant alteration in their endogenous levels by 6 h after exposure to NO (Fig. 1a and Supplementary Table S1). Significantly lower number of changes were detected in samples collected at 24 h after exposure (Fig. 1a and Supplementary Table S1), thus suggesting NO treatment has an extensive though transient metabolic effect on Arabidopsis plants. The Principal Component Analysis (PCA) confirms this general pattern. The PRE and MOCK 6 h or 24 h samples did not separate well, the NOx 24 h samples had only partial separation, and indeed the NOx 6 h samples were all clearly separated from this grouping (Fig. 1b). Moreover, to assess the compounds which meet the statistical criteria for significant differentiation between the analyzed samples, we applied two types of tests to compare the five experimental groups. The first approach was to compare each treated or mock-treated group to the PRE samples, using Welch’s Two Sample t-Test. This approach yields information on the effect of time parameter on the experiment. A summary of the numbers of biochemicals that achieved statistical significance (p ≤ 0.05), as well as those approaching significance (0.05 < p < 0.1), is shown in Table 1. One would expect approximately 10–15 compounds to reach significance by chance alone with 232 compounds tested. In these comparisons the mock samples showed only 12 or 24 compounds at 6 h and 24 h, respectively, which were significant (Table 1), thus suggesting the mock treatment had little effect on the metabolome. By contrast, the NOx treatment resulted in 133 compounds being significant at the 6 h time point, and 55 at 24 h (Table 1), consistent with a robust experimental effect. To eliminate any diurnal effects in the data, we used an ANOVA Contrast test to compare the NOx to MOCK plants at each time point individually, and to score the time-related differences within each treatment pair. Again, the results were quite consistent with the observations taken from the heat map (Fig. 1a), PCA (Fig. 1b), and t-test results (Table 1). There were 154 compounds meeting the cut-off when comparing NOx 6 h to MOCK 6 h, but only 29 when compared at the 24 h time point (Table 1 and Supplementary Table S1), consistent with the idea that plants experienced a transient and acute response to NO exposure, and then, most metabolites returned to baseline levels after 24 h.

### Altered levels of amino acids and dipeptides suggests NO treatment increased protein breakdown

The elevated content of proteinogenic amino acids and dipeptides in the NOx 6 h compared to the MOCK 6 h or PRE samples (Supplementary Table S1) strongly suggest NO is inducing protein degradation. The exceptions to this pattern were amino acids that serve important metabolic functions in addition to protein synthesis. This includes glutamate, glutamine, and arginine, which are involved in nitrogen trafficking and storage, and glycine and serine, which are utilized in the photorespiration reactions, which endogenous content did not change significantly by NO treatment (Supplementary Table S1). The branched chain amino acids, leucine, isoleucine, and valine are typically at relatively low levels in cells, and function almost exclusively in protein synthesis, so an increase in their levels often indicates net protein turnover. These compounds, as well as the aromatic amino acids were more than twice the levels in NOx 6 h samples relative to MOCK 6 h (Supplementary Table S1). The aromatic amino acids are involved in other important pathways, such as the large phenylpropanoid family pathways (phenylalanine), tocopherol production (tyrosine), and hormone and glucosinolate synthesis (tryptophan), which were relatively unchanged or even dropped by NOx treatment (Supplementary Table S1). Moreover, the content of a common precursor of aromatic amino acid biosynthesis such as shikimate was lower in NO-treated relative to mock-treated plants at both 6 and 24 h time points (Supplementary Table S1), thus suggesting either the increased levels of aromatic amino acids caused a feed-back inhibition on the biosynthetic pathway or, alternatively, their increased levels are the result of active protein turnover.

**Figure 2:**
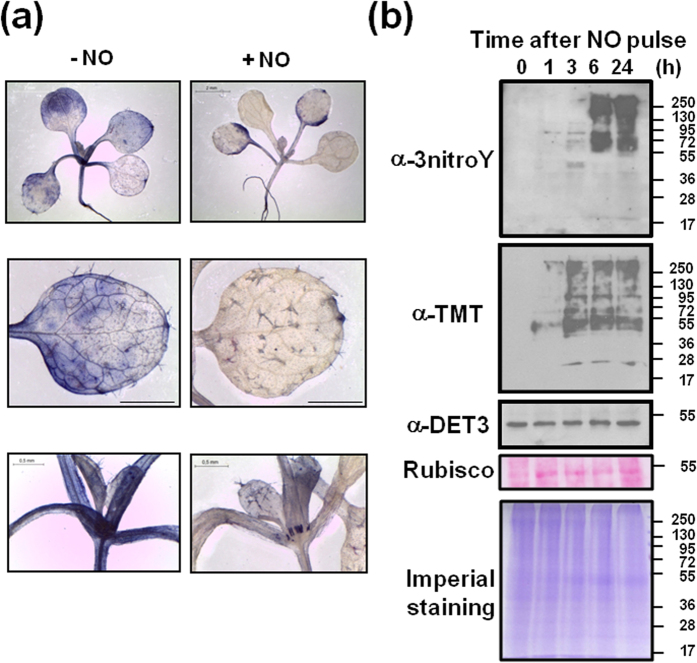
Superoxide content reduction and post-translational modifications induced by NO. (**a**) Control untreated seedlings (−NO) and NO-exposed seedlings (+NO) were stained with Nitroblue tetrazolium from 30 min to 20 h after treatment. (**b**) Nitration and S-nitrosyltion of proteins in NO-treated plants. Each lane was loaded with 10 μg of proteins. The marks and numbers at the right side of panels indicate the position of molecular weight markers in kDa. The general pattern of protein is shown by staining of a replicate gel with Imperial (Thermo Scientific). Blot replicates were probed with the antibodies indicated at the left side of panels. α3-nitroY, anti-3-nitrated-Y; αTMT, anti-Tandem Mass Tagged-S-nitrosylated proteins. Equal loading was checked by Coomassie-stained large subunit of Ribulose-1,5-Bisphosphate Carboxylase/Oxygenase (Rubisco) and by using αDET3, anti-De-Etiolated 3. Data shown are representative of three independent experiments with similar results.

We have observed that by 30 min after exposure to NO the nitroblue tetrazolium staining of superoxide anion in Arabidopsis seedlings was significantly reduced, thus suggesting NO efficiently scavenged superoxide (Fig. 2a). Peroxynitrite, a potent nitrating agent, is formed upon superoxide scavenging by NO, thus suggesting nitration of target proteins may occur in plants exposed to NO. We have analyzed the total and NO-related modified protein profile in plants after the NO pulse. No general changes were observed in the total protein pattern detected after monodimensional electrophoresis (Fig. 2b). Exogenous NO enables the modification of proteins through S-nitrosylation or nitration of cysteine and tyrosine residues, respectively^13^. We found that many nitrated and S-nitrosylated proteins started to accumulate by 1 h and reached maximum accumulation between 3 and 6 h after exposure of plants to NO (Fig. 2b).

### Acute exposure to NO triggers the accumulation of polyamines and responses to oxidative stress

**Figure 3:**
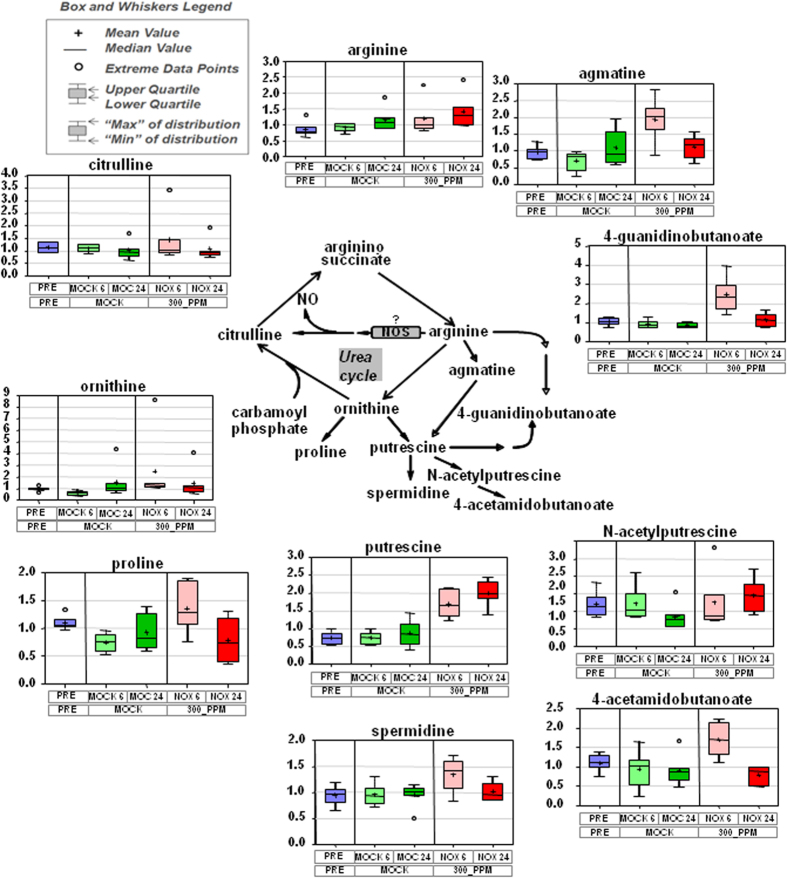
Enhanced polyamine content in plants exposed to NO. Scheme showing the biosynthesis of polyamines derived from the urea cycle and the box plots for every metabolite before treatment (blue) and at indicated times after mock- (green) or NO-treatment (red). Light and dark tones correspond to 6 h and 24 h, respectively, after treatment.

Plants exposed to NO also showed specific effects on α-ketoglutarate-derived amino acids of the glutamate family. The levels of γ-aminobutyrate (GABA) and 2-aminobutyrate were slightly increased, and the increase was higher (4.3-fold) for γ-hydroxybutyrate (GBH) by 6 h after exposure to NO (Supplementary Table S1). It has been reported that the metabolism of GABA leads to accumulation of GBH when plants are exposed to low oxygen or high light conditions, and also that under those stress conditions accumulation of GABA and alanine was also detected^23^, which is in agreement with our data (Supplementary Table S1). Also in the glutamate family of amino acids, the levels of proline and ornithine were 1.8- and 4-fold increased, respectively, and also their corresponding N-acetyl derivatives were 1, 7- and 5.8-fold augmented, respectively (Supplementary Table S1). The increased levels of proline and ornithine were also accompanied by significant increases in the content of polyamines such as putrescine (2.3-fold) and spermidine (1.4-fold) as well as the precursor agmatine (2.8-fold) in NO-exposed plants after 6 h (Fig. 3 and Supplementary Table S1).

Cysteine is an unique example where active synthesis of an amino acid was evident in response to NO treatment. It is formed from serine in two steps, through the O-acetylation of serine by acetyl-CoA to form O-acetylserine, followed by the incorporation of hydrogen sulfide, displacing the acetyl group^24^. Cysteine synthesis is often the rate limiting step for the formation of glutathione, a critical compound in cellular redox responses^25^. We found an increase above 2-fold in all these compounds in NOx 6 h plants. O-acetylserine and the non-enzymatically rearranged N-acetylserine were 17- and 11-fold increased, respectively (Supplementary Table S1), thus suggesting the exposure to NO triggered a strong demand for cysteine production as a mean to remediate oxidative stress. In agreement with that, perturbations of several other oxidative markers, namely increased of oxylipins 13-HODE, 9-HODE and 9,10-hydroxyoctadec-12(Z)-enoic acid produced through the LOX pathway^26^, were detected. Also consistent with NO-treated plants undergoing oxidative stress, the endogenous content of threonate, which is a downstream product of ascorbate metabolism^27^, were more than 4-fold higher in NO-treated plants by 6 h, and also a moderate increase in both oxidized and reduced glutathione content was detected (Supplementary Table S1). By contrast, the content of α-tocopherol, which together with glutathione and ascorbate constitute the triad of main antioxidants in plants undergoing oxidative stress^28^, were around 50% lower by 6 h after NO treatment (Supplementary Table S1). These data together suggest that NO-exposed plants respond to oxidative stress through a complex process.

### Altered lipidome reflects changes in lipidic structures such as membranes and cuticle in NO-exposed plants

**Figure 4:**
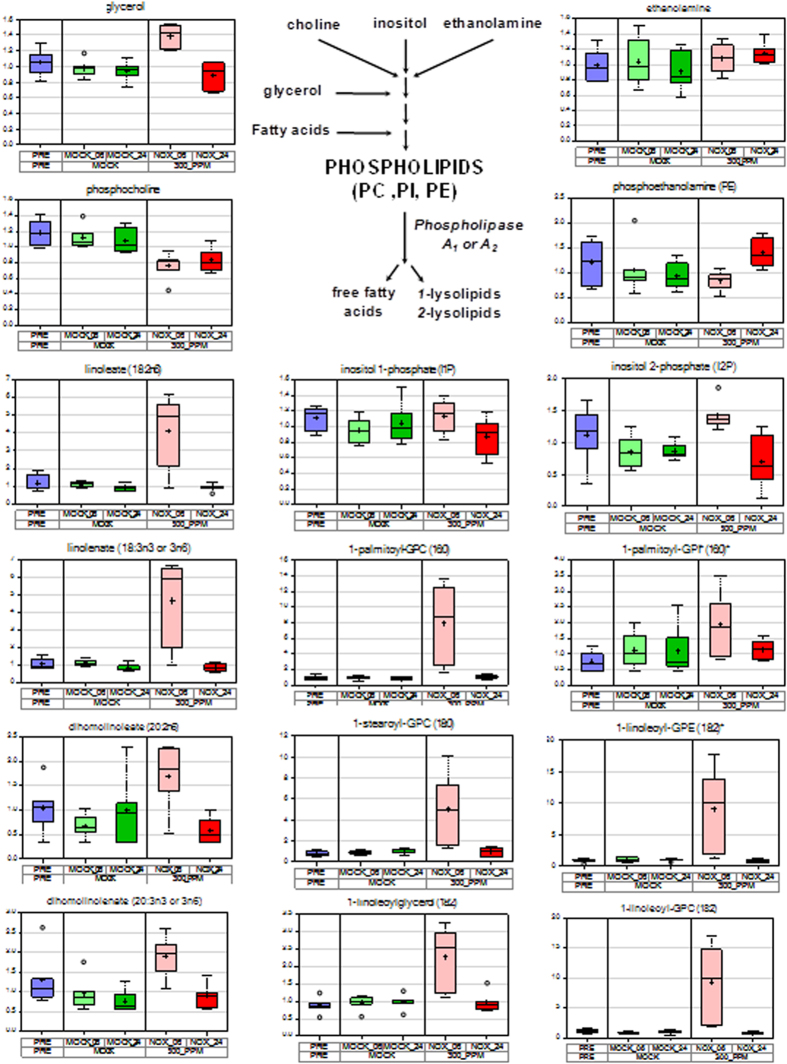
Altered phospholipid catabolism in plants by 6 h after exposure to NO. Scheme showing the metabolism of phospholipids and the box plots for fatty acids and lipids before treatment (blue) and at indicated times after mock- (green) or NO-treatment (red). Light and dark tones correspond to 6 h and 24 h after treatment, respectively.

**Figure 5:**
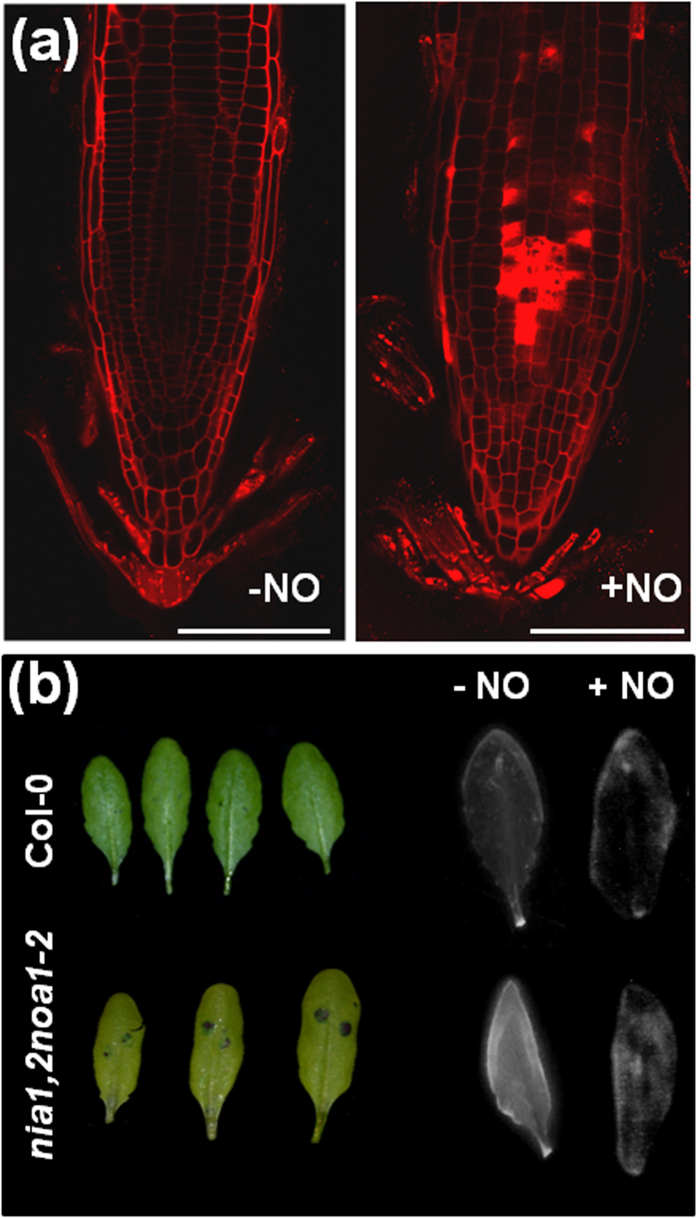
Alterations in the permeability of lipidic structures. (**a**) Permeability of plasma membrane in roots of NO-treated plants. Untreated (−NO) or NO-treated (+NO) roots from wild type Col-0 plants were stained with propidium iodide at 30 min after exposure to NO. Images were obtained with a confocal microscope Leica TCS SL with excitation at 488 nm and emission at 598–650 nm range. Scale bars: 50 μm. (**b**) Effect of NO on leaf cuticle permeability. In the left panel, two drops of 10 μl of toluidine blue solution were applied on the upper side of undetached leaves from wild type Col-0 and NO-deficient *nia1,2noa1-2* mutant plants. After 2 h, leaves were extensively washed with water, excised and photographed to show penetration or not of toluidine blue through cuticle. In the right panel, leaves from untreated (−NO) or NO-treated (+NO) plants were detached at 6 h after NO pulse, bleached with ethanol, stained for 1 min with calcolfluor white and extensively rinsed with water before photographed under UV light. Data shown are representative of five and three independent toluidine blue and calcofluor white experiments, respectively, with similar results.

The content of compounds belonging to the lipid category underwent large changes in NO-treated plants. Alterations were strictly limited to the six hour time point (Fig. 4). A significant increase in the free polyunsaturated fatty acids (PUFAs) content of around 4-fold for linoleate and linolenate, and around 2-fold increase for the C20 PUFAs dihomolinoleate and dihomolinolenate was detected (Supplementary Table S1 and Fig. 4). We also found a strong increase in several lyso-lipids such as 1-palmitoyl-GPC (8.9-fold), 1-palmitoyl-GPE (13.2-fold), 1-stearoyl-GPC (5.7-fold), 1-linoleoyl-GPC (10.8-fold), 1-linoleoyl-GPE (8.9-fold), 2-linoleoyl-GPC (4.8-fold) and 2-linoleoyl-GPE (5.4-fold) (Supplementary Table S1 and Fig. 4). Altered content of free fatty acids and phospholipids are likely connected to processes of lipid trafficking in either membrane remodeling or alteration of lipidic leaf structures such as cuticles^29^. We tested whether cell membranes or cuticles were altered in Arabidopsis plants upon exposure to NO. Nitrooxidative stress produced by the acute pulse of NO can severely damage membranes by lipid peroxidation^30^, thus altering their permeability. The integrity of membranes was monitored *in vivo* by staining roots with propidium iodide that only stains nuclei if membranes become permeable, but it remains staining membranes and walls if cell membranes are intact. Roots of untreated plants display non altered plasma membranes as demonstrated by propidium iodide decorating the cell membrane with no permeation into the cells (Fig. 5a). By contrast, by 30 min after exposure to NO the roots of NO-treated plants displayed numerous cells with propidium iodide-stained nuclei, thus indicating the permeability of their membranes was altered (Fig. 5a). Also the formation, integrity and permeability of the leaf cuticle are highly connected to lipid metabolism in Arabidopsis^31^. We checked whether the permeability of the leaf cuticle was influenced by the exposure to NO. Wild type Arabidopsis plants treated with exogenous NO as well as the Arabidopsis *nia1nia2noa1-2* triple mutant, depleted of endogenous NO^32^, were tested for cuticle permeability. Assays of cuticle permeability after application of toluidine blue or calcofluor white demonstrated an enhanced permeability of the cuticle in NO-deficient leaves that was reversed by treatment with exogenous NO (Fig. 5b).

### Altered purine, pyrimidine and chlorophyll metabolism suggest NO enhanced nucleic acid turnover, chlorophyll degradation and non-programmed cell death

The metabolomic data also reflected a significant increase in metabolites involved in purine and pyrimidine metabolism. The levels of allantoin, guanine, urate, cytidine, cytosine-2′,3′-cyclic monophosphate, pseudouridine, uridine and uracil were all increased (between 1.5- and 7.6-fold) by 6 h after NO treatment (Supplementary Table S1). However, all these metabolites returned to the baseline levels by 24 h, with special emphasis on uracil, which moved from a 7.6-fold increase by 6 h to non-significantly changed levels by 24 h (Supplementary Table S1). It is noteworthy mentioning that, in turn, the levels of nucleosides such as adenosine, adenosine-2′-monophosphate and inosine are between 20 and 45% reduced by 6 h after plants were exposed to NO (Supplementary Table S1). In agreement with the enhanced metabolism of purines and pyrimidines, we found a pattern of progressive genomic DNA degradation upon exposure of plants to the NO pulse (Supplementary Fig. S1a).

**Figure 6:**
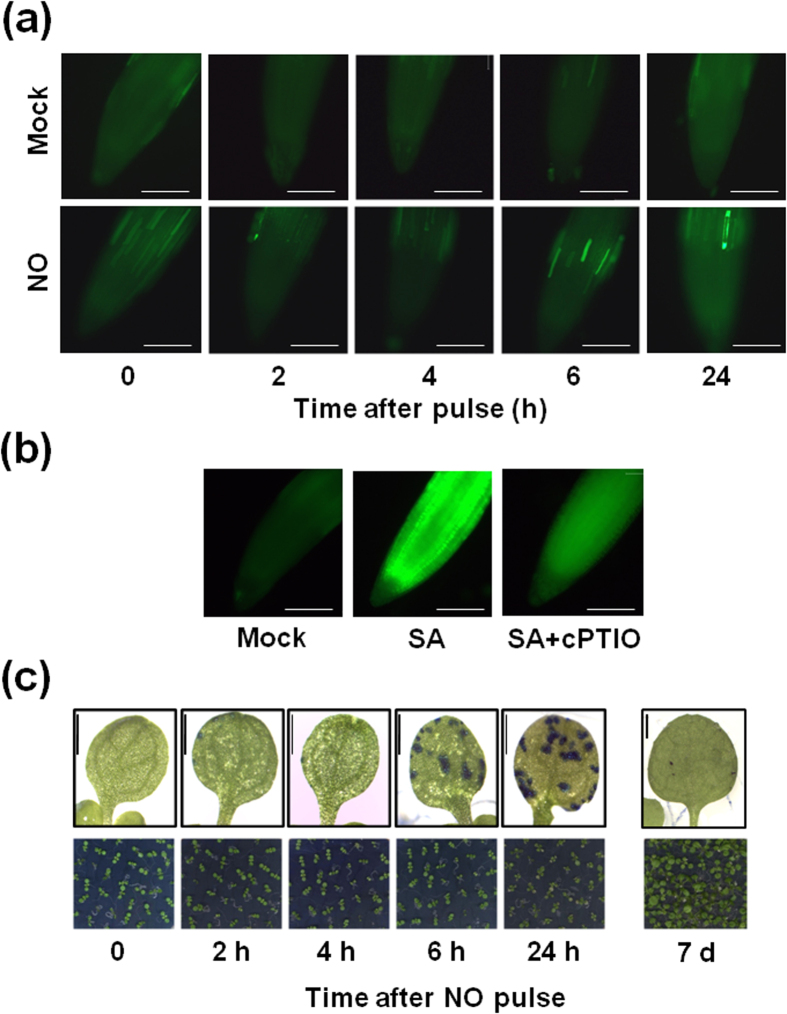
Endogenous NO content and cell death upon exposure to exogenous NO. Seedlings (8 day-old) were treated with (**a**) exogenous NO or treated with air as control (Mock) and (**b**) mock treated or treated with 0.25 mM salicylic acid (SA) or with SA plus 1 mM 2-(4-Carboxyphenyl)-4,4,5,5-tetramethylimidazoline-1-oxyl-3-oxide (SA + cPTIO) for 1 h, and at the indicated times after exposure, the endogenous NO content was assayed by root staining with DAF-FM DA. Scale bars: 100 μm. (**c**) Cell death was assayed by Evans blue staining of plants at the indicated times after exposure to NO. Scale bars: 1 mm. The appearance of the plants for every time point is shown in the bottom panels. The experiments were repeated three times with similar results and representative images are shown.

On the other hand, the metabolomic data also suggest NO induces chlorophyll degradation. Pheophorbide a, which has been identified as a genuine intermediate of chlorophyll breakdown^33^, was 3.7-fold increased by 6 h after NO treatment (Supplementary Fig. S1b), thus suggesting Pheophorbide a oxidase (PAO) activity should be reduced in NO-exposed plants. The absence of PAO activity in different plants has been correlated with increased cell death, and pheophorbide a has been reported to be involved in both light-dependent and light-independent cell death mechanisms^34^,^35^. We checked whether treatment with exogenous NO alters the endogenous levels and leads to cell death in exposed leaves. Figure 6a shows the endogenous levels of NO after staining roots with DAF-FM diacetate. The endogenous levels slightly decreased by 2 and 4 h after exposure to exogenous NO and modest increases were observed by 6 and 24 h (Fig. 6a) but increases were far below those detected in plants treated with a known inducer of NO such as salicylic acid^36^ (Fig. 6b). Evans blue staining of plants at different times after NO treatment showed blue stained spots of dead cells at 6 h and 24 h after exposure to NO (Fig. 6c) and although seedlings were slightly chlorotic by 24 h plants they were fully viable showing normal growth by seven days after treatment (Fig. 6c).

### Exposure to NO affected photorespiration and central carbon metabolism

**Figure 7:**
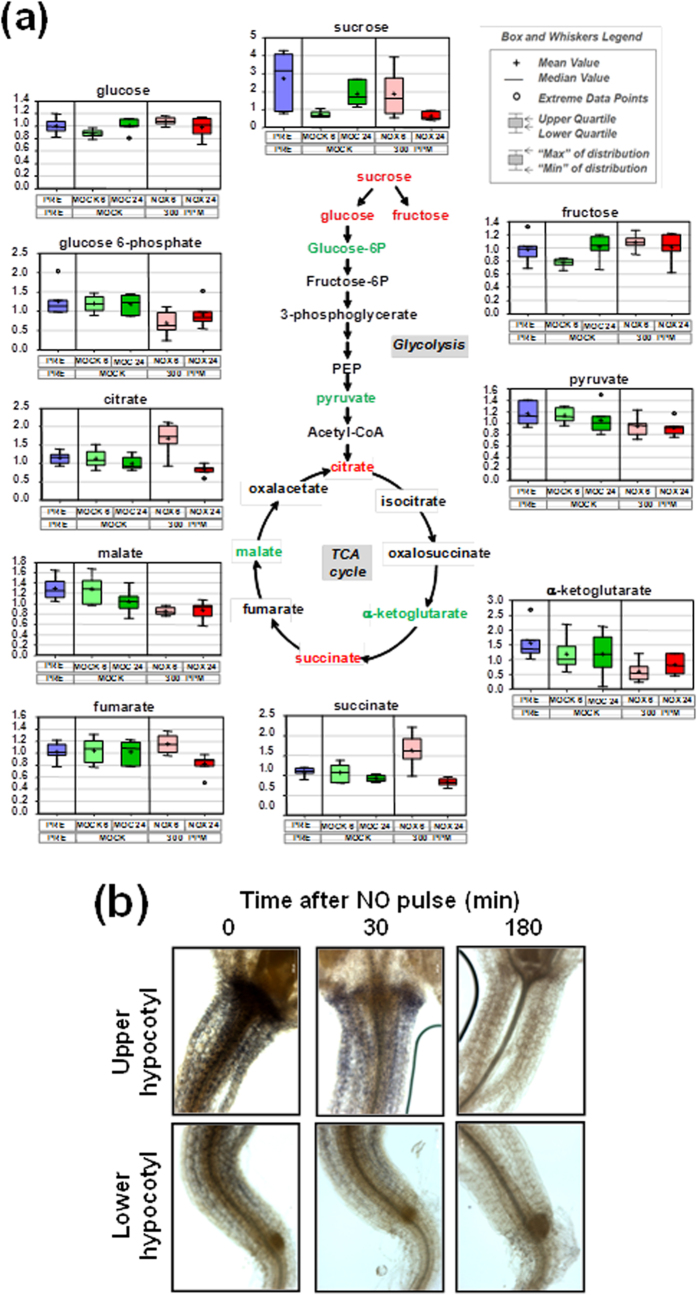
Metabolites of glycolysis and TCA cycle in plants exposed to NO. (**a**) Scheme showing the intermediate metabolites (in red increased content; in green reduced content) of the glycolysis and the TCA cycle and the box plots for every metabolite before treatment (blue) and at indicated times after mock- (green) or NO-treatment (red). Light and dark tones correspond to 6 h and 24 h, respectively, after treatment. (**b**) Starch granules were stained with Lugol´s reagent in 7-day old seedlings exposed to a 300 ppm NO pulse for the indicated times. Images for hypocotyls are representative of at least six independent plants per time point.

By 6 h after exposure to NO, the content of the glycolisis intermediates glucose-6-phosphate, glycerate and pyruvate decreased (0.57-, 0.59- and 0.83-fold, respectively) if compared to mock-treated plants at the same time point (Fig. 7a and Supplementary Table S1). It is noteworthy mentioning that glycerate, which is an intermediate in photorespiration, kept levels significantly reduced by 24 h after exposure to NO (Supplementary Table S1). Also the levels of two metabolites of the TCA cycle such as α-ketoglutarate and malate were significantly reduced by 6 h in NO-treated plants (Fig. 7a and Supplementary Table S1). Finally, sedoheptulose-7-phosphate and ribose-5-phosphate, both intermediates of the Calvin Cycle were not different at the six hour time point, but were significantly lower (0.32-, 0.58-fold, respectively) at 24 hours (Supplementary Table S1). The content of sucrose, the downstream product of photosynthesis, although increased (2.5-fold) by 6 h was significantly reduced (0.33-fold) by 24 h (Fig. 7a and Supplementary Table S1). Also different sugars including amino and nucleotide sugars as well as disaccharides were increased (between 1.4- and 3-fold) in the 6 h time point in NO-treated plants (Supplementary Table S1). Remarkably, three of those compounds arabitol, ribulose and ribitol were strongly reduced (0.04-, 0.54- and 0.74-fold, respectively) by 24 h after NO treatment (Supplementary Table S1). In agreement with exogenous NO altering photosynthetic metabolism and triggering differential accumulation of sugars, we have detected a significant reduction in the starch granules in the hypocotyls of NO-treated seedlings by 3 h after exposure to NO (Fig. 7b).

## Discussion

The nature of NO as a gas and as a free radical molecule makes it highly diffusible but at the same time very reactive. NO mode of action is likely based on the existence of sharp concentration gradients with high local concentrations in the microenvironment where it is produced. In an attempt to mimic conditions of high local accumulation of NO such as those reached under natural stress-related conditions, we exposed Arabidopsis plants to a short pulse of a high dose of exogenously applied NO gas. Most of the studies quantifying NO production in plants measure NO emission, which is just a fraction of the total NO synthesized inside cells. The recent implementation of techniques using microchip electrophoresis and laser-induced fluorescence detection allowed reporting intracellular concentrations of NO around 0.6 mM for human Jurkat cells that can be increased to 1.5 mM under stress conditions induced by lipopolysaccharides^37^. Unfortunately, no such approach has been implemented for plant cells. Nevertheless, we have made a conservative estimation of the exogenous NO dose that would be required to approximate the endogenous content to a concentration peak closer to those ranges of concentration. A short pulse of 300 ppm NO gas would allow to raise the endogenous NO levels to values still clearly below 0.5 mM but high enough to mimic a response in the whole plant similar to that experienced locally in the production foci under stressful conditions. We then analyzed the metabolic effects that the acute exposure to NO produced in Arabidopsis plants. The metabolome of NO-treated plants changed extensively by 6 h after treatment but alterations were transient as by 24 h the metabolome was roughly similar to those of untreated or mock-treated plants. This sharp and transient response talks about the extraordinary plasticity of Arabidopsis metabolism. It has been reported that NO counteracts oxidative stress either by acting as a direct antioxidant or by functioning as a signaling molecule that alters gene expression. When acting as an antioxidant, it mainly scavenges reactive oxygen species (ROS), such as superoxide anions to form peroxynitrite, which has a large potential in nitrating target proteins^38^. Our data are consistent with this antioxidant activity as we detected a reduction in nitrobluetetrazolium-staining of superoxide upon NO pulse treatment (Fig. 2a) and concomitantly, treated plants accumulated nitrated proteins (Fig. 2b). Moreover, NO-treated plants showed a significant increased production of polyamines and cysteine (Supplementary Table S1), which are both good indicators of NO activating processes devoted to remediate oxidative stress. However, extensive evidence suggests that NO is somehow involved in paradoxical processes exerting sometimes opposing regulatory functions. It has been reported both antioxidant and prooxidant effects of NO^12^. These paradoxical roles exerted by NO might be due to multiple factors including the relative cellular concentration, the location where it is produced and the complex interacting microenvironment. As an example, the increased polyamine production has been reported to act as antioxidant but they also trigger the production of hydrogen peroxide as a result of their metabolism^39^. In addition, the endogenous content of two important antioxidant molecules such as ascorbate and α-tocopherol^29^ were reduced in NO-treated plants (Supplementary Table S1) thus suggesting NO was somehow lowering the antioxidant potential of the plant. Although ascorbate is predominantly metabolized through the reversible oxidiation to dehydroascorbate by the ascorbate–glutathione cycle, it can also escape the ascorbate–glutathione cycle by being broken down through irreversible reactions that lead to threonate^40^. Remarkably, a significant increase in the content of threonate was detected in NO-treated plants, thus suggesting NO is not triggering the oxidation of ascorbate but its catabolism to non antioxidant molecules.

Metabolic changes were especially relevant in the categories of peptides and amino acids, carbohydrates and lipids (Fig. 1a), thus suggesting NO is altering central processes of primary metabolism. Among these metabolic categories, changes in the lipidome have a signaling potential^41^ that is worth to note. The content of several phospholipids was significantly enhanced upon NO treatment (Fig. 4). The huge increases (5 to 13-fold) of lyso-lipids (Supplementary Table S1 and Fig. 4) suggested a strong activation of lipase activities at six hours. Consistently, we also saw similar patterns of increase for linoleate and linolenate (Supplementary Table S1 and Fig. 4), which are the two predominant PUFAs in Arabidopsis membranes. In turn, we did not detect palmitate, one of the main sn-1 position saturated fatty acids. We also saw somewhat elevated levels of two C20 PUFAs, dihomolinoleate (20:2n6) and dihomolinolenate (20:3n3 or 3n6). These fatty acids are reported to occur in Arabidopsis membranes in only trace amounts^42^. Their detection here could reflect the lipase action on trace lipids, or it could result from the enhanced elongase activity, either constitutively expressed or induced by NO action. Besides the increase in phospholipids, the NO-exposed plants had also elevated contents in the oxylipin molecules 13-HODE + 9-HODE and 9,10-hydroxyactadec-12(Z)-enoic acid (Supplementary Table S1), which are involved in development and responses to stress^43^.

Several data from the metabolomic analyses of plants exposed to NO suggested that one aspect of the global stress responses activated by NO is the promotion of cell death. The transient but strong increase in pheophorbide a content detected in NO-exposed plants (Supplementary Fig. S1b) is consistent with the promotion of cell death as previously reported^35^. Also consistent with the promotion of cell death, many cells of roots from NO-treated plants were permeable to propidium iodide showing strongly stained nuclei (Fig. 5a). Metabolomic data also pointed to the massive protein and nucleic acid degradation in plants exposed to NO, and these catabolic processes are usually activated under cell death conditions^44^,^45^. Although we found a pattern of progressive DNA degradation upon NO treatment, no DNA ladders were detected (Supplementary Fig. S1a), thus suggesting cell death is not apoptotic but more likely necrotic. Accordingly, we have detected cell death in plants by Evans blue staining at 6 h and 24 h after exposure to NO (Fig. 6c). It has been reported that cell death can be delayed by polyamines^46^, so the increase in polyamines detected in NO-treated plants (Fig. 3) may represent a strategy to attenuate NO-triggered cell death. Moreover, not only protein breakdown but also tyrosine nitration-based protein modification has been characterized as a marker of cell death signaling pathways^47^. Accordingly, we found a strong increase in tyrosine protein nitration after exposure of plants to NO (Fig. 2b). In animals models, the nitration of proteins often favors subsequent proteolytic degradation^48^,^49^ and, in plants, we have recently reported that nitration of ABA receptors triggers their inactivation, polyubiquitylation and subsequent proteasomal degradation^50^. Although more work is required to substantiate the connection between NO, protein nitration and protein breakdown, the work presented here supports it. On the other hand, the functional connection between NO and cell death is complex in animal systems being reported both pro- and anti-apoptotic activities^51^,^52^ likely depending on the concentration. Similarly in plants, NO has both promoting and suppressing effects on cell death, depending on cell type and redox status, as well as on the NO dose^53^. NO has been characterized as a key regulator of programmed cell death during self-incompatibility events essential to prevent self-fertilization^54^. Maize cells with suppressed hemoglobin, enhanced nitric oxide and reduced auxin levels are committed to die during embryogenesis^55^. Another support of NO triggering cell death might come from the increased metabolism of nucleic acids detected in NO-exposed plants (Supplementary Table S1), which runs in parallel with genomic DNA degradation (Fig. S1a) in cells that are committed to die. However, NO-exposed plants despite of displaying multiple metabolic and cellular symptoms of cell death by 6 h, were fully viable and actually undistinguishable from untreated plants by 24 h and, by 7 days after exposure to NO, 100% of the treated plants survived and grew normally (Fig. 6c). These data suggest NO only triggers cell death under natural stress conditions in a sharply localized way in places where the intracellular concentration is above a threshold level.

A small but statistically significant increase (between 1.2 and 1.5-fold) in a large collection of dipeptides was also detected by 6 h after NO treatment (Supplementary Table S1). The accumulation of dipeptides may be the result of the N-terminal dipeptide release from target proteins, a process that in mammals is associated to cell differentiation, the protection from cell death, and to have a role in the degradation of oligopeptides from proteins such as collagen^56^. However, the levels of all identified dipeptides were not significantly high by 24 h after NO treatment (Supplementary Table S1), thus indicating all dipeptides should be degraded without an increase in free amino acid content at that time point. Another reason for the accumulation of dipeptides could be related to a plant response directed to ameliorate the combined effects of NO and O_2_ in the cysteine branch of the N-terminal targeted proteolysis of certain protein substrates through the N-end rule pathway, a process that we have recently characterized as a sensor mechanism of NO in Arabidopsis^57^. It is well known that certain dipeptides inhibited the proteolysis of targeted proteins through the N-end rule pathway^58^, thus allowing us to posit that an increase in endogenous levels of dipeptides might be used for the plant to protect some key proteins from specific N-terminal directed proteolysis.

All together, data presented here suggest that NO likely triggers an array of responses to alleviate toxicity and cellular damage that includes massive but transient metabolic reprogramming involving metabolites of both primary and secondary metabolism.

## Methods

### Plant materials, growth conditions and NO treatment

Wild type Col-0 and the NO-deficient *nia1nia2noa1-2* mutant seeds of *Arabidopsis thaliana* were sown in moistened soil and grown under photoperiod cycles of 16 h day and 8 h night (long days, at 22 °C and 20 °C, respectively), under 150 μE m-2 s-1 cool-white fluorescent lamps and 60% relative humidity. Alternatively, surface sterilized seeds were sown after 4 d of stratification at 4 °C under darkness and grown in agar-supplemented Murashige and Skoog (MS) medium (Duchefa, Haarlem, The Netherlands) supplemented with 1% (w/v) sucrose.

The pulse of NO was performed by incubating plants for 5 min in a tightly sealed transparent box after injection of 300 ppm of pure NO gas (Linde AG, Germany).

### Cuticle permeability tests

The toluidine blue test was carried out by placing 10 μl droplets of a 0.025% (w/v) toluidine blue solution in potato dextrose broth (PDB) on the upper side surface of leaves. After 2 h leaves were washed gently with distilled water to remove excess of the toluidine blue solution, and then leaves were excised and photographed. For Calcofluor white staining, leaves were bleached in absolute ethanol overnight, equilibrated in 0.2 M phosphate buffer (pH 9) for 1 h, and incubated for 1 min in 0.5% (w/v) Calcofluor white in 0.2 M phosphate buffer (pH 9). Leaves were rinsed in phosphate buffer to remove excess of Calcofluor white and viewed under UV light.

### Staining for cell permeability, cell death, NO, superoxide and starch

To assess cell permeability, roots of 10 d-old seedlings were stained with propidium iodide by dipping into a 10 μg/ml solution for 10 min. Stained roots were visualized under confocal microscopy with excitation at 488 nm and emission at 598–650 nm range. Cell death was assayed by staining seedlings with 2% (w/v) Evans blue solution for 5 min and subsequent extensive washing. The endogenous levels of NO were assayed by staining with DAF-FM DA, and the NO-associated fluorescence was detected with a fluorescence Leica DM 5000B microscope as described^32^. Superoxide staining was performed by dipping seedlings in 0.2% (w/v) solution of nitroblue tetrazolium (NBT) in 50 mM sodium phosphate buffer (pH 7.5) overnight at room temperature and protected from light followed by bleaching with hot ethanol. Staining of starch granules was performed by bleaching 7d-old seedlings with 96% ethanol for 5 h and then by incubating bleached seedlings in Lugol’s staining solution (5.7 mM iodine, 43.4 mM KI, 0.2N HCl) for 30 min and further extensive overnight washing with water.

### Protein extraction and Western blot analyses

Seedlings frozen in liquid nitrogen were homogenized with a polytron in 0.1 M Tris-HCl buffer pH 8 containing 150 mM NaCl, 0.1% (v/v) NP-40 (Roche) and 1% (v/v) protease inhibitor cocktail (Sigma). Supernatants of 15 min centrifugation at 16000 × g and 4 °C, were quantified with Bradford reagent and separated under SDS-PAGE. Gels were either stained for total protein with Imperial reagent (Thermo Scientific) or semi-dry transferred to nitrocellulose membranes. After blocking membranes with TTBS buffer supplemented with either 5% (w/v) non-fat dried milk, 3% (w/v) Top-Block (Fluka) or 3% (w/v) bovine serum albumin, Western blots were performed by incubating membranes overnight at 4 °C in the following primary antibodies at the indicated dilution factor: monoclonal anti-3nitroY (Cayman Chemicals, 1:1000) and monoclonal anti-TMT (Thermo Scientific 1:1000). Polyclonal anti DE-ETIOLATED 3 (DET3) (kindly donated by David Alabadí, IBMCP Valencia Spain, at 1:10000) was used as loading control^59^. Secondary anti-rabbit or anti-mouse antibodies coupled to horseradish peroxidase (GE, 1:5000) and Supersignal WestPico Chemiluniscence reagents (Thermo Scientific) were used to visualize bands.

### DNA isolation and Southern blot

Genomic DNA was isolated with hexadecyltrimethylammonium bromide (CTAB)-containing buffer, and 15 μg were separated through 2% agarose gel, blotted onto positively charged nylon membranes, and Southern blot performed with a digoxigenin-labelled probe (DIG High Prime DNA Labeling and Detection Starter Kit II from Roche).

### Metabolomic analyses

The sample preparation process was carried out using the automated MicroLab STAR^®^ system from Hamilton Company. Recovery standards were added prior to the first step in the extraction process for quality Control (QC) purposes. Sample preparation was conducted by series of organic and aqueous extractions to remove the protein fraction while allowing maximum recovery of small molecules. The resulting extract was divided into two fractions; one for analysis by Liquid Chromatography (LC) and one for analysis by Gas Chromatography (GC). Samples were placed briefly on a TurboVap^®^ (Zymark) to remove the organic solvent. Each sample was then frozen, dried under vacuum and prepared for either LC/MS or GC/MS. Details on the methodology used for the metabolomic analyses is included in the Metabolomic Procedure sheet of Supplementary Table S1.

### Statistical analyses

For metabolomic analyses, following log transformation and imputation with minimum observed values for each compound, Welch’s two-sample t-test and ANOVA contrast were used to identify biochemicals that differed significantly between experimental groups. A Two-way ANOVA was also used to identify biochemicals exhibiting a significant time and treatment main effect and the interaction effect between these two variables. An estimate of the false discovery rate (q-value) was calculated to take into account the multiple comparisons. Statistical analyses are performed with the program “R” http://cran.r-project.org/.

## Additional Information

**How to cite this article**: León, J. *et al*. Nitric oxide triggers a transient metabolic reprogramming in Arabidopsis. *Sci. Rep.* **6**, 37945; doi: 10.1038/srep37945 (2016).

**Publisher’s note:** Springer Nature remains neutral with regard to jurisdictional claims in published maps and institutional affiliations.

## Supplementary Material

Supplementary Table and Figure

## Figures and Tables

**Table 1:** Statistical comparisons in the metabolomic analyses.

Welch’s Two Sample t-test	Total number of metabolites p ≤ 0.05	Metabolites up p ≤ 0.05	Metabolites down p ≤ 0.05	Total number of metabolites 0.05 < p < 0.1	Metabolites up 0.05 < p < 0.1	Metabolites down 0.05 < p < 0.1
MOCK 6/PRE	12	1	11	17	1	16
MOCK 24/PRE	24	2	22	22	4	18
NOx6/PRE	133	114	19	15	10	5
NOx24/PRE	55	17	38	29	7	22
ANOVA contrast						
NOx6/MOCK6	154	138	16	14	9	5
NOx24/MOCK24	29	18	11	17	7	10
MOCK24/MOCK6	27	16	11	17	6	11
NOx24/NOx6	155	12	143	9	2	7

PRE, Pre-treatment; MOCK6, Mock-treated; harvested after 6 h; MOCK24, Mock-treated harvested after 24 h; NOx6, Nitric oxide treatment harvested after 6 h; NOx24, Nitric oxide treatment harvested after 24 h. N = 6 biological independent replicates.

## References

[b1] Arc E., Galland M., Godin B., Cueff G. & Rajjou L. Nitric oxide implication in the control of seed dormancy and germination. Front. Plant Sci. 4, 346 (2013). 24065970 10.3389/fpls.2013.00346 PMC3777103

[b2] Beligni M. V. & Lamattina L. Nitric oxide stimulates seed germination and de-etiolation, and inhibits hypocotyl elongation, three light-inducible responses in plants. Planta 210, 215–221 (2000). 10664127 10.1007/PL00008128

[b3] Lozano-Juste J. & León J. Nitric oxide regulates DELLA content and PIF expression to promote photomorphogenesis in Arabidopsis. Plant Physiol. 156, 1410–1123 (2011). 21562334 10.1104/pp.111.177741 PMC3135954

[b4] He Y. . Nitric oxide represses the Arabidopsis floral transition. Science 305, 1968–1971 (2004). 15448272 10.1126/science.1098837

[b5] Tsai Y. C., Delk N. A., Chowdhury N. I. & Braam J. Arabidopsis potential calcium sensors regulate nitric oxide levels and the transition to flowering. Plant Signal. Behav. 2, 446–454 (2007). 19517005 10.4161/psb.2.6.4695 PMC2634334

[b6] Manjunatha G., Lokesh V. & Neelwarne B. Nitric oxide in fruit ripening: trends and opportunities. Biotechnol. Adv. 28, 489–499 (2010). 20307642 10.1016/j.biotechadv.2010.03.001

[b7] Liu F. & Guo F. Q. Nitric oxide deficiency accelerates chlorophyll breakdown and stability loss of thylakoid membranes during dark-induced leaf senescence in Arabidopsis. PLoS One 8(2), e56345 (2013). 23418559 10.1371/journal.pone.0056345 PMC3572010

[b8] Du J. . Nitric oxide induces cotyledon senescence involving co-operation of the NES1/MAD1 and EIN2-associated ORE1 signalling pathways in Arabidopsis. J. Exp. Bot. 65, 4051–4063 (2014). 24336389 10.1093/jxb/ert429 PMC4106434

[b9] Siddiqui M. H., Al-Whaibi M. H. & Basalah M. O. Role of nitric oxide in tolerance of plants to abiotic stress. Protoplasma 248, 447–455 (2011). 20827494 10.1007/s00709-010-0206-9

[b10] Arasimowicz-Jelonek M. & Floryszak-Wieczorek J. Nitric oxide: an effective weapon of the plant or the pathogen? Mol. Plant Pathol. 15, 406–416 (2014). 24822271 10.1111/mpp.12095 PMC6638900

[b11] Thomas D. D. Breathing new life into nitric oxide signaling: A brief overview of the interplay between oxygen and nitric oxide. Redox Biol. 5, 225–33 (2015). 26056766 10.1016/j.redox.2015.05.002 PMC4473092

[b12] Groβ F., Durner J. & Gaupels F. Nitric oxide, antioxidants and prooxidants in plant defence responses. Front. Plant Sci. 4, 419 (2013). 24198820 10.3389/fpls.2013.00419 PMC3812536

[b13] Astier J. & Lindermayr C. Nitric oxide-dependent posttranslational modification in plants: an update. Int. J. Mol. Sci. 13, 15193–15208 (2012). 23203119 10.3390/ijms131115193 PMC3509635

[b14] Hess D. T. & Stamler J. S. Regulation by S-nitrosylation of protein post-translational modification. J. Biol. Chem. 287, 4411–4418 (2012). 22147701 10.1074/jbc.R111.285742 PMC3281651

[b15] Guerra D. D. & Callis J. Ubiquitin on the move: the ubiquitin modification system plays diverse roles in the regulation of endoplasmic reticulum- and plasma membrane-localized proteins. Plant Physiol. 160, 56–64 (2012). 22730427 10.1104/pp.112.199869 PMC3440229

[b16] Skalska K., Miller J. S. & Ledakowicz S. Trends in NO(x) abatement: a review. Sci. Total Environ. 408, 3976–3989 (2010). 20580060 10.1016/j.scitotenv.2010.06.001

[b17] Pilegaard K. Processes regulating nitric oxide emissions from soils. Phil. Transac. Royal Soc. London. Ser. B, Biol. Sci. 368, 20130126 (2013). 10.1098/rstb.2013.0126 PMC3682746 23713124

[b18] Jaegle L., Steinberger L., Martin R. V. & Chance K. Global partitioning of NOx sources using satellite observations: Relative roles of fossil fuel combustion, biomass burning and soil emissions. Faraday Discus. 130, 407–423 (2005). 10.1039/b502128f 16161795

[b19] Gupta K. J., Fernie A. R., Kaiser W. M. & van Dongen J. T. On the origins of nitric oxide. Trends Plant Sci. 16, 160–168 (2011). 21185769 10.1016/j.tplants.2010.11.007

[b20] Mur L. A. . Nitric oxide in plants: an assessment of the current state of knowledge. AoB Plants 5, pls052 (2013). 10.1093/aobpla/pls052 PMC3560241 23372921

[b21] Correa-Aragunde N., Foresi N. & Lamattina L. Nitric oxide is a ubiquitous signal for maintaining redox balance in plant cells: regulation of ascorbate peroxidase as a case study. J. Exp. Bot. 66, 2913–2921 (2015). 25750426 10.1093/jxb/erv073

[b22] Noctor G., Lelarge-Trouverie C. & Mhamdi A. The metabolomics of oxidative stress. Phytochemistry 112, 33–53 (2015). 25306398 10.1016/j.phytochem.2014.09.002

[b23] Allan W. L., Simpson J. P., Clark S. M. & Shelp B. J. Gamma-hydroxybutyrate accumulation in Arabidopsis and tobacco plants is a general response to abiotic stress: putative regulation by redox balance and glyoxylate reductase isoforms. J. Exp. Bot. 59, 2555–2564 (2008). 18495640 10.1093/jxb/ern122 PMC2423657

[b24] Romero L. C., Aroca M. Á., Laureano-Marín A. M., Moreno I., García I. & Gotor C. Cysteine and cysteine-related signaling pathways in *Arabidopsis thaliana*. Mol. Plant 7, 264–276 (2014). 24285094 10.1093/mp/sst168

[b25] Noctor G. . Glutathione in plants: an integrated overview. Plant Cell Environ. 35, 454–484 (2012). 21777251 10.1111/j.1365-3040.2011.02400.x

[b26] Feussner I. & Wasternack C. The lipoxygenase pathway. Ann. Rev. Plant Biol. 53, 275–297 (2002). 12221977 10.1146/annurev.arplant.53.100301.135248

[b27] Green M. A. & Fry S. C. Vitamin C degradation in plant cells via enzymatic hydrolysis of 4-O-oxalyl-L-threonate. Nature 433, 83–87 (2005). 15608627 10.1038/nature03172

[b28] Szarka A., Tomasskovics B. & Bánhegyi G. The ascorbate-glutathione-α-tocopherol triad in abiotic stress response. Int. J. Mol. Sci. 13, 4458–4483 (2012). 22605990 10.3390/ijms13044458 PMC3344226

[b29] Hurlock A. K., Roston R. L., Wang K. & Benning C. Lipid trafficking in plant cells. Traffic 15, 915–932 (2014). 24931800 10.1111/tra.12187

[b30] Blokhina O., Virolainen E. & Fagerstedt K. V. Antioxidants, oxidative damage and oxygen deprivation stress: a review. Ann. Bot. 91, 179–194 (2003). 12509339 10.1093/aob/mcf118 PMC4244988

[b31] Yeats T. H. & Rose J. K. The formation and function of plant cuticles. Plant Physiol. 163, 5–20 (2013). 23893170 10.1104/pp.113.222737 PMC3762664

[b32] Lozano-Juste J. & León J. Enhanced abscisic acid-mediated responses in nia1nia2noa1-2 triple mutant impaired in NIA/NR- and AtNOA1-dependent nitric oxide biosynthesis in Arabidopsis. Plant Physiol. 152, 891–903 (2010). 20007448 10.1104/pp.109.148023 PMC2815865

[b33] Hörtensteiner S. Update on the biochemistry of chlorophyll breakdown. Plant Mol Biol. 82, 505–17 (2013). 22790503 10.1007/s11103-012-9940-z

[b34] Pruzinská A. . Chlorophyll breakdown in senescent Arabidopsis leaves: characterization of chlorophyll catabolites and of chlorophyll catabolic enzymes involved in the degreening reaction. Plant Physiol. 139, 52–63 (2005). 16113212 10.1104/pp.105.065870 PMC1203357

[b35] Hirashima M., Tanaka R. & Tanaka A. Light-independent cell death induced by accumulation of pheophorbide a in *Arabidopsis thaliana*. Plant Cell Physiol. 50, 719–29 (2009). 19273468 10.1093/pcp/pcp035

[b36] Zottini M., Costa A., De Michele R., Ruzzene M., Carimi F. & Lo Schiavo F. Salicylic acid activates nitric oxide synthesis in Arabidopsis. J Exp Bot. 58, 1397–1405 (2007). 17317674 10.1093/jxb/erm001

[b37] Mainz E. R. . Monitoring intracellular nitric oxide production using microchip electrophoresis and laser-induced fluorescence detection. Analytical Methods 4, 414–420 (2012).

[b38] Vandelle E. & Delledonne M. Peroxynitrite formation and function in plants. Plant Sci. 181, 534–539 (2011). 21893249 10.1016/j.plantsci.2011.05.002

[b39] Minocha R., Majumdar R. & Minocha S. C. Polyamines and abiotic stress in plants: a complex relationship. Front. Plant Sci. 5, 175 (2014). 24847338 10.3389/fpls.2014.00175 PMC4017135

[b40] Parsons H. T., Yasmin T. & Fry S. C. Alternative pathways of dehydroascorbic acid degradation *in vitro* and in plant cell cultures: novel insights into vitamin C catabolism. Biochem. J. 440, 375–383 (2011). 21846329 10.1042/BJ20110939

[b41] Hou Q., Ufer G. & Bartels D. Lipid signalling in plant responses to abiotic stress. Plant Cell Environ. 39, 1029–4108 (2016). 26510494 10.1111/pce.12666

[b42] Zhou X. R., Callahan D. L., Shrestha P., Liu Q., Petrie J. R. & Singh S. P. Lipidomic analysis of Arabidopsis seed genetically engineered to contain DHA. Front. Plant Sci. 5, 41 (2014). 25225497 10.3389/fpls.2014.00419 PMC4150447

[b43] Pohl C. H. & Kock J. L. Oxidized fatty acids as inter-kingdom signaling molecules. Molecules 19, 1273–1285 (2014). 24448067 10.3390/molecules19011273 PMC6270766

[b44] Araújo W. L., Tohge T., Ishizaki K., Leaver C. J. & Fernie A. R. Protein degradation-an alternative respiratory substrate for stressed plants. Trends Plant Sci. 16, 489–498 (2011). 21684795 10.1016/j.tplants.2011.05.008

[b45] Sakamoto W. & Takami T. Nucleases in higher plants and their possible involvement in DNA degradation during leaf senescence. J. Exp. Bot. 65, 3835–3843 (2014). 24634485 10.1093/jxb/eru091

[b46] Del Duca S., Serafini-Fracassini D. & Cai G. Senescence and programmed cell death in plants: polyamine action mediated by transglutaminase. Front. Plant Sci. 5, 120 (2014). 24778637 10.3389/fpls.2014.00120 PMC3985020

[b47] Franco M. C. & Estévez A. G. Tyrosine nitration as mediator of cell death. Cell. Mol. Life Sci. 71, 3939–3950 (2014). 24947321 10.1007/s00018-014-1662-8 PMC11113622

[b48] Palumbo A., Fiore G., Di Cristo C., Di Cosmo A. & d’Ischia M. NMDA receptor stimulation induces temporary alpha-tubulin degradation signalled by nitric oxide-mediated tyrosine nitration in the nervous system of *Sepia officinalis*. Biochem. Biophys. Res. Commun. 293, 1536–1543 (2002). 12054691 10.1016/S0006-291X(02)00392-3

[b49] Wang Y. Y., Lin S. Y., Chuang Y. H., Mao C. H., Tung K. C. & Sheu W. H. Protein nitration is associated with increased proteolysis in skeletal muscle of bile duct ligation-induced cirrhotic rats. Metabolism 59, 468–472 (2010). 19846167 10.1016/j.metabol.2009.07.035

[b50] Castillo M. C., Lozano-Juste J., González-Guzmán M., Rodriguez L., Rodriguez P. L. & León J. Inactivation of PYR/PYL/RCAR ABA receptors by tyrosine nitration may enable rapid inhibition of ABA signaling by nitric oxide in plants. Sci. Signal. 8(392), ra89 (2015). 26329583 10.1126/scisignal.aaa7981

[b51] Blaise G. A., Gauvin D., Gangal M. & Authier S. Nitric oxide, cell signaling and cell death. Toxicology 208, 177–192 (2005). 15691583 10.1016/j.tox.2004.11.032

[b52] Brüne B. Nitric oxide: NO apoptosis or turning it ON? Cell Death Differ. 10, 864–869 (2003). 12867993 10.1038/sj.cdd.4401261

[b53] Wang Y., Chen C., Loake G. J. & Chu C. Nitric oxide: promoter or suppressor of programmed cell death? Prot. Cell 1, 133–142 (2010). 10.1007/s13238-010-0018-x PMC4875162 21203983

[b54] Serrano I., Romero-Puertas M. C., Sandalio L. M. & Olmedilla A. The role of reactive oxygen species and nitric oxide in programmed cell death associated with self-incompatibility. J. Exp. Bot. 66, 2869–2876 (2015). 25750430 10.1093/jxb/erv083

[b55] Huang S., Hill R. D. & Stasolla C. Plant hemoglobin participation in cell fate determination. Plant Signal. Behavior 9, e29485 (2014). 10.4161/psb.29485 PMC4205130 25763627

[b56] Maes M. B., Scharpé S. & De Meester I. Dipeptidyl peptidase II (DPPII), a review. Clin. Chim. Acta 380, 31–49 (2007). 17328877 10.1016/j.cca.2007.01.024

[b57] Gibbs D. J. . Nitric oxide sensing in plants is mediated by proteolytic control of group VII ERF transcription factors. Mol. Cell 53, 369–379 (2014). 24462115 10.1016/j.molcel.2013.12.020 PMC3969242

[b58] Kitamura K. Inhibition of the Arg/N-end rule pathway-mediated proteolysis by dipeptide-mimetic molecules. Amino Acids 48, 235–243 (2016). 26334349 10.1007/s00726-015-2083-1

[b59] Duek P. D., Elmer M. V., van Oosten V. R. & Fankhauser C. The degradation of HFR1, a putative bHLH class transcription factor involved in light signaling, is regulated by phosphorylation and requires COP1. Curr Biol. 14, 2296–2301 (2004). 15620659 10.1016/j.cub.2004.12.026

