# Supplementary material for: Nitric oxide triggers a transient metabolic reprogramming in Arabidopsis
Source: Sci Rep. 2016 Nov 25;6:37945. doi: 10.1038/srep37945 (PMC5122866; doi:10.1038/srep37945)
Supplement: Supplementary Table and Figure [file srep37945-s1.pdf]

Supplementary Information

Title: Nitric oxide triggers a transient metabolic reprogramming in Arabidopsis

Authors: José León*, Álvaro Costa, Mari-Cruz Castillo

Supplementary Table S1

Supplementary Figure S1

Supplementary Table S1

Metabolomic procedure

LC/MS- and GC/MS-based analyses of the metabolome ol Arabidopsis thaliana seedlings

The sample preparation process was carried out using the automated MicroLab STAR® system from Hamilton Company. Recovery standards were added prior to the first step in the extraction process for quality Control (QC) purposes. Sample preparation was conducted by series of organic and aqueous extractions to remove the protein fraction while allowing maximum recovery of small molecules. The resulting extract was divided into two fractions; one for analysis by Liquid Chromatography (LC) and one for analysis by Gas Chromatography (GC). Samples were placed briefly on a TurboVap® (Zymark) to remove the organic solvent. Each sample was then frozen, dried under vacuum and prepared for either LC/MS or GC/MS.

The LC/MS portion of the platform was based on a Waters ACQUITY UPLC and a Thermo-Finnigan LTQ mass spectrometer, which consisted of an electrospray ionization (ESI) source and linear ion-trap (LIT) mass analyzer. The sample extract was split into two aliquots, dried, then reconstituted in acidic or basic LC-compatible solvents, each of which contained 11 or more injection standards at fixed concentrations. One aliquot was analyzed using acidic positive ion optimized conditions and the other using basic negative ion optimized conditions in two independent injections using separate dedicated columns. Extracts reconstituted in acidic conditions were gradient eluted using water and methanol both containing 0.1% Formic acid, while the basic extracts, which also used water/methanol, contained 6.5 mM ammonium bicarbonate. The MS analysis alternated between MS and data-dependent MS2 scans using dynamic exclusion. The Thermo-Finnigan LTQ-FT mass spectrometer had a linear ion-trap (LIT) front end and a Fourier transform ion cyclotron resonance (FT-ICR) mass spectrometer back end. For ions with counts greater than 2 million, an accurate mass measurement could be performed. Accurate mass measurements could be made on the parent ion as well as fragments. The typical mass error was less than 5 ppm. Ions with less than two million counts require fragmentation spectra (MS/MS) typically generated in data dependent manner or targeted MS/MS in the case of lower level signals.

The samples destined for GC/MS analysis were re-dried under vacuum desiccation for a minimum of 24 hours prior to being derivatized under dried nitrogen using bistrimethyl-silyl-triflouroacetamide (BSTFA). The GC column was 5% phenyl and the temperature ramp is from 40° to 300° C in a 16 minute period. Samples were analyzed on a Thermo-Finnigan Trace DSQ fast-scanning single-quadrupole mass spectrometer using electron impact ionization.

The data extraction of the raw mass spec data files yielded information that was loaded into a relational database and manipulated without resorting to BLOB manipulation. Peaks were identified using peak integration software, and component parts were stored in a separate and specifically designed complex data structure. Compounds were identified by comparison to library entries of more than 1000 commercially available purified standards. The combination of chromatographic properties and mass spectra gave an indication of a match to the specific compound or an isobaric entity. Additional entities could be identified by virtue of their recurrent nature (both chromatographic and mass spectral). A variety of curation procedures were carried out to ensure accurate and consistent identification of true chemical entities, and to remove those representing system artifacts, mis-assignments, and background noise.

Explanation

OrigScale Values are normalized in terms of raw area counts.
Note: for a single day run, this is equivalent to the raw data

ScaledImpData Each biochemical in OrigScale is re-scaled to have median equal to 1.
Missing values are imputed with the minimum.

Pathway Heat Map This is the heatmap associated with the statistical analysis of the data
Indicates ratios, p- and q-values for each comparison

0,55	**Green:** indicates significant difference (p≤0.05) between the groups shown; GREEN indicates a ratio < 1
0,55	**Light Green:** indicates differences approaching significance (0.05≤p≤0.1) between the groups shown; LIGHT GREEN indicates a ratio of <1
1,71	**Red:** indicates significant difference (p≤0.05) between the groups shown; RED indicates a ratio > 1
1,71	**Pink:** inidicates differences approaching signicance (0.05≤p≤0.1) between the groups shown; PINK indicates a ratio of >1
1,20	**Non-colored text and cell:** mean values are not significantly different for that comparison

* indicates compounds that have not been officially confirmed based on a standard, but we are confident in its identity

Box Plots-Alpha Box plots are provided for each biochemical detected, sorted alphabetically by biochemical name.

Box Plots-Pathway Box plots are provided for each biochemical detected, sorted by biochemical pathway.

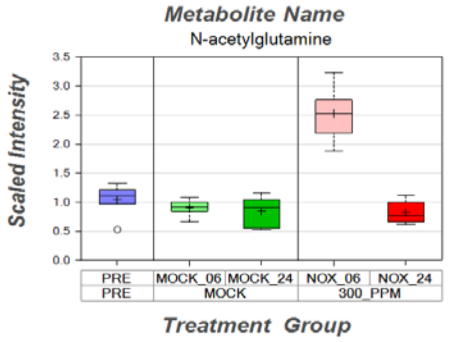

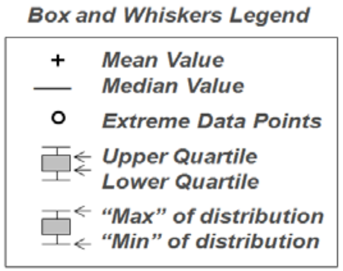

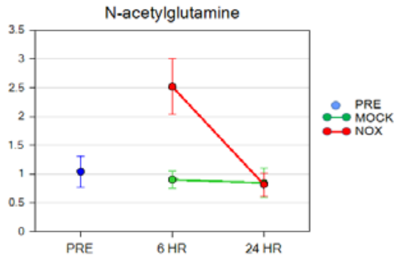

Pathway Heat Map

Heat map of statistically significant biochemicals profiled in this study. Red and green shaded cells indicate p≤0.05 (red indicates that the mean values are significantly higher for that comparison; green values significantly lower). Light red and light green shaded cells indicate 0.05<p<0.10 (light red indicates that the mean values trend higher for that comparison; light green values trend lower).

Pathway Order	Super Pathway	Sub Pathway	Biochemical Name	Platform	Comp ID	KEGG	HMDB	PUBCHEM	Fold of Change (Group Means Ratios in Scaled Imputed Data): Welch's Two sample t-test: MOCK 06 PRE				ANOVA Contrast: NOX 06 MOCK 06			
									MOCK 06 PRE	MOCK 24 PRE	NOX 06 PRE	NOX 24 PRE	NOX 06 MOCK 06	NOX 24 MOCK 24	MOCK 24 MOCK 06	NOX 24 NOX 06
7			**4-guanidinobutanoate**	LC/MS pos	15681	C01035	HMDB03464	500	0,87	0,82	2,32	1,09	2,67	1,33	0,94	0,47
9			**beta-hydroxypyruvate**	GC/MS	15686	C00168	HMDB01352	964	0,94	0,86	1,1	1,05	1,17	1,22	0,92	0,96
13			**cysteine**	GC/MS	31453	C00097	HMDB00574	5862;6419722	0,94	1,02	1,51	1,05	1,6	1,03	1,09	0,7
17		Serine family (phosphoglycerate derived)	**glycine**	GC/MS	11777	C00037	HMDB00123	5257127;750	1,04	1,32	1,67	1,19	1,6	0,9	1,26	0,71
25			**N-acetylserine**	LC/MS pos	37076		HMDB02931	65249	0,93	1,5	10,23	2,43	11,01	1,62	1,61	0,24
27			**O-acetylserine**	GC/MS	15947	C00979	HMDB03011	99478;6971051	0,87	0,97	14,87	1,45	17,11	1,49	1,12	0,1
28			**serine**	GC/MS	1648	C00065	HMDB00187	5951;6857581	0,87	1,04	1,03	1,1	1,19	1,06	1,2	1,07
76			**phenylalanine**	LC/MS pos	64	C00079	HMDB00159	6925665;6140	0,94	0,95	2,07	1,35	2,21	1,42	1,01	0,65
83		Aromatic amino acid metabolism (PEP derived)	**shikimate**	GC/MS	15144	C00493	HMDB03070	8742	0,99	0,91	0,72	0,62	0,73	0,68	0,92	0,85
85			**tryptophan**	LC/MS pos	54	C00078	HMDB00929	6923516;6305	0,97	0,99	2,61	1,73	2,69	1,75	1,02	0,66
87			**tyrosine**	LC/MS pos	1299	C00082	HMDB00158	6057;6942100	0,95	1	2,37	1,14	2,5	1,14	1,05	0,48
101			**alanine**	GC/MS	1126	C00041	HMDB00161	5950;7311724	1,07	1,02	1,68	0,99	1,57	0,96	0,96	0,59
103			**asparagine**	GC/MS	512	C00152	HMDB00168	6267;6992089	1,04	1,15	1,92	1,28	1,84	1,11	1,1	0,67
104			**aspartate**	GC/MS	15996	C00049	HMDB00191	5960	1,02	0,94	1,17	0,94	1,14	1	0,92	0,8
105			**beta-alanine**	GC/MS	55	C00099	HMDB00056	239;4755801	0,97	0,81	2,19	1,48	2,25	1,82	0,84	0,68
107			**cyano-alanine**	GC/MS	35660	C02512		13538	0,81	1,08	1,44	1,01	1,77	0,94	1,32	0,7
112		Aspartate family (OAA derived)	**homoserine**	GC/MS	23642	C00263	HMDB00719	12647;6971022	0,94	0,91	1,49	0,81	1,59	0,89	0,97	0,54
115			**lysine**	GC/MS	1301	C00047	HMDB00182	5962	0,95	1,08	2,46	1,38	2,6	1,28	1,14	0,56
116			**methionine**	GC/MS	1302	C00073	HMDB00696	6992087;6137	1,02	0,98	1,79	1,03	1,75	1,05	0,96	0,58
125			**N-acetylmethionine**	LC/MS neg	1589	C02712	HMDB11745	448580	1,08	0,9	0,86	1,13	0,8	1,26	0,83	1,31
134			**pipecolate**	GC/MS	1444	C00408	HMDB00070	849	0,75	0,46	1,23	0,34	1,63	0,74	0,61	0,28
138			**S-adenosylhomocysteine (SAH)**	LC/MS pos	39540	C00021	HMDB00939		0,98	0,62	0,77	0,68	0,78	1,09	0,64	0,89
141			**threonine**	GC/MS	1284	C00188	HMDB00167	6971019;6288	0,96	1,08	1,65	1,35	1,71	1,25	1,12	0,82
149			**2-aminobutyrate**	GC/MS	1577	C02261	HMDB00650	439691;6971251	0,87	0,99	1,33	0,99	1,53	1	1,14	0,74
150			**2-pyrrolidinone**	GC/MS	31675		HMDB02039	12025	0,82	0,97	1,46	0,49	1,78	0,5	1,19	0,33
152	Amino acid		**4-acetamidobutanoate**	GC/MS	1558	C02946	HMDB03681	18189	0,85	0,83	1,54	0,72	1,81	0,87	0,97	0,47
154			**4-hydroxybutyrate (GHB)**	GC/MS	34585	C00989	HMDB00710	10413	0,83	0,82	3,59	1,09	4,3	1,33	0,98	0,3
157			**arginine**	LC/MS pos	1638	C00062	HMDB00517	5246487;232	1,06	1,35	1,4	1,65	1,32	1,22	1,27	1,18
162			**citrulline**	LC/MS pos	2132	C00327	HMDB00904	833	0,97	0,89	1,26	0,93	1,3	1,04	0,92	0,74
166			**gamma-aminobutyrate (GABA)**	GC/MS	1416	C00334	HMDB00112	6992099;119	0,98	0,89	1,37	0,91	1,41	1,02	0,91	0,66
167			**glutamate**	GC/MS	57	C00025	HMDB03339	611	0,93	1,11	0,91	1,11	0,98	1,01	1,19	1,23
169		Glutamate family (alpha-ketoglutarate derived)	**glutamine**	LC/MS pos	53	C00064	HMDB00641	5961	1,05	1	1,25	1,12	1,2	1,12	0,96	0,9
171			**histidine**	LC/MS neg	59	C00135	HMDB00177	773;3651426	0,96	1,18	1,61	1,36	1,67	1,15	1,23	0,84
177			**N-acetylglutamine**	LC/MS pos	33943	C02716	HMDB06029	182230	0,87	0,81	2,42	0,79	2,79	0,97	0,94	0,33
179			**N-acetylornithine**	LC/MS pos	15630	C00437	HMDB03357	6992102;439232	0,91	1,02	5,3	4,34	5,83	4,26	1,12	0,82
180			**N-acetylproline**	LC/MS pos	34387			322640	0,89	1,04	1,55	1,04	1,74	1	1,16	0,67
181			**N-acetylputrescine**	LC/MS pos	37496	C02714	HMDB02064	122356	1,02	0,7	1,05	1,22	1,03	1,73	0,69	1,16
183			**ornithine**	GC/MS	1493	C00077	HMDB03374	6262	0,66	1,63	2,59	1,56	3,96	0,95	2,49	0,6
184			**proline**	LC/MS pos	1898	C00148	HMDB00162	145742;6971047	0,68	0,84	1,23	0,71	1,81	0,85	1,23	0,58
189			**trans-4-hydroxyproline**	GC/MS	1366	C01157	HMDB00725	5810;6971053	0,74	0,87	1,3	0,83	1,77	0,95	1,19	0,64
195			**4-hydroxy-2-oxoglutaric acid**	GC/MS	40062	C01127	HMDB02070	599	0,92	0,77	0,37	0,68	0,4	0,88	0,84	1,85
202		Branched Chain Amino Acids (OAA derived)	**isoleucine**	LC/MS pos	1125	C00407	HMDB00172	791	0,98	0,97	2,34	1,26	2,38	1,3	0,98	0,54
221		Branched Chain Amino Acids (pyruvate derived)	**leucine**	LC/MS pos	60	C00123	HMDB00687	7045798;6106	0,96	0,93	2,42	1,09	2,53	1,18	0,97	0,45
230			**valine**	LC/MS pos	1649	C00183	HMDB00883	6971018;6287	1,02	0,97	1,89	1,14	1,85	1,18	0,95	0,6
233			**5-methylthioadenosine (MTA)**	LC/MS pos	1419	C00170	HMDB01173	439176	1,1	0,96	1,06	0,84	0,96	0,87	0,88	0,8
234		Amines and polyamines	**agmatine**	GC/MS	15496	C00179	HMDB01432	199	0,73	1,14	2,01	1,17	2,75	1,02	1,56	0,58
237			**putrescine**	GC/MS	1408	C00134	HMDB01414		1,01	1,18	2,29	2,71	2,26	2,29	1,17	1,18
238			**spermidine**	GC/MS	485	C00315	HMDB01257	1102	1,02	1,01	1,43	1,08	1,4	1,07	0,99	0,75
240			**5-oxoproline**	LC/MS pos	1494	C01879	HMDB00267	7405	1,06	1,02	1,59	1,23	1,51	1,21	0,96	0,77
241		Glutathione metabolism	**cysteine-glutathione disulfide**	LC/MS pos	35159		HMDB00656	4247235	0,97	0,89	2,04	1,38	2,1	1,55	0,92	0,68
243			**glutathione, oxidized (GSSG)**	LC/MS pos	38783	C00127	HMDB03337	65359;11215652	1,02	0,9	1,57	1,11	1,54	1,23	0,89	0,71
252			**1,3-dihydroxyacetone**	GC/MS	35981	C00184	HMDB01882	670	0,94	0,96	1,41	1	1,49	1,05	1,02	0,71

262			glucose	GC/MS	20488	C00031	HMDB00122	79025	0,88	1,01	1,08	0,98	1,22	0,97	1,14	0,91
264		Glycolysis	glucose-6-phosphate (G6P)	GC/MS	31260	C00668	HMDB01401		0,95	0,94	0,54	0,72	0,57	0,77	0,99	1,33
266			glycerate	GC/MS	1572	C00258	HMDB00139	752	0,92	1,05	0,54	0,77	0,59	0,73	1,15	1,43
272			pyruvate	GC/MS	599	C00022	HMDB00243	107735	0,97	0,9	0,81	0,79	0,83	0,88	0,93	0,97
276			alpha-ketoglutarate	GC/MS	33453	C00026	HMDB00208	51	0,76	0,76	0,38	0,53	0,51	0,7	1	1,39
278			citrate	GC/MS	1564	C00158	HMDB00094	311	0,99	0,87	1,47	0,72	1,49	0,83	0,88	0,49
279		TCA cycle	fumarate	GC/MS	1643	C00122	HMDB00134		1,02	1	1,13	0,8	1,1	0,8	0,98	0,71
281			malate	GC/MS	1303	C00149	HMDB00156	525	0,99	0,81	0,66	0,68	0,67	0,84	0,82	1,03
285			succinate	GC/MS	1437	C00042	HMDB00254	1110	0,98	0,85	1,49	0,76	1,53	0,89	0,87	0,51
297		Calvin cycle and pentose phosphate	ribose 5-phosphate	GC/MS	561	C00117	HMDB00618	447634	0,95	1,42	0,83	0,84	0,87	0,59	1,5	1,02
300			sedoheptulose-7-phosphate	GC/MS	35649	C05382	HMDB01068	616	0,43	0,72	0,3	0,23	0,71	0,32	1,69	0,76
315			arabinose	GC/MS	575	C00216	HMDB00646	66308	0,84	1,04	1,61	1,04	1,93	1	1,25	0,65
317			arabitol	GC/MS	38075	C00474	HMDB01851	94154	1	32,47	1,7	1,43	1,7	0,04	32,52	0,84
318			arabonate	GC/MS	37516		HMDB00539	122045	1	1,2	1,54	1,14	1,54	0,95	1,2	0,74
320			erythritol	GC/MS	20699	C00503	HMDB02994		0,81	1,07	1,58	0,95	1,95	0,88	1,32	0,6
321			erythronate*	GC/MS	33477		HMDB00613	2781043	0,88	1,04	1,26	0,96	1,43	0,92	1,18	0,77
322			fucose	GC/MS	15821	C02095	HMDB00174	3034656	0,98	0,97	1,5	1,01	1,53	1,04	0,99	0,68
323			gluconate	GC/MS	587	C00257	HMDB00625	10690	0,9	0,89	2,36	0,96	2,63	1,08	0,99	0,41
326			glucosamine	GC/MS	18534	C00329	HMDB01514	441477	0,42	0,86	1,3	0,82	3,06	0,96	2,03	0,63
333		Amino sugar and nucleotide sugar	N-acetylglucosamine	GC/MS	15095	C00140	HMDB00215	24139	0,88	0,79	1,6	0,71	1,82	0,91	0,9	0,44
341	Carbohydrate		ribitol	GC/MS	15772	C00474	HMDB00508		0,9	1,38	1,38	1,03	1,53	0,74	1,53	0,74
343			ribose	GC/MS	12083	C00121	HMDB00283	5311110	0,97	0,95	1,45	0,84	1,5	0,89	0,98	0,58
345			ribulose	GC/MS	35855	C00309	HMDB00621	79021	1,06	1,36	1,29	0,73	1,22	0,54	1,29	0,56
346			threitol	GC/MS	35854	C16884	HMDB04136	169019	0,8	1,04	1,79	1,07	2,24	1,03	1,3	0,6
347			UDP-glucose	GC/MS	32344	C00029	HMDB00286	8629	0,9	1,06	0,8	0,8	0,88	0,76	1,17	1
353			xylonate	GC/MS	35638	C05411		6602431	0,92	1,07	1,47	0,96	1,6	0,89	1,16	0,65
354			xylose	GC/MS	15835	C00181	HMDB00098	95259	0,98	0,92	1,58	0,85	1,62	0,93	0,94	0,54
355			xylulose	GC/MS	18344	C00310	HMDB00654	5289590	1,04	1,36	6,66	4,95	6,4	3,64	1,31	0,74
362			inositol 1-phosphate (I1P)	GC/MS	1481	C04006	HMDB00213		0,85	0,94	1,02	0,78	1,19	0,84	1,1	0,77
363		Inositol metabolism	inositol 2-phosphate (I2P)	GC/MS	27725	C01177	HMDB01313 HMDB02985 HMDB0[illegible]		0,76	0,77	1,27	0,62	1,67	0,81	1,01	0,49
365			myo-inositol	GC/MS	19934	C00137	HMDB00211		0,87	0,93	1,13	0,79	1,29	0,84	1,07	0,7
379			3-deoxyoctulosonate	GC/MS	15942			4636210	0,97	0,87	1,13	0,86	1,17	0,99	0,89	0,76
391			fructose	GC/MS	577	C00095	HMDB00660	5984	0,79	1,04	1,11	1,04	1,4	1	1,31	0,93
392			galactinol	GC/MS	21034	C01235	HMDB05826		0,73	1,43	2,02	1,07	2,78	0,75	1,97	0,53
395			galactose	GC/MS	12055	C01582	HMDB00143	3037556	0,94	0,94	1,37	0,81	1,46	0,87	0,99	0,59
397			galacturonate	GC/MS	20758	C08348	HMDB02545	84740	0,98	0,95	1,34	0,86	1,37	0,9	0,97	0,64
418		Sucrose, glucose, fructose metabolism	mannitol	GC/MS	15335	C00392	HMDB00765	6251	0,28	0,21	0,3	0,4	1,06	1,87	0,76	1,34
422			mannose-6-phosphate	GC/MS	1469	C00275	HMDB01078		0,86	0,86	0,9	0,8	1,05	0,93	1	0,89
431			raffinose	LC/MS neg	586	C00492	HMDB03213	439242	0,63	1,12	0,81	0,72	1,28	0,64	1,77	0,89
432			rhamnose	GC/MS	15826	C00507	HMDB00849	19233	0,96	1,05	1,46	1,08	1,52	1,04	1,09	0,74
438			sucrose	LC/MS neg	1519	C00089	HMDB00258	5988	0,27	0,69	0,69	0,23	2,53	0,33	2,53	0,33
441			trehalose	GC/MS	15573	C01083	HMDB00975	7427	1,05	1,03	0,94	0,76	0,89	0,74	0,98	0,81
449			Isobar: 1-kestose, levan	LC/MS neg	40806				0,68	0,62	0,91	0,51	1,33	0,83	0,9	0,56
468			2-hydroxyglutarate	GC/MS	37253	C02630	HMDB00606	43	1,06	0,79	0,99	0,81	0,94	1,03	0,75	0,82
469			2-hydroxymyristate	LC/MS neg	32413	C13790	HMDB02261	1563	0,58	0,84	0,43	0,65	0,73	0,77	1,44	1,51
470			2-hydroxypalmitate	LC/MS neg	35675			92836	0,78	0,88	0,64	0,82	0,82	0,94	1,13	1,29
471			2-hydroxystearate	LC/MS neg	17945	C03045		69417	0,73	0,98	0,59	0,89	0,81	0,9	1,35	1,5
513		Free fatty acid	dihomo-linoleate (20:2n6)	LC/MS neg	17805	C16525		6439848	0,65	0,95	1,61	0,55	2,49	0,58	1,47	0,34
514			dihomo-linolenate (20:3n3 or n6)	LC/MS neg	35718	C03242	HMDB02925	5312529	0,74	0,59	1,46	0,7	1,98	1,18	0,8	0,48
522			eicosenoate (20:1n9 or 11)	LC/MS neg	33587		HMDB02231		0,74	0,75	0,95	0,71	1,28	0,94	1,02	0,75
539			linoleate (18:2n6)	LC/MS neg	1105	C01595	HMDB00673	5280450	0,94	0,79	3,51	0,8	3,73	1	0,84	0,23
540			linolenate [alpha or gamma; (18:3n3 or 6)]	LC/MS neg	34035	C06427	HMDB01388		1,06	0,79	4,34	0,79	4,1	1	0,75	0,18
541			malonate (propanedioate)	LC/MS neg	15872	C00383	HMDB00691	867	0,98	0,87	0,96	0,87	0,99	1	0,89	0,91
596		Oxylipins	13-HODE + 9-HODE	LC/MS neg	37752				0,91	0,76	2,3	0,93	2,54	1,23	0,83	0,4
600			9,10-hydroxyoctadec-12(Z)-enoic acid	LC/MS neg	38399	C14828	HMDB04704	9966640	1	1	2,12	1	2,12	1	1	0,47
624		Glycerolipids	1-linoleoylglycerol (1-monolinolein)	LC/MS neg	27447			5283469	1,09	1,12	2,58	1,11	2,37	0,99	1,03	0,43
648	Lipids		glycerol	GC/MS	15122	C00116	HMDB00131	753	0,93	0,9	1,32	0,84	1,42	0,94	0,96	0,64
653			1-linoleoylglycerophosphocholine	LC/MS pos	34419	C04100		11988421	0,82	0,94	8,82	0,74	10,75	0,79	1,15	0,08
654			1-linoleoylglycerophosphoethanolamine*	LC/MS neg	32635		HMDB11507		1,15	1,03	10,21	0,87	8,87	0,84	0,9	0,09
663			1-palmitoylglycerophosphocholine	LC/MS pos	33955			86554	1,08	0,91	9,01	1,18	8,3	1,29	0,84	0,13

664		Phospholipids	1-palmitoylglycerophosphoethanolamine	LC/MS neg	35631		HMDB11503	9547069	1,03	0,9	13,6	1,36	13,23	1,51	0,88	0,1
665			1-palmitoylglycerophosphoinositol*	LC/MS neg	35305				1,5	1,46	2,56	1,53	1,7	1,05	0,97	0,6
667			1-stearoylglycerophosphocholine	LC/MS pos	33961			497299	1,12	1,22	6,41	1,26	5,71	1,04	1,09	0,2
670			2-linoleoylglycerophosphocholine*	LC/MS pos	35257				1,02	1,27	4,85	1,13	4,76	0,89	1,25	0,23
671			2-linoleoylglycerophosphoethanolamine*	LC/MS neg	36593				0,72	0,62	3,89	0,64	5,38	1,04	0,85	0,16
679			glycerol 3-phosphate (G3P)	GC/MS	15365	C00093	HMDB00126	754	0,91	1,1	1,18	1,03	1,29	0,93	1,21	0,87
688			phosphoethanolamine	GC/MS	1600	C00346	HMDB00224	5232324;1015	0,87	0,78	0,69	1,16	0,79	1,48	0,9	1,68
697		Choline metabolism	choline phosphate	LC/MS pos	34396	C00588		135437	0,95	0,92	0,64	0,71	0,68	0,77	0,96	1,09
698			ethanolamine	GC/MS	1497	C00189	HMDB00149		1,05	0,92	1,09	1,16	1,04	1,26	0,88	1,06
717		Sterols	beta-sitosterol	GC/MS	27414	C01753	HMDB00852	222284	1,02	1,08	0,96	0,93	0,94	0,86	1,06	0,98
720			campesterol	GC/MS	39511	C01789	HMDB02869	173183	1,07	1,13	1,04	0,96	0,97	0,85	1,05	0,93
762	Cofactors, Prosthetic Groups, Electron Carriers	CoA metabolism	pantothenate	LC/MS pos	1508	C00864	HMDB00210	6613	0,85	0,88	1,13	0,97	1,33	1,1	1,04	0,86
777		Nicotinate and nicotinamide metabolism	nicotinamide ribonucleotide (NMN)	LC/MS pos	22152	C00455	HMDB00229	14180	1,1	0,89	0,68	0,88	0,62	0,98	0,81	1,28
778			nicotinamide riboside*	LC/MS pos	33013	C03150	HMDB00855		1,02	0,97	1,33	1,14	1,31	1,17	0,95	0,85
778,1			nicotianamine	GC/MS	43026	C05324	C05324	9882882	0,74	0,85	1,1	0,6	1,48	0,7	1,15	0,55
779			nicotinate	GC/MS	1504	C00253	HMDB01488	938	0,86	0,97	1,14	0,89	1,32	0,92	1,13	0,78
782			nicotinate ribonucleoside*	LC/MS pos	33471	C05841	HMDB06809	161233	0,96	0,89	1,57	0,97	1,64	1,08	0,93	0,62
797		Oxidative phosphorylation	methylphosphate	GC/MS	37070			13130	0,94	0,97	1,25	0,87	1,33	0,89	1,03	0,69
798			phosphate	GC/MS	11438	C00009	HMDB01429	1061	0,88	0,95	1,14	0,78	1,28	0,81	1,08	0,68
817		Riboflavin and FAD metabolism	riboflavin (Vitamin B2)	LC/MS pos	1827	C00255	HMDB00244	493570	1,03	1,02	1,38	0,89	1,34	0,87	0,99	0,64
818			flavin mononucleotide (FMN)	LC/MS neg	15797	C00061	HMDB01520	710	1	0,85	0,78	0,8	0,78	0,95	0,85	1,02
821		Quinone metabolism	phytonadione (Vitamin K1)	GC/MS	12250	C02059	HMDB03555	5284607	0,98	0,96	1,07	0,79	1,09	0,82	0,98	0,74
824		Ascorbate metabolism	ascorbate (Vitamin C)	GC/MS	1640	C00072	HMDB00044		0,89	0,78	0,8	0,71	0,9	0,9	0,88	0,89
825			dehydroascorbate	GC/MS	1659	C05422	HMDB01264	835	0,97	1,02	0,95	0,89	0,98	0,88	1,05	0,94
832			threonate	GC/MS	27738	C01620	HMDB00943	151152	0,9	0,81	3,85	0,69	4,29	0,85	0,9	0,18
839		Tocopherol metabolism	alpha-tocopherol	GC/MS	1561	C02477	HMDB01893	14985	0,92	1,11	0,51	0,92	0,55	0,83	1,2	1,81
848		Vitamin B metabolism (B6 or B12)	pyridoxal	LC/MS pos	1651	C00250	HMDB01545	1050	0,92	0,78	0,94	0,97	1,02	1,24	0,85	1,03
852			pyridoxate	LC/MS neg	31555	C00847	HMDB00017	6723	1,01	0,97	1,06	1,14	1,05	1,17	0,96	1,07
859		Chlorophyll and heme metabolism	pheophorbide A	LC/MS pos	35879	C18021			1,14	1,03	4,19	1,17	3,69	1,14	0,9	0,28
862			phytol	GC/MS	40140	C01389	HMDB02019	5280435	0,95	1	1,15	0,84	1,21	0,84	1,05	0,73
873	Nucleotide	Purine metabolism	2'-deoxyadenosine	LC/MS pos	1553	C00559	HMDB00101	13730	1,08	0,94	1,08	1,02	1	1,09	0,87	0,95
883			adenine	LC/MS pos	554	C00147	HMDB00034	190	0,97	0,88	1,48	0,94	1,53	1,07	0,91	0,64
884			adenosine	LC/MS pos	555	C00212	HMDB00050	60961	1,37	0,83	0,95	0,76	0,69	0,92	0,6	0,8
885			adenosine 2'-monophosphate (2'-AMP)	LC/MS neg	36815	C00946	HMDB11617		0,98	0,93	0,77	1,01	0,79	1,09	0,95	1,3
888			adenosine 3'-monophosphate (3'-AMP)	LC/MS neg	35142	C01367	HMDB03540	15938966	1,21	0,95	1,78	1,39	1,47	1,46	0,79	0,78
892			adenosine-2',3'-cyclic monophosphate	LC/MS pos	37467	C02353		2024	1,07	1,03	1,03	1,03	0,96	1	0,96	0,99
894			adenylosuccinate	LC/MS neg	18360	C03794	HMDB00536	195	1,17	1,16	1,23	0,82	1,05	0,7	0,99	0,67
896			allantoin	GC/MS	22808	C02350	HMDB00462	204	0,98	1,41	1,83	1,81	1,87	1,28	1,44	0,99
899			guanine	LC/MS pos	32352	C00242	HMDB00132	764	1,05	0,89	2,17	1,02	2,08	1,15	0,85	0,47
900			guanosine	LC/MS neg	1573	C00387	HMDB00133	6802	1,09	0,88	1,39	0,87	1,28	0,98	0,81	0,62
905			guanosine-2',3'-cyclic monophosphate	LC/MS pos	37139	C06194	HMDB11629	417655	1,07	0,94	1,22	1,03	1,14	1,1	0,88	0,84
908			inosine	LC/MS neg	1123	C00294	HMDB00195		1,17	0,88	0,63	0,93	0,54	1,06	0,75	1,48
917			urate	GC/MS	1604	C00366	HMDB00289		0,79	1,05	1,3	1,33	1,65	1,27	1,33	1,03
919			xanthosine	LC/MS neg	15136	C01762	HMDB00299	64959	0,95	1,06	0,83	1,24	0,87	1,17	1,11	1,49
946		Pyrimidine metabolism	cytidine	LC/MS pos	514	C00475	HMDB00089	6175	1,05	0,9	1,58	0,96	1,51	1,06	0,86	0,61
952			cytosine-2',3'-cyclic monophosphate	LC/MS pos	37465	C02354	HMDB11691	417654	1,05	0,99	1,55	1,25	1,49	1,26	0,94	0,8
959			pseudouridine	LC/MS pos	33442	C02067	HMDB00767		1,08	0,98	2,93	1,25	2,7	1,28	0,9	0,43
966			uracil	GC/MS	605	C00106	HMDB00300	1174	1,29	0,96	9,85	1,28	7,61	1,34	0,74	0,13
967			uridine	LC/MS neg	606	C00299	HMDB00296	6029	0,99	0,8	1,47	0,88	1,48	1,09	0,81	0,6
977		gamma-glutamyl	gamma-glutamylglutamate	LC/MS pos	36738			92865	1,08	0,76	0,71	0,79	0,66	1,04	0,7	1,11
991			valylglycine	LC/MS pos	40475			136487	1	0,78	1,33	0,87	1,33	1,12	0,78	0,65
1009			alanylisoleucine	LC/MS pos	37118			417358;5246008	0,91	0,86	1,02	0,8	1,11	0,92	0,95	0,79
1010			alanylleucine	LC/MS pos	37093			259583	0,92	0,9	1,29	0,78	1,4	0,87	0,98	0,61
1013			alanylvaline	LC/MS pos	37084			137276	0,88	0,86	0,99	0,81	1,12	0,94	0,97	0,82
1016,5			asparagylleucine	LC/MS pos	42980				0,93	0,82	1,55	0,78	1,66	0,95	0,88	0,51
1018			aspartylphenylalanine	LC/MS pos	22175		HMDB00706	93078	1,13	1,02	1,29	1,01	1,15	0,99	0,91	0,79
1037			glycylisoleucine	LC/MS pos	36659				1,12	1,01	1,7	1,04	1,51	1,03	0,9	0,61
1038			glycylleucine	LC/MS pos	34398	C02155	HMDB00759	92843;1548899	0,95	0,9	1,24	0,89	1,3	0,99	0,95	0,72
1045			glycyltyrosine	LC/MS pos	33958			92829;6994980	0,91	0,85	0,89	0,8	0,98	0,93	0,94	0,89
1047			glycylvaline	LC/MS pos	18357			2724807;6994979	0,99	0,88	1,31	0,88	1,33	1,01	0,89	0,68

1049			**isoleucylisoleucine**	LC/MS pos	36761				0,94	**0,74**	1,22	**0,64**	**1,3**	0,86	**0,78**	**0,52**
1050			**isoleucylphenylalanine**	LC/MS neg	40067			435728	1,08	1	**1,88**	0,96	**1,73**	0,96	0,92	**0,51**
1051			**isoleucylleucine**	LC/MS pos	36760			11644431	0,93	1,04	**1,55**	1	**1,67**	0,96	1,12	**0,64**
1052			**leucylisoleucine**	LC/MS pos	36757				0,99	0,86	1,16	1,02	1,17	1,19	0,86	0,88
1054			**valylaspartate**	LC/MS pos	40650			9964657	0,98	**0,78**	**1,19**	**0,88**	**1,22**	1,13	**0,79**	**0,74**
1055			**valylphenylalanine**	LC/MS neg	40688			6993119;6993120	1,08	**0,89**	**1,7**	1	**1,58**	1,12	**0,83**	**0,59**
1056			**valylvaline**	LC/MS neg	40728			409682;4280382	1,16	0,95	**1,51**	0,9	**1,3**	0,95	0,82	**0,6**
1057,5			**isoleucylaspartate**	LC/MS neg	42982				1,03	0,85	**1,77**	0,83	**1,73**	0,98	0,83	**0,47**
1059			**leucylleucine**	LC/MS pos	36756	C11332		76807;6992072	1,02	1,02	**1,52**	1,08	**1,48**	1,07	0,99	**0,71**
1061			**leucylaspartate**	LC/MS neg	40053			3328705	0,99	0,83	**1,37**	0,82	**1,39**	0,99	0,84	**0,6**
1066			**phenylalanylvaline**	LC/MS pos	41393			4096934	0,9	0,89	**1,45**	0,93	**1,61**	1,05	0,99	**0,64**
1071			**leucylglycine**	LC/MS pos	40045			79070	**0,87**	**0,89**	1,03	**0,77**	**1,19**	**0,87**	1,03	**0,75**
1073			**valylalanine**	LC/MS pos	41518			334517	0,99	0,95	**1,33**	0,94	**1,35**	0,99	0,96	**0,71**
1084			**arginylleucine**	LC/MS pos	39995			333445	**0,86**	0,93	1,05	0,96	**1,22**	1,03	1,09	0,91
1086	Peptide		**isoleucylglutamate**	LC/MS neg	40057				0,99	**0,79**	1,2	**0,7**	1,21	0,89	0,79	**0,58**
1088		Dipeptide	**isoleucylglycine**	LC/MS pos	40008			342532	0,9	0,89	**1,32**	**0,83**	**1,46**	0,93	0,98	**0,63**
1090			**isoleucylserine**	LC/MS neg	40012				0,87	**0,77**	1,13	**0,74**	**1,3**	0,96	0,89	**0,66**
1092,6			**threonylisoleucine**	LC/MS neg	42972				0,99	0,89	**1,5**	0,9	**1,52**	1,02	0,9	**0,6**
1093			**threonylphenylalanine**	LC/MS pos	31530			4099799;4099798	0,98	0,84	**1,23**	0,94	**1,26**	1,13	0,85	**0,77**
1094			**aspartylleucine**	LC/MS pos	40068			332962	0,95	**0,84**	**1,79**	**0,8**	**1,88**	0,96	0,88	**0,45**
1096			**isoleucylalanine**	LC/MS pos	40046			5246009;5246010	0,99	0,93	**1,47**	0,9	**1,49**	0,97	0,94	**0,61**
1098			**leucylalanine**	LC/MS pos	40010			259321	1,11	1,01	**1,48**	1,1	**1,33**	1,1	0,91	**0,75**
1099			**leucylasparagine**	LC/MS pos	40052			4128305	1,02	0,98	**1,35**	0,95	**1,32**	0,97	0,95	**0,71**
1100			**leucylglutamate**	LC/MS pos	40021			5259589;5259590	0,96	**0,78**	**1,26**	**0,77**	**1,32**	0,99	**0,81**	**0,61**
1102			**leucylserine**	LC/MS pos	40048			3621685	1,01	0,92	**1,25**	0,96	**1,24**	1,04	0,91	**0,77**
1103			**valylglutamate**	LC/MS pos	32454			7009623	0,92	**0,78**	1,3	**0,72**	**1,4**	0,92	0,85	**0,55**
1104			**lysylleucine**	LC/MS pos	40020			4682588	0,94	0,89	**1,31**	0,89	**1,39**	1	0,95	**0,68**
1105			**serylisoleucine***	LC/MS pos	40193				0,75	**0,67**	**1,43**	0,9	**1,9**	**1,34**	0,89	**0,63**
1106			**serylleucine**	LC/MS pos	40066				0,95	**0,86**	**1,29**	**0,86**	**1,36**	1	0,91	**0,66**
1107			**serylphenyalanine**	LC/MS pos	40054				**0,89**	**0,86**	**1,3**	**0,88**	**1,47**	1,02	0,97	**0,68**
1108			**threonylleucine**	LC/MS pos	40051			4420322	0,9	0,82	**1,5**	**0,81**	**1,66**	0,98	0,91	**0,54**
1119,2			**asparagylleucine**	LC/MS pos	42980				0,93	**0,82**	**1,55**	**0,78**	**1,66**	0,95	0,88	**0,51**
1120,1			**leucylthreonine**	LC/MS pos	42969				0,99	**0,8**	1,01	**0,89**	1,02	1,1	**0,81**	0,87
1121,04			**asparagylvaline**	LC/MS pos	42028				0,99	0,94	**1,34**	0,9	**1,36**	0,96	0,95	**0,67**
1121,19			**serylvaline**	LC/MS pos	42058				**0,88**	**0,73**	1,22	**0,84**	**1,39**	1,15	**0,83**	**0,69**
1121,23			**threonylvaline**	LC/MS pos	42011			416721;4278432	0,95	**0,8**	**1,31**	**0,82**	**1,38**	1,02	0,85	**0,63**
1121,31			**valylglutamine**	LC/MS pos	42079			5253209	1,08	0,91	**1,48**	1,02	**1,38**	1,12	0,85	**0,69**
1121,33			**valyllysine**	LC/MS neg	41384			5253210	1,03	1	**1,63**	0,9	**1,59**	0,89	0,98	**0,55**
1123,3			**glutamine-isoleucine**	LC/MS pos	42988				1,05	**0,82**	1,19	**0,8**	1,13	0,98	**0,78**	**0,68**
1123,4			**glutamine-leucine**	LC/MS pos	42994				0,96	**0,82**	**1,27**	**0,84**	**1,33**	1,02	0,86	**0,66**
1123,5			**threonylisoleucine**	LC/MS neg	42972				0,99	0,89	**1,5**	0,9	**1,52**	1,02	0,9	**0,6**
1130	Hormone metabolism	Auxin metabolism	**3-indolylacetonitrile**	LC/MS pos	21179	C02938	HMDB06524	351795	1	0,88	**0,71**	1,01	**0,71**	1,15	0,88	**1,43**
1174		Amine derived	**tyramine**	LC/MS pos	1603	C00483	HMDB00306	5610	0,94	**0,84**	1,03	0,81	1,09	0,97	0,89	**0,79**
1193		Benzenoids	**benzoyl-O-glucose**	LC/MS pos	38302			314421	0,88	0,9	1,17	0,88	**1,33**	0,98	1,02	**0,76**
1206		Fatty acid and sugar derivatives	**galactarate (mucic acid)**	GC/MS	20881	C00879	HMDB00639	3037582	**0,76**	0,89	1,03	0,72	**1,36**	0,81	1,17	**0,69**
1245			**dihydroquercetin**	LC/MS neg	38179	C12316		471	1	1	1	1	1	1	1	1
1249			**kaempferol**	GC/MS	17785	C05903	HMDB05801	5280863	0,68	0,74	0,77	0,79	1,14	1,07	1,08	1,02
1250		Flavonoids	**kaempferol 3-O-beta-glucoside**	LC/MS neg	38145				**0,76**	0,93	1,06	1,02	**1,39**	1,09	1,22	0,96
1265			**quercitrin**	LC/MS neg	21186	C01750		5280459	0,66	0,73	0,99	0,63	1,49	0,87	1,09	0,64
1273			**kaempferol-3-rhamnoside**	LC/MS neg	40041			5835713	0,97	0,78	0,78	0,76	**0,81**	0,97	0,8	0,97
1280	Secondary metabolism		**sulforaphane**	LC/MS pos	38697		HMDB05792	5350	0,95	0,89	**1,45**	0,82	**1,52**	0,92	0,94	**0,57**
1281			**sulforaphane-cysteine-glycine**	LC/MS pos	40412				1,03	0,8	1,05	1,08	1,03	1,34	0,78	1,02
1282		Glucosinolate	**3-methylsulfinylpropyl isothiocyanate**	LC/MS pos	39585			10455	0,91	0,9	**1,51**	**0,74**	**1,66**	0,81	0,99	**0,49**
1284			**sulforaphane-glutathione**	LC/MS pos	40422				1,1	0,83	**1,52**	1,38	1,39	1,67	0,76	0,91
1287,1			**4-methylsulfinylbutyl glucosinolate**	LC/MS neg	42571			9548634	0,92	0,88	**0,51**	0,78	**0,56**	0,89	0,95	**1,53**
1306			**coniferyl alcohol**	GC/MS	34696	C00590		1549095	0,65	0,78	0,85	0,87	1,32	1,11	1,21	1,02
1320		Phenylpropanoids	**sinapate**	GC/MS	21150	C00482		637775	0,93	0,97	0,55	0,78	**0,59**	0,8	1,04	1,41
1327,3			**pinoresinol**	LC/MS neg	42963	C10872		73399	0,87	0,85	0,87	0,81	0,99	0,96	0,97	0,94
1327,4			**lariciresinol**	LC/MS neg	43013	C10646		332427	0,81	0,86	0,88	0,85	1,08	0,99	1,05	0,97
1446	Xenobiotics	Chemicals	**trizma acetate**	GC/MS	20710	C07182		81291;16218782	**0,27**	1,1	1,17	0,99	4,26	0,89	**4,02**	0,84

Statistical Values (using log transformed scaled imputed data)															
MOCK 06 PRE		MOCK 24 PRE		NOX 06 PRE		NOX 24 PRE		NOX 06 MOCK 06		NOX 24 MOCK 24		MOCK 24 MOCK 06		NOX 24 NOX 06	
p-value	q-value	p-value	q-value	p-value	q-value	p-value	q-value	p-value	q-value	p-value	q-value	p-value	q-value	p-value	q-value
0,2599	0,7686	0,0825	0,2611	0,0017	0,0022	0,7414	0,411	9,44E-06	1,85E-05	0,1355	0,5389	0,7358	0,6783	0,0001	0,0002
0,6528	0,7761	0,3307	0,3883	0,5106	0,1117	0,8379	0,4359	0,3299	0,0863	0,2429	0,6677	0,5796	0,6119	0,7237	0,2064
0,4591	0,7696	0,8952	0,5588	0,0738	0,0235	0,6206	0,3643	0,0064	0,0029	0,828	0,9936	0,5806	0,6119	0,0351	0,014
0,8789	0,7761	0,1124	0,29	0,2494	0,0616	0,1909	0,1761	0,1706	0,0489	0,7029	0,9895	0,2724	0,5296	0,5044	0,1502
0,8237	0,7761	0,0652	0,2611	5,72E-06	4,78E-05	0,0035	0,0382	1,48E-10	3,48E-09	0,0277	0,2663	0,0136	0,206	1,15E-06	5,81E-06
0,1279	0,6095	0,7088	0,5217	9,08E-06	4,99E-05	0,0125	0,0549	8,80E-14	4,47E-12	0,0239	0,2617	0,4591	0,5974	3,43E-12	2,09E-10
0,0615	0,5869	0,5567	0,4848	0,8592	0,1694	0,4324	0,2986	0,106	0,0318	0,6644	0,9895	0,0648	0,3272	0,491	0,1469
0,3194	0,7686	0,3185	0,3883	9,08E-06	4,99E-05	0,0033	0,0382	1,07E-09	9,08E-09	0,0001	0,0115	0,8028	0,6839	1,27E-05	3,39E-05
0,8856	0,7761	0,3329	0,3883	0,0178	0,0079	0,0001	0,0039	0,0058	0,0026	0,0017	0,0571	0,4536	0,5974	0,212	0,072
0,6473	0,7761	0,8485	0,5501	3,94E-06	4,33E-05	8,41E-06	0,0009	2,05E-10	3,48E-09	1,58E-06	0,0004	0,818	0,6839	0,0001	0,0001
0,3579	0,7696	0,9591	0,5779	2,39E-05	8,65E-05	0,0893	0,13	8,97E-10	9,08E-09	0,1359	0,5389	0,5655	0,6119	3,54E-08	4,30E-07
0,2931	0,7686	0,7245	0,5258	3,20E-05	0,0001	0,8192	0,4303	2,89E-06	7,57E-06	0,6	0,954	0,5314	0,6008	2,58E-07	2,24E-06
0,6203	0,7696	0,2725	0,3832	0,0134	0,0065	0,2651	0,2064	0,0053	0,0025	0,7046	0,9895	0,6316	0,6323	0,0356	0,0142
0,6904	0,7761	0,4326	0,4169	0,162	0,0427	0,4445	0,3049	0,1503	0,0433	0,9778	1	0,266	0,5295	0,0167	0,0073
0,8243	0,7761	0,2828	0,3836	0,0024	0,0026	0,0232	0,0676	0,0002	0,0002	0,0022	0,0571	0,4	0,5816	0,058	0,0222
0,057	0,5869	0,3146	0,3883	0,0114	0,0058	0,791	0,4266	0,0005	0,0004	0,4552	0,8512	0,045	0,2929	0,0112	0,0052
0,6597	0,7761	0,5199	0,4658	0,0884	0,0268	0,1717	0,1676	0,0183	0,0068	0,4689	0,8663	0,8571	0,6839	0,0023	0,0014
0,4173	0,7696	0,3843	0,4053	0,0001	0,0003	0,0698	0,1143	5,18E-07	1,76E-06	0,1076	0,5048	0,3316	0,5543	0,0002	0,0002
0,8109	0,7761	0,7501	0,5305	9,51E-05	0,0003	0,9628	0,4726	6,40E-05	9,03E-05	0,7838	0,9936	0,7116	0,6783	5,13E-05	8,20E-05
0,3265	0,7686	0,2281	0,3676	0,1435	0,0392	0,0998	0,1302	0,0036	0,0017	0,0032	0,073	0,0167	0,206	0,0006	0,0005
0,5238	0,7696	0,0398	0,2361	0,7333	0,1505	0,0095	0,0496	0,2191	0,061	0,3171	0,7597	0,0414	0,2881	0,0002	0,0002
0,8281	0,7761	0,0014	0,0966	0,0112	0,0058	0,0081	0,0448	0,0537	0,0173	0,4995	0,8672	0,0008	0,1058	0,2414	0,081
0,5571	0,7696	0,3346	0,3883	2,48E-06	3,64E-05	0,0149	0,0558	3,40E-06	7,57E-06	0,0201	0,2565	0,1998	0,4677	0,0215	0,0091
0,2491	0,7686	0,8632	0,5501	0,0328	0,0123	0,9332	0,4678	0,0026	0,0014	0,903	0,9978	0,3664	0,5611	0,0269	0,0112
0,4152	0,7696	0,7697	0,5305	0,1508	0,0409	0,0163	0,0558	0,0452	0,0153	0,0625	0,4354	0,5936	0,6119	0,0019	0,0012
0,3479	0,7686	0,2477	0,3676	0,0195	0,0082	0,0592	0,1049	0,0129	0,0052	0,6833	0,9895	0,8265	0,6839	0,0084	0,0041
0,6995	0,7761	0,6511	0,511	0,0295	0,0113	0,8524	0,4393	0,0036	0,0017	0,5568	0,9148	0,9513	0,7164	0,012	0,0055
0,5383	0,7696	0,089	0,2611	0,1298	0,0361	0,0195	0,0611	0,2032	0,0571	0,2858	0,7145	0,22	0,4776	0,3074	0,1004
0,7555	0,7761	0,3528	0,3936	0,6722	0,1401	0,474	0,3166	0,5409	0,1323	0,8646	0,9936	0,5661	0,6119	0,314	0,102
0,6942	0,7761	0,1093	0,29	0,0235	0,0095	0,2087	0,1795	0,0068	0,003	0,8711	0,9936	0,5159	0,599	0,0022	0,0013
0,3929	0,7696	0,2768	0,3836	0,246	0,0611	0,1665	0,1676	0,7525	0,1786	0,8834	0,996	0,0331	0,2705	0,0121	0,0055
0,6232	0,7696	0,8984	0,5588	0,2942	0,0696	0,1999	0,1784	0,3236	0,0851	0,38	0,7946	0,7186	0,6783	0,6366	0,1859
0,4589	0,7696	0,0249	0,2265	0,0028	0,0027	0,0294	0,0687	0,0001	0,0001	0,2645	0,6931	0,0649	0,3272	0,1038	0,038
0,4735	0,7696	0,293	0,3839	0,0004	0,0007	0,2094	0,1795	4,73E-07	1,72E-06	0,9449	0,9978	0,4887	0,5974	1,02E-07	1,04E-06
0,2989	0,7686	0,9928	0,5904	0,1598	0,0426	0,0006	0,0158	0,027	0,0096	0,002	0,0571	0,7915	0,6839	0,1664	0,0571
0,3047	0,7686	0,7053	0,5217	0,0106	0,0056	0,8084	0,4284	0,0002	0,0002	0,937	0,9978	0,2283	0,4892	0,0035	0,002
0,9853	0,803	0,0794	0,2611	0,8364	0,1672	0,3558	0,2553	0,8393	0,192	0,0278	0,2663	0,1255	0,4123	0,3391	0,1067
0,0193	0,5212	0,3879	0,4058	0,1236	0,0353	0,4481	0,3049	0,0104	0,0043	0,9185	0,9978	0,0682	0,3272	0,3267	0,1039
0,0064	0,3993	0,1915	0,334	0,3158	0,0739	0,1108	0,1396	0,0263	0,0094	0,3025	0,744	0,4638	0,5974	0,0135	0,0061
0,0017	0,3067	0,1668	0,3188	0,0778	0,0244	0,2181	0,1819	0,003	0,0015	0,491	0,8663	0,3273	0,5527	0,0059	0,0031
0,5034	0,7696	0,1955	0,334	0,003	0,0028	0,0325	0,0739	0,001	0,0007	0,8582	0,9936	0,3147	0,5527	0,016	0,0071
0,7387	0,7761	0,5644	0,4855	2,34E-05	8,65E-05	0,0276	0,0687	3,21E-09	2,34E-08	0,0076	0,1255	0,84	0,6839	5,37E-07	3,26E-06
0,4103	0,7696	0,2506	0,3676	0,0005	0,0008	0,2302	0,1886	3,47E-08	1,97E-07	0,1319	0,5389	0,727	0,6783	3,62E-07	2,44E-06
0,6092	0,7696	0,4009	0,4099	2,54E-07	1,12E-05	0,0933	0,13	8,34E-10	9,08E-09	0,0131	0,1885	0,3698	0,5611	1,77E-08	3,59E-07
0,2874	0,7686	0,7637	0,5305	0,5108	0,1117	0,1042	0,133	0,579	0,1394	0,0682	0,4437	0,067	0,3272	0,0036	0,002
0,152	0,6838	0,8108	0,5428	0,0092	0,0053	0,4758	0,3166	0,0007	0,0005	0,7868	0,9936	0,1009	0,3729	0,0584	0,0222
0,9185	0,7828	0,6197	0,4979	0,0002	0,0004	2,15E-05	0,0012	0,0001	0,0002	4,97E-05	0,0057	0,5919	0,6119	0,3223	0,1036
0,8845	0,7761	0,9654	0,5792	0,0336	0,0124	0,485	0,3188	0,0299	0,0104	0,5347	0,8931	0,8586	0,6839	0,0738	0,0277
0,3428	0,7686	0,8492	0,5501	0,0068	0,0045	0,0513	0,1005	0,0012	0,0007	0,084	0,4602	0,6799	0,657	0,0269	0,0112
0,777	0,7761	0,2851	0,3836	0,0001	0,0003	0,0068	0,0439	2,23E-07	1,03E-06	0,0002	0,0126	0,4721	0,5974	0,0009	0,0006
0,8087	0,7761	0,4834	0,4532	0,0085	0,0052	0,4168	0,2896	0,0009	0,0006	0,0923	0,4805	0,326	0,5527	0,0052	0,0028
0,6842	0,7761	0,791	0,5362	0,0236	0,0095	0,9789	0,4755	0,0024	0,0013	0,7534	0,9936	0,9126	0,6974	0,0065	0,0034

Mean Values (Scaled Imputed Data)					% Detected by Group in RAW Data				
PRE	MOCK 06	MOCK 24	NOX 06	NOX 24	PRE	MOCK 06	MOCK 24	NOX 06	NOX 24
1,058	0,9218	0,8637	2,458	1,1499	100	100	100	100	100
1,0963	1,0307	0,9439	1,204	1,1554	100	100	100	100	100
0,9364	0,8789	0,9562	1,4094	0,9803	100	100	100	83	100
0,8818	0,921	1,1597	1,4728	1,046	100	100	100	100	100
0,6668	0,6196	0,9986	6,8192	1,6173	100	83	100	100	100
0,9138	0,7942	0,888	13,5866	1,321	100	100	100	100	100
1,0533	0,9113	1,096	1,0858	1,1568	100	100	100	100	100
0,9615	0,9022	0,9148	1,9926	1,3024	100	100	100	100	100
1,1567	1,1462	1,0559	0,8364	0,7143	100	100	100	100	100
0,8734	0,8467	0,8636	2,2794	1,5138	100	100	100	100	100
0,9534	0,9053	0,9545	2,264	1,0874	100	100	100	100	100
0,955	1,0218	0,9772	1,6086	0,9427	100	100	100	100	100
0,8608	0,8986	0,9875	1,6495	1,0991	100	100	100	100	100
0,9974	1,0199	0,9341	1,165	0,9358	100	100	100	100	100
0,8328	0,8102	0,6779	1,8233	1,235	100	100	100	100	100
0,9776	0,7944	1,0521	1,4087	0,9842	100	100	100	100	100
1,0874	1,0186	0,9919	1,6235	0,882	100	100	100	100	100
0,9457	0,8952	1,0203	2,3287	1,3051	100	100	100	100	100
0,9533	0,9722	0,9351	1,7046	0,9862	100	100	100	100	100
0,9363	1,0069	0,8391	0,8051	1,058	100	100	83	33	100
1,5569	1,1686	0,7113	1,9083	0,529	100	100	100	100	100
1,2638	1,2411	0,7889	0,9671	0,8566	100	100	100	100	100
0,8901	0,8582	0,962	1,4671	1,2018	100	100	100	100	100
0,9228	0,8035	0,9126	1,2292	0,9107	100	83	100	100	100
1,2817	1,0497	1,2453	1,8656	0,6231	100	100	100	100	100
1,0998	0,9379	0,9121	1,6987	0,7909	100	100	100	100	100
0,9131	0,7608	0,7457	3,2751	0,9928	100	83	83	100	83
0,865	0,9183	1,1686	1,2097	1,4263	100	100	100	100	100
1,1477	1,1116	1,0243	1,4454	1,0666	100	100	100	100	100
1,0401	1,0146	0,9225	1,4283	0,9423	100	100	100	100	100
1,0038	0,9334	1,1092	0,9104	1,1161	100	100	100	100	100
1,0081	1,0535	1,0088	1,264	1,1327	100	100	100	100	100
0,8741	0,8433	1,0341	1,4096	1,1849	100	100	100	100	100
1,0399	0,9021	0,8444	2,5178	0,8184	100	100	100	100	100
0,9053	0,8222	0,922	4,7944	3,9261	100	100	100	100	100
0,9505	0,848	0,9842	1,4716	0,9851	100	100	100	100	100
1,2042	1,2282	0,8458	1,2602	1,4632	100	100	83	100	100
0,9528	0,6245	1,5551	2,4719	1,483	100	100	100	100	100
1,0996	0,7457	0,9197	1,35	0,7834	100	100	100	100	100
1,1324	0,8331	0,9907	1,4739	0,9411	100	100	100	100	100
1,3074	1,2011	1,0102	0,4799	0,8899	100	100	100	100	100
0,9812	0,9655	0,9508	2,2991	1,2353	100	100	100	100	100
0,9997	0,9571	0,9259	2,4217	1,0893	100	100	100	100	100
0,9601	0,9806	0,9305	1,8176	1,0935	100	100	100	100	100
1,0242	1,1223	0,9871	1,0814	0,861	100	100	100	100	100
0,9637	0,706	1,0988	1,9403	1,1251	100	100	100	100	100
0,7359	0,7446	0,8693	1,6856	1,9922	100	100	100	100	100
0,9414	0,9602	0,9479	1,3463	1,016	100	100	100	100	100
0,9338	0,9863	0,9479	1,4881	1,1497	100	100	100	100	100
0,9117	0,8838	0,8154	1,8568	1,262	100	100	100	100	100
0,9772	0,997	0,8829	1,5383	1,0889	100	100	100	100	100
0,9376	0,8834	0,9005	1,3189	0,9421	100	100	100	100	100

0,0966	0,5869	0,8933	0,5588	0,217	0,0548	0,7911	0,4266	0,0086	0,0037	0,6139	0,954	0,0761	0,3369	0,1353	0,0483	1	0,8836	1,008	1,0788	0,9807	100	100	100	100	100
0,8233	0,7761	0,7743	0,5305	0,029	0,0112	0,093	0,13	0,0052	0,0024	0,1645	0,6201	0,9602	0,7199	0,1165	0,0421	1,2583	1,1946	1,1853	0,6856	0,909	100	100	100	100	100
0,3288	0,7686	0,8963	0,5588	1,99E-05	8,65E-05	0,0952	0,13	0,0027	0,0014	0,0467	0,3701	0,4561	0,5974	0,0527	0,0205	1,1713	1,0722	1,2282	0,6275	0,9	100	100	100	100	100
0,8204	0,7761	0,3669	0,3975	0,0745	0,0236	0,0352	0,0772	0,0748	0,0234	0,2459	0,6677	0,3821	0,5611	0,8361	0,2288	1,1652	1,1335	1,0525	0,9457	0,921	100	100	100	100	100
0,2055	0,7469	0,2821	0,3836	0,006	0,0043	0,0163	0,0558	0,0944	0,0287	0,8679	0,9936	0,5345	0,6008	0,3508	0,1093	1,555	1,1797	1,182	0,5961	0,8289	100	100	100	100	100
0,8134	0,7761	0,1643	0,3188	0,0329	0,0123	0,0068	0,0439	0,0088	0,0037	0,1871	0,6208	0,3702	0,5611	4,49E-05	7,37E-05	1,1428	1,1264	0,9913	1,6779	0,8246	100	100	100	100	100
0,8939	0,7761	0,9572	0,5779	0,194	0,0499	0,0793	0,1281	0,3659	0,0948	0,072	0,4437	0,8614	0,6839	0,007	0,0036	1,0222	1,0462	1,0236	1,155	0,822	100	100	100	100	100
0,8525	0,7761	0,0776	0,2611	0,0008	0,0012	0,0072	0,0439	0,003	0,0015	0,1445	0,5634	0,089	0,3664	0,9469	0,2534	1,2943	1,2766	1,0425	0,855	0,8764	100	100	100	100	100
0,7005	0,7761	0,0192	0,2265	0,0223	0,0092	0,0021	0,0327	0,0017	0,001	0,3293	0,7597	0,282	0,5355	1,34E-05	3,39E-05	1,0934	1,0667	0,9249	1,6293	0,8274	100	100	100	100	100
0,8767	0,7761	0,146	0,312	0,4726	0,1066	0,5606	0,346	0,5032	0,1253	0,0388	0,3432	0,0942	0,3664	0,8228	0,2262	0,8585	0,8141	1,2221	0,711	0,7227	67	100	100	50	67
0,0563	0,5869	0,4331	0,4169	0,0091	0,0053	0,0018	0,0322	0,4419	0,1111	0,0072	0,1255	0,1031	0,3729	0,6251	0,1834	1,9966	0,8526	1,4429	0,6019	0,4596	100	83	100	83	67
0,0955	0,5869	0,6744	0,5202	2,05E-05	8,65E-05	0,5627	0,346	4,19E-07	1,69E-06	0,9549	0,9983	0,0208	0,2117	0,0001	0,0001	0,9695	0,8109	1,0108	1,5614	1,011	100	100	100	100	100
0,743	0,7761	0,0507	0,2569	0,1291	0,0361	0,0466	0,093	0,4103	0,1047	0,0113	0,1738	0,0022	0,1058	0,9019	0,2446	0,6805	0,6793	22,0925	1,1566	0,9711	83	67	100	100	100
0,947	0,789	0,1839	0,334	0,0001	0,0003	0,2321	0,1888	0,0007	0,0005	0,71	0,9895	0,1412	0,4371	0,0093	0,0045	0,9049	0,9041	1,0821	1,3894	1,0312	100	100	100	100	100
0,1067	0,5869	0,5902	0,4926	0,002	0,0024	0,6376	0,3684	2,87E-06	7,57E-06	0,2548	0,6815	0,016	0,206	7,40E-05	0,0001	0,977	0,7902	1,0441	1,5405	0,9236	100	100	100	100	100
0,1872	0,7403	0,7664	0,5305	0,0455	0,0155	0,6727	0,3813	0,0098	0,0041	0,4499	0,8482	0,217	0,4776	0,0291	0,012	0,9905	0,8729	1,0339	1,2457	0,9536	100	100	100	100	100
0,8042	0,7761	0,7025	0,5217	0,0019	0,0024	0,8507	0,4393	6,17E-05	8,99E-05	0,586	0,9456	0,8918	0,6902	0,0002	0,0002	0,948	0,9281	0,9178	1,4189	0,958	100	100	100	100	100
0,9619	0,7903	0,9456	0,5776	0,0193	0,0082	0,9214	0,464	0,0014	0,0009	0,9522	0,9983	0,9834	0,7269	0,0012	0,0008	1,0099	0,908	0,9008	2,3846	0,9738	100	100	100	100	100
0,0064	0,3993	0,6505	0,511	0,1657	0,0431	0,4502	0,3049	0,0002	0,0002	0,5456	0,9027	0,0135	0,206	0,0292	0,012	1,1867	0,5024	1,0179	1,5396	0,9721	100	100	100	100	100
0,3239	0,7686	0,1055	0,29	0,0026	0,0026	0,0258	0,0676	0,0002	0,0002	0,4934	0,8663	0,4167	0,5955	6,45E-06	2,31E-05	1,1683	1,0249	0,9175	1,8696	0,831	100	100	100	100	100
0,4729	0,7696	0,0621	0,2611	0,0186	0,008	0,8615	0,4411	0,0038	0,0018	0,0432	0,3679	0,0056	0,1342	0,0302	0,0123	0,9518	0,8608	1,3132	1,3157	0,9769	100	100	100	100	100
0,6433	0,7761	0,5246	0,4658	0,0012	0,0017	0,0264	0,0676	8,40E-05	0,0001	0,1821	0,6208	0,8003	0,6839	2,20E-06	9,45E-06	1,0236	0,9899	0,9732	1,4808	0,8633	100	100	100	100	100
0,7497	0,7761	0,11	0,29	0,1901	0,0492	0,0249	0,0676	0,2972	0,079	0,002	0,0571	0,1829	0,4633	0,004	0,0022	1,0608	1,1194	1,4388	1,3705	0,7736	100	100	100	100	100
0,1142	0,5869	0,65	0,511	0,0008	0,0012	0,6612	0,378	1,01E-06	3,23E-06	0,9399	0,9978	0,0306	0,2689	0,0002	0,0002	0,9686	0,7756	1,0085	1,7357	1,0341	100	100	100	100	100
0,5234	0,7696	0,7009	0,5217	0,2026	0,0518	0,195	0,1784	0,4307	0,1088	0,1179	0,5056	0,3394	0,5578	0,8833	0,2406	0,937	0,8451	0,9892	0,7475	0,7501	83	67	100	33	67
0,2176	0,7469	0,5165	0,4658	0,0013	0,0018	0,5429	0,3386	0,0003	0,0002	0,2682	0,6931	0,1966	0,4677	0,0004	0,0003	0,9785	0,9029	1,0456	1,441	0,9357	100	100	100	100	100
0,689	0,7761	0,3322	0,3883	0,0006	0,001	0,1363	0,1527	0,0005	0,0004	0,515	0,871	0,5031	0,5974	2,33E-05	4,88E-05	0,9802	0,9571	0,897	1,5509	0,834	100	100	100	100	100
0,8615	0,7761	0,1161	0,2903	6,52E-06	4,78E-05	0,0174	0,058	2,29E-05	4,04E-05	0,0042	0,0875	0,4283	0,5974	0,1648	0,0569	0,7387	0,7688	1,0034	4,9203	3,6573	100	100	100	100	83
0,1135	0,5869	0,5074	0,4658	0,9248	0,1784	0,0899	0,13	0,2199	0,061	0,1781	0,6208	0,5499	0,6089	0,0533	0,0206	1,1113	0,9481	1,043	1,1313	0,8719	100	100	100	100	100
0,438	0,7696	0,4981	0,4637	0,205	0,0521	0,174	0,1676	0,051	0,0166	0,1149	0,5056	0,8725	0,6839	0,002	0,0012	1,1193	0,8546	0,8647	1,4237	0,6983	100	100	100	100	100
0,0738	0,5869	0,3318	0,3883	0,0571	0,0187	0,0602	0,1049	0,0158	0,0061	0,0692	0,4437	0,535	0,6008	0,0008	0,0006	1,0388	0,9064	0,9659	1,1692	0,8156	100	100	100	100	100
0,7155	0,7761	0,1369	0,312	0,1581	0,0424	0,1473	0,1618	0,1021	0,0308	0,8681	0,9936	0,1891	0,4651	0,0041	0,0022	1,0335	1,0002	0,8942	1,1674	0,886	100	100	100	100	100
0,0603	0,5869	0,7427	0,5305	0,255	0,0623	0,8035	0,4282	0,0038	0,0018	0,9429	0,9978	0,0215	0,2117	0,4018	0,1233	0,974	0,7738	1,0133	1,0855	1,0116	100	100	100	100	100
0,1608	0,6897	0,3227	0,3883	0,0039	0,0032	0,7966	0,4266	0,0002	0,0002	0,3093	0,7488	0,0185	0,206	0,0073	0,0037	0,9767	0,7095	1,4006	1,9695	1,0498	83	100	100	100	100
0,546	0,7696	0,5167	0,4658	0,0117	0,0059	0,0449	0,0914	0,0021	0,0012	0,2283	0,6529	0,9081	0,6974	8,72E-05	0,0001	1,0448	0,9847	0,9774	1,4339	0,8493	100	100	100	100	100
0,6818	0,7761	0,7129	0,5217	0,0183	0,008	0,2187	0,1819	0,025	0,009	0,4888	0,8663	0,7701	0,6812	0,0103	0,0049	1,0354	1,0176	0,9883	1,3907	0,8876	100	100	100	100	100
0,0503	0,5869	0,0551	0,2611	0,0845	0,0258	0,1173	0,1437	0,7626	0,18	0,877	0,9936	0,8239	0,6839	0,9402	0,2527	5,9856	1,6832	1,2791	1,7908	2,398	100	67	83	83	83
0,2159	0,7469	0,2402	0,3676	0,4177	0,0957	0,1541	0,162	0,769	0,18	0,4781	0,8663	0,9976	0,7269	0,321	0,1036	1,1631	0,9979	1,0028	1,0507	0,9321	100	100	100	100	100
0,0479	0,5869	0,8722	0,5527	0,2883	0,0685	0,1564	0,162	0,2514	0,0686	0,108	0,5048	0,0488	0,3019	0,4529	0,1362	1,2662	0,8	1,4182	1,0262	0,9133	100	100	100	100	100
0,7966	0,7761	0,7007	0,5217	0,0055	0,0041	0,6272	0,3662	0,001	0,0007	0,8382	0,9936	0,5005	0,5974	0,0076	0,0038	0,9481	0,9097	0,9925	1,3831	1,0283	100	100	100	100	100
0,0143	0,5212	0,5404	0,4737	0,3975	0,092	0,0065	0,0439	0,0126	0,0051	0,0006	0,0268	0,0032	0,1058	0,0024	0,0014	2,7158	0,7406	1,873	1,8719	0,6263	100	100	100	100	100
0,579	0,7696	0,9145	0,5637	0,5542	0,1201	0,1286	0,1502	0,394	0,1015	0,1187	0,5056	0,8632	0,6839	0,3617	0,1121	1,0416	1,0958	1,0779	0,9784	0,7955	100	100	100	83	100
0,0994	0,5869	0,0402	0,2361	0,7883	0,159	0,0082	0,0448	0,0413	0,0141	0,1919	0,6208	0,4161	0,5955	0,0003	0,0003	1,312	0,8978	0,8087	1,1949	0,6678	100	83	67	100	33
0,5136	0,7696	0,0268	0,2265	0,8593	0,1694	0,059	0,1049	0,488	0,1221	0,7864	0,9936	0,0121	0,206	0,0903	0,0335	1,0769	1,1396	0,8493	1,0679	0,8775	100	100	100	100	100
0,0491	0,5869	0,4084	0,41	0,0012	0,0017	0,0434	0,09	0,2559	0,0693	0,219	0,6529	0,0381	0,2767	0,0466	0,0184	1,3958	0,8144	1,1713	0,5962	0,9027	100	67	100	100	100
0,125	0,6095	0,361	0,3975	0,0463	0,0157	0,2489	0,1952	0,2514	0,0686	0,7058	0,9895	0,5242	0,6008	0,1635	0,0569	1,1992	0,9347	1,0516	0,7697	0,9893	100	100	100	100	100
0,1033	0,5869	0,9073	0,5618	0,0259	0,0102	0,5045	0,3265	0,2591	0,0696	0,6603	0,9895	0,1258	0,4123	0,0315	0,0127	1,1915	0,8714	1,1732	0,7062	1,0577	100	100	100	100	100
0,2206	0,7469	0,7117	0,5217	0,1632	0,0427	0,0945	0,13	0,0141	0,0055	0,204	0,6271	0,4895	0,5974	0,0036	0,002	1,0452	0,6774	0,9957	1,6862	0,5795	83	83	83	100	50
0,2774	0,7686	0,0623	0,2611	0,0806	0,0251	0,2021	0,1787	0,0016	0,001	0,4055	0,8222	0,3153	0,5527	0,0011	0,0008	1,2972	0,9558	0,7643	1,8959	0,9035	100	83	50	100	100
0,1546	0,6838	0,1437	0,312	0,5794	0,1237	0,0235	0,0676	0,2566	0,0693	0,9944	1	0,8529	0,6839	0,3353	0,1061	1,2059	0,895	0,9102	1,1425	0,8587	100	100	100	100	100
0,9368	0,7862	0,287	0,3836	0,0113	0,0058	0,3029	0,2309	9,57E-05	0,0001	0,9968	1	0,4532	0,5974	1,69E-05	3,85E-05	1,1703	1,1004	0,9265	4,1073	0,9306	100	100	100	100	100
0,5838	0,7696	0,1677	0,3188	0,0096	0,0053	0,1829	0,1716	0,0001	0,0002	0,9887	1	0,2649	0,5295	1,01E-05	3,06E-05	1,0799	1,1421	0,8566	4,6872	0,8575	100	100	100	100	100
0,7647	0,7761	0,1508	0,312	0,648	0,1364	0,1991	0,1784	0,9583	0,2145	0,9974	1	0,2442	0,4979	0,2636	0,087	1,0889	1,0633	0,9451	1,0488	0,9497	100	100	100	100	100
0,4327	0,7696	0,0347	0,2361	0,0002	0,0004	0,5412	0,3386	2,01E-07	1,03E-06	0,0827	0,4602	0,1406	0,4371	3,60E-07	2,44E-06	1,0362	0,9409	0,7836	2,3854	0,9647	100	100	100	100	100
				0,0615	0,0199			0,0029	0,0015	1	1	1	0,7269	0,0029	0,0017	0,3358	0,3358	0,3358	0,7129	0,3358	0	0	0	83	0
0,5876	0,7696	0,4448	0,4228	0,0036	0,0031	0,5091	0,3268	0,0003	0,0003	0,9434	0,9978	0,8675	0,6839	0,0004	0,0003	0,8849	0,9618	0,9894	2,2837	0,9833	100	100	100	100	100
0,439	0,7696	0,2599	0,3773	0,0066	0,0045	0,134	0,1527	0,0005	0,0004	0,4096	0,8222	0,6625	0,6509	2,64E-05	5,02E-05	1,0504	0,9778	0,9433	1,3915	0,8862	100	100	100	100	100
0,3261	0,7686	0,7444	0,5305	0,0045	0,0035	0,1351	0,1527	3,41E-06	7,57E-06	0,5115	0,871	0,74	0,6783	1,68E-06	7,85E-06	1,0491	0,8602	0,9851	9,2494	0,7774	100	100	100	100	100
0,5419	0,7696	0,6887	0,5217	0,0056	0,0041	0,594	0,3563	4,42E-05	7,27E-05	0,5874	0,9456	0,8531	0,6839	8,41E-06	2,69E-05	0,8928	1,0271	0,9236	9,115	0,7751	100	100	100	100	100
0,6228	0,7696	0,6119	0,4979	0,0023	0,0026	0,3507	0,2533	3,41E-06	7,57E-06	0,4206	0,8268	0,5859	0,6119	6,11E-06	2,31E-05	0,8867	0,9618	0,8106	7,9873	1,044	100	100	100	100	100

0,8897	0,7761	0,9318	0,5717	0,0006	0,001	0,1728	0,1676	1,34E-06	4,03E-06	0,2175	0,6529	0,8319	0,6839	1,32E-05	3,39E-05	0,8007	0,8231	0,7218	10,8935	1,0882	100	100	100	100	100
0,2323	0,7686	0,4101	0,41	0,0202	0,0085	0,0663	0,112	0,1258	0,0369	0,5359	0,8931	0,7623	0,6812	0,2171	0,0733	0,754	1,1334	1,1019	1,931	1,1528	83	83	83	100	100
0,3902	0,7696	0,2648	0,3802	0,0038	0,0032	0,3712	0,2629	5,08E-05	8,10E-05	0,9973	1	0,8218	0,6839	8,51E-05	0,0001	0,7833	0,8787	0,9537	5,0208	0,9899	83	100	100	100	83
0,8724	0,7761	0,2365	0,3676	0,0101	0,0054	0,5886	0,355	0,0003	0,0002	0,6572	0,9895	0,4711	0,5974	0,0005	0,0004	0,8099	0,8243	1,0319	3,9242	0,9158	83	83	83	100	100
0,2211	0,7469	0,0697	0,2611	0,0379	0,0135	0,0925	0,13	0,0003	0,0003	0,8472	0,9936	0,5808	0,6119	0,0001	0,0002	1,0654	0,7699	0,657	4,1401	0,683	83	83	50	83	50
0,2758	0,7686	0,3939	0,4059	0,1294	0,0361	0,9681	0,4726	0,0711	0,0226	0,5043	0,8672	0,1768	0,4547	0,2495	0,0833	1,0201	0,9327	1,125	1,1988	1,0481	100	100	100	100	100
0,5108	0,7696	0,299	0,3881	0,1173	0,0337	0,3337	0,2483	0,3316	0,0863	0,0308	0,2837	0,7262	0,6783	0,0077	0,0038	1,204	1,0447	0,9399	0,8295	1,3926	100	100	100	100	100
0,5366	0,7696	0,3111	0,3883	0,0063	0,0043	0,004	0,0394	0,0011	0,0007	0,0223	0,2565	0,7205	0,6783	0,3485	0,1091	1,1788	1,1199	1,0794	0,7595	0,8315	100	100	100	100	100
0,8671	0,7761	0,5139	0,4658	0,4806	0,1073	0,1498	0,162	0,6635	0,1589	0,0812	0,4602	0,381	0,5611	0,6232	0,1834	0,9896	1,0367	0,9105	1,08	1,1458	100	100	100	100	100
0,6671	0,7761	0,2915	0,3839	0,42	0,0957	0,374	0,2632	0,4022	0,1031	0,0581	0,4308	0,4846	0,5974	0,6629	0,1918	0,9909	1,0091	1,0724	0,9477	0,9253	100	100	100	100	100
0,3364	0,7686	0,2106	0,3554	0,4542	0,103	0,6202	0,3643	0,7767	0,181	0,0983	0,4914	0,6657	0,6509	0,3254	0,1039	0,9787	1,0517	1,1063	1,0157	0,9419	100	100	100	100	100
0,0622	0,5869	0,1139	0,29	0,0944	0,0281	0,6764	0,3813	0,0006	0,0004	0,2351	0,6594	0,5578	0,6119	0,0334	0,0135	1,0403	0,881	0,9162	1,1733	1,005	100	100	100	100	100
0,1046	0,5869	0,0302	0,2265	0,0258	0,0102	0,0117	0,0549	0,0001	0,0001	0,8626	0,9936	0,0699	0,3272	0,0141	0,0063	1,0647	1,1706	0,9521	0,7292	0,9339	100	100	100	100	100
0,7213	0,7761	0,8149	0,5428	0,1046	0,0307	0,2431	0,1934	0,0714	0,0226	0,191	0,6208	0,6005	0,6121	0,2916	0,0958	0,9309	0,949	0,9062	1,2397	1,0572	100	100	100	100	100
0,0196	0,5212	0,2437	0,3676	0,5063	0,1117	0,0285	0,0687	0,0413	0,0141	0,0274	0,2663	0,4966	0,5974	0,001	0,0007	1,209	0,8973	1,0317	1,324	0,7253	100	100	100	100	100
0,1144	0,5869	0,7758	0,5305	0,1252	0,0355	0,245	0,1935	0,0011	0,0007	0,278	0,7104	0,1493	0,4456	0,0027	0,0016	1,0525	0,9088	1,0234	1,1965	0,9388	100	100	100	100	100
0,7229	0,7761	0,3394	0,3883	0,004	0,0032	0,8117	0,4284	6,55E-05	9,03E-05	0,4111	0,8222	0,487	0,5974	8,86E-05	0,0001	0,9835	0,9397	0,8785	1,5396	0,9506	100	100	100	100	100
0,57	0,7696	0,6619	0,5164	0,0487	0,0161	0,1665	0,1676	0,0897	0,0276	0,77	0,9936	0,9709	0,7247	0,0544	0,0209	1,0023	0,9439	0,9759	1,254	0,8712	100	100	100	100	100
0,0908	0,5869	0,4588	0,4331	0,1626	0,0427	0,0687	0,1142	0,0464	0,0156	0,0619	0,4354	0,5355	0,6008	0,0024	0,0014	1,0866	0,961	1,0358	1,2337	0,8427	100	100	100	100	100
0,5716	0,7696	0,7433	0,5305	0,0081	0,0051	0,2051	0,1787	0,0022	0,0012	0,0752	0,4437	0,8866	0,6894	2,09E-05	4,52E-05	0,9777	1,0065	1,0001	1,345	0,8662	100	100	100	100	100
0,8731	0,7761	0,0696	0,2611	0,062	0,0199	0,006	0,0439	0,0504	0,0165	0,7083	0,9895	0,2055	0,4707	0,6981	0,201	1,1444	1,1462	0,9694	0,8982	0,9199	100	100	100	100	100
0,7562	0,7761	0,5953	0,493	0,2657	0,0642	0,0999	0,1302	0,5196	0,1287	0,1341	0,5389	0,7679	0,6812	0,0205	0,0088	1,0061	0,9895	0,9681	1,075	0,7931	100	100	100	100	100
0,3051	0,7686	0,0645	0,2611	0,0891	0,0268	0,0261	0,0676	0,4129	0,1048	0,435	0,8268	0,369	0,5611	0,3896	0,1201	1,1963	1,0655	0,9383	0,9544	0,8462	100	100	100	100	100
0,8333	0,7761	0,8523	0,5501	0,667	0,1397	0,3424	0,2506	0,873	0,1989	0,6073	0,954	0,9135	0,6974	0,6433	0,187	1,0301	1,0004	1,0465	0,9793	0,9176	100	100	100	100	100
0,7495	0,7761	0,4182	0,4121	0,0005	0,001	0,1334	0,1527	4,31E-07	1,69E-06	0,4008	0,8222	0,6164	0,6209	3,08E-08	4,30E-07	1,0859	0,9749	0,8786	4,1839	0,7511	100	100	100	100	100
0,8674	0,7761	0,5223	0,4658	0,0093	0,0053	0,7406	0,411	0,002	0,0011	0,2299	0,6529	0,3438	0,5578	0,0036	0,002	1,167	1,0765	1,2926	0,5916	1,0722	100	100	100	100	100
0,2987	0,7686	0,0177	0,2265	0,4761	0,1068	0,6774	0,3813	0,8773	0,199	0,0212	0,2565	0,0603	0,3272	0,7274	0,2065	1,0977	1,0129	0,8561	1,033	1,062	100	100	100	100	100
0,8922	0,7761	0,8639	0,5501	0,7358	0,1505	0,2049	0,1787	0,768	0,18	0,1697	0,6208	0,7479	0,6795	0,4332	0,1309	0,9565	0,9671	0,9299	1,0172	1,0859	100	100	100	83	100
0,4838	0,7696	0,5757	0,4857	0,004	0,0032	0,3831	0,2679	0,0002	0,0002	0,6687	0,9895	0,8199	0,6839	0,0004	0,0003	0,8623	0,9793	0,8849	3,6168	1,0124	100	100	100	100	100
0,4796	0,7696	0,8571	0,5501	0,2211	0,0552	0,0398	0,084	0,1083	0,0323	0,1626	0,6201	0,7552	0,6812	0,0107	0,005	1,0624	1,01	1,062	1,226	0,8917	100	100	100	100	100
0,4246	0,7696	0,566	0,4855	0,572	0,1232	0,7652	0,4187	0,9215	0,2081	0,3703	0,7946	0,192	0,4651	0,7419	0,2096	0,9651	1,0396	0,9066	1,0378	0,9837	100	100	100	100	100
0,5913	0,7696	0,0295	0,2265	0,0032	0,0029	0,1724	0,1676	2,17E-05	3,95E-05	0,3369	0,7597	0,2193	0,4776	1,15E-05	3,32E-05	1,0494	1,0184	0,9218	1,5575	0,9904	100	100	100	100	100
0,1633	0,6897	0,3113	0,3883	0,8703	0,1709	0,1284	0,1502	0,0292	0,0102	0,6306	0,9734	0,0027	0,1058	0,1359	0,0483	1,1324	1,547	0,9352	1,0712	0,8606	100	100	100	100	100
0,78	0,7761	0,4085	0,41	0,0139	0,0066	0,8639	0,4411	0,038	0,0131	0,3658	0,7946	0,6056	0,6136	0,0163	0,0072	1,053	1,0356	0,9788	0,8159	1,0635	100	100	100	100	100
0,5977	0,7696	0,7944	0,5362	0,0052	0,0039	0,3348	0,2483	0,058	0,0186	0,2288	0,6529	0,5081	0,5974	0,1645	0,0569	0,7939	0,9605	0,7578	1,4153	1,1055	50	67	67	100	50
0,296	0,7686	0,715	0,5217	0,8107	0,1628	0,6118	0,3642	0,5653	0,1367	0,9962	1	0,5966	0,6119	0,967	0,2577	0,9845	1,0579	1,0136	1,0149	1,0092	100	100	100	100	100
0,3236	0,7686	0,4144	0,4113	0,2846	0,068	0,4527	0,3049	0,8264	0,1915	0,0464	0,3701	0,8754	0,6839	0,021	0,009	0,9208	1,081	1,0681	1,1321	0,7529	50	100	100	100	83
0,8168	0,7761	0,0036	0,1608	5,20E-05	0,0002	0,0015	0,0322	2,02E-05	3,81E-05	0,0546	0,4188	0,004	0,1113	0,8083	0,2242	0,7382	0,7246	1,0439	1,3531	1,3389	100	100	100	100	100
0,642	0,7761	0,1675	0,3188	0,0092	0,0053	0,6872	0,3849	0,0001	0,0001	0,3332	0,7597	0,2883	0,5355	9,83E-05	0,0001	0,9511	0,9942	0,8488	2,0654	0,973	100	100	100	100	100
0,4502	0,7696	0,2698	0,3832	0,0029	0,0027	0,2384	0,191	0,0501	0,0165	0,8438	0,9936	0,0933	0,3664	0,0006	0,0005	0,986	1,0704	0,8707	1,3709	0,8531	100	100	100	100	100
0,5492	0,7696	0,3673	0,3975	0,0152	0,0069	0,6594	0,378	0,1249	0,0368	0,2818	0,7121	0,1762	0,4547	0,0723	0,0273	0,9545	1,021	0,8957	1,1685	0,985	100	100	100	100	100
0,0882	0,5869	0,158	0,3183	0,0081	0,0051	0,5056	0,3265	5,25E-05	8,12E-05	0,8096	0,9936	0,0356	0,2705	0,0055	0,003	1,0665	1,2486	0,9346	0,6688	0,991	100	100	100	100	100
0,0731	0,5869	0,7071	0,5217	0,1358	0,0376	0,0635	0,109	0,0021	0,0012	0,0904	0,4805	0,0551	0,3272	0,7777	0,2177	1,0237	0,8078	1,0739	1,3323	1,3662	100	100	100	100	100
0,8556	0,7761	0,5748	0,4857	0,2772	0,0666	0,1194	0,1437	0,2005	0,0568	0,1178	0,5056	0,2813	0,5355	0,0006	0,0005	0,9789	0,9342	1,0339	0,8124	1,2131	67	100	100	67	100
0,5206	0,7696	0,2261	0,3676	0,0026	0,0026	0,5822	0,3536	0,0001	0,0001	0,4279	0,8268	0,0916	0,3664	1,45E-05	3,51E-05	0,9892	1,0351	0,8941	1,5655	0,9508	100	100	100	100	100
0,6254	0,7696	0,7623	0,5305	5,50E-07	1,21E-05	0,0029	0,0382	2,88E-05	4,90E-05	0,0047	0,0905	0,4443	0,5974	0,0074	0,0037	0,9302	0,9726	0,9175	1,446	1,1584	100	100	100	100	100
0,2914	0,7686	0,7782	0,5305	0,0015	0,0019	0,0148	0,0558	3,84E-06	7,83E-06	0,094	0,4805	0,4396	0,5974	3,29E-05	5,56E-05	0,8927	0,9685	0,8747	2,6148	1,1165	100	100	100	100	100
0,0185	0,5212	0,6162	0,4979	2,56E-05	8,65E-05	0,3239	0,2435	2,39E-08	1,52E-07	0,2045	0,6271	0,1113	0,3876	1,26E-08	3,59E-07	0,8574	1,1096	0,8191	8,4448	1,0984	100	100	100	100	100
0,893	0,7761	0,2362	0,3676	0,0432	0,0151	0,5214	0,3308	0,0026	0,0014	0,4348	0,8268	0,0766	0,3369	0,0002	0,0002	1,0623	1,0511	0,8548	1,5583	0,9333	100	100	100	100	100
0,4496	0,7696	0,0176	0,2265	0,0048	0,0037	0,0867	0,13	0,0008	0,0006	0,8271	0,9936	0,0031	0,1058	0,4263	0,1295	1,2063	1,3027	0,9117	0,8538	0,9513	100	100	100	100	100
0,9394	0,7862	0,014	0,2265	0,0322	0,0122	0,1717	0,1676	0,0223	0,0081	0,3435	0,7597	0,0346	0,2705	0,0012	0,0008	1,0499	1,0477	0,8152	1,3975	0,9148	100	100	100	100	100
0,1455	0,6759	0,0093	0,2265	0,7636	0,1555	0,0188	0,0607	0,1168	0,0347	0,202	0,6271	0,4717	0,5974	0,0014	0,001	1,073	0,9776	0,9265	1,0893	0,856	100	100	100	100	100
0,3129	0,7686	0,1942	0,334	0,0034	0,003	0,0043	0,0394	0,0001	0,0001	0,0747	0,4437	0,7592	0,6812	9,79E-07	5,40E-06	1,0548	0,9715	0,9491	1,3595	0,8259	100	100	100	100	100
0,2016	0,7469	0,104	0,29	0,9777	0,1877	0,0291	0,0687	0,0475	0,0157	0,329	0,7597	0,5924	0,6119	0,0016	0,001	1,1292	0,999	0,9678	1,1166	0,9117	100	100	100	100	100
0,4862	0,7696	0,0448	0,2522	0,0443	0,0152	0,016	0,0558	0,0019	0,0011	0,721	0,9927	0,347	0,5578	8,55E-05	0,0001	1,0846	1,0141	0,8906	1,6788	0,8494	100	100	100	100	100
0,1933	0,7469	0,7587	0,5305	0,0372	0,0134	0,8235	0,4305	0,131	0,0382	0,9251	0,9978	0,2312	0,4892	0,0088	0,0043	0,9392	1,0595	0,9592	1,2134	0,953	100	100	100	100	100
0,4078	0,7696	0,9991	0,5915	0,0142	0,0066	0,7579	0,4181	0,0177	0,0066	0,8096	0,9936	0,4842	0,5974	0,0063	0,0033	0,911	1,0216	0,9167	1,5444	0,9475	100	100	100	100	100
0,5456	0,7696	0,1776	0,3284	0,0612	0,0199	0,1889	0,1758	0,0039	0,0018	0,827	0,9936	0,5109	0,5974	0,0005	0,0004	1,0725	1,0225	0,9693	1,3294	0,9582	100	100	100	100	100
0,1727	0,713	0,0833	0,2611	0,0915	0,0274	0,012	0,0549	0,8353	0,192	0,3976	0,8222	0,3757	0,5611	0,1345	0,0483	1,1222	1,0181	0,9565	1,0003	0,8928	100	100	100	100	100
0,8341	0,7761	0,1126	0,29	0,0143	0,0066	0,1119	0,1396	0,0035	0,0017	0,9094	0,9978	0,1716	0,4547	0,0002	0,0002	1,0288	1,0139	0,9024	1,3445	0,9092	100	100	100	100	100

0,5108	0,7696	0,0053	0,1785	0,1573	0,0424	0,0071	0,0439	0,0733	0,0231	0,1876	0,6208	0,0823	0,3526	5,67E-05	8,61E-05	1,0626	1,0021	0,7862	1,2988	0,6752	100	100	100	100	100
0,4773	0,7696	0,8543	0,5501	0,0025	0,0026	0,6304	0,3662	0,0013	0,0008	0,8005	0,9936	0,5083	0,5974	0,0001	0,0002	0,9188	0,9943	0,9148	1,725	0,88	100	100	100	100	100
0,3516	0,7686	0,6014	0,495	0,0095	0,0053	0,9523	0,4726	5,88E-05	8,82E-05	0,7141	0,9895	0,2713	0,5296	0,0003	0,0003	0,9496	0,8861	0,9888	1,476	0,9509	100	100	100	100	100
0,9274	0,7833	0,1744	0,327	0,1057	0,0308	0,9988	0,4809	0,2297	0,0634	0,1821	0,6208	0,2014	0,4677	0,2527	0,0839	1,0125	1,0069	0,8673	1,1774	1,0357	100	100	83	100	100
0,693	0,7761	0,0005	0,067	0,0136	0,0065	0,0968	0,13	0,0232	0,0084	0,1773	0,6208	0,014	0,206	0,0013	0,0008	1,0616	1,0392	0,824	1,2631	0,9316	100	100	100	100	100
0,3843	0,7696	0,0857	0,2611	0,0017	0,0022	0,9687	0,4726	0,0002	0,0002	0,2681	0,6931	0,0705	0,3272	2,83E-05	5,13E-05	0,9791	1,0566	0,8737	1,6684	0,9804	100	100	100	100	100
0,3084	0,7686	0,5912	0,4926	0,0153	0,0069	0,2374	0,191	0,0778	0,0241	0,8217	0,9936	0,1629	0,4547	0,0021	0,0013	0,9313	1,0817	0,8866	1,4017	0,8418	100	100	100	100	100
0,7584	0,7761	0,2504	0,3676	0,0128	0,0064	0,1822	0,1716	0,0007	0,0005	0,8684	0,9936	0,158	0,4547	1,71E-05	3,85E-05	1,0425	1,0695	0,8851	1,8463	0,8643	100	100	100	100	100
0,5634	0,7696	0,8162	0,5428	0,0002	0,0005	0,1518	0,162	3,58E-06	7,60E-06	0,3041	0,744	0,854	0,6839	2,43E-05	4,92E-05	0,9597	0,9833	0,9745	1,4588	1,0394	100	100	100	100	100
0,9629	0,7903	0,1353	0,312	0,0352	0,0129	0,1385	0,1536	0,0086	0,0037	0,8243	0,9936	0,1379	0,4371	0,0001	0,0002	1,0762	1,0607	0,8945	1,4756	0,8813	100	100	100	100	100
0,2706	0,7686	0,1497	0,312	0,0079	0,0051	0,3658	0,2608	0,0002	0,0002	0,6926	0,9895	0,9504	0,7164	0,0004	0,0003	1,0309	0,9267	0,9128	1,4963	0,954	100	100	100	100	100
0,0299	0,5869	0,0886	0,2611	0,7124	0,1478	0,0157	0,0558	0,0467	0,0156	0,0721	0,4437	0,7214	0,6783	0,0016	0,001	1,0797	0,9354	0,964	1,116	0,835	100	100	100	100	100
0,8326	0,7761	0,4355	0,4169	0,0033	0,0029	0,3419	0,2506	0,0009	0,0006	0,9275	0,9978	0,6516	0,6446	0,0002	0,0002	0,9954	0,9849	0,9449	1,3256	0,9368	100	100	100	100	100
0,0407	0,5869	0,3394	0,3883	0,5311	0,1156	0,4802	0,3176	0,006	0,0027	0,6029	0,954	0,2378	0,4908	0,1991	0,068	1,0391	0,8901	0,966	1,0878	0,9934	100	100	100	100	100
0,9609	0,7903	0,0514	0,2569	0,6175	0,1312	0,0541	0,1023	0,5459	0,1327	0,4228	0,8268	0,2354	0,4908	0,0152	0,0068	1,1329	1,1223	0,8921	1,3598	0,792	100	100	100	100	100
0,2132	0,7469	0,1499	0,312	0,0134	0,0065	0,033	0,0739	0,0001	0,0001	0,3676	0,7946	0,8204	0,6839	7,57E-06	2,55E-05	1,047	0,9469	0,9306	1,3779	0,8679	100	100	100	100	100
0,3474	0,7686	0,0703	0,2611	0,3034	0,0714	0,0397	0,084	0,0172	0,0065	0,7061	0,9895	0,3826	0,5611	0,0009	0,0007	1,2311	1,0692	0,9496	1,3905	0,9108	100	100	100	100	100
0,8538	0,7761	0,1526	0,312	0,0131	0,0065	0,2272	0,1875	0,0008	0,0006	0,8446	0,9936	0,3013	0,5527	0,0001	0,0001	1,0165	1,0016	0,9003	1,5245	0,9182	100	100	100	100	100
0,856	0,7761	0,1461	0,312	0,0425	0,015	0,6138	0,3642	0,0223	0,0081	0,1943	0,6208	0,1048	0,3729	0,0103	0,0049	0,9834	0,9608	0,8212	1,2129	0,9285	100	100	100	100	100
0,6103	0,7696	0,0802	0,2611	0,0237	0,0095	0,0265	0,0676	0,0005	0,0004	0,7878	0,9936	0,3614	0,5611	2,87E-05	5,13E-05	1,1074	1,0551	0,928	1,9788	0,8906	100	100	100	100	100
0,899	0,777	0,3711	0,3975	0,0088	0,0053	0,156	0,162	0,0006	0,0005	0,8177	0,9936	0,4421	0,5974	6,03E-05	8,94E-05	1,0137	1,0028	0,939	1,4897	0,9119	100	100	100	100	100
0,1843	0,7403	0,8606	0,5501	0,0023	0,0026	0,211	0,1795	0,0013	0,0008	0,2468	0,6677	0,209	0,4722	0,001	0,0007	0,9019	1,0021	0,9071	1,3323	0,9944	100	100	100	100	100
0,791	0,7761	0,9804	0,5855	0,0435	0,0151	0,7953	0,4266	0,0165	0,0063	0,6919	0,9895	0,7425	0,6783	0,0032	0,0018	0,9377	0,9597	0,9156	1,2626	0,8904	100	100	100	100	100
0,5369	0,7696	0,0224	0,2265	0,0818	0,0253	0,0103	0,0516	0,0286	0,0101	0,9239	0,9978	0,0619	0,3272	0,0003	0,0002	1,065	1,0221	0,8289	1,3466	0,8166	100	100	100	100	100
0,9038	0,7775	0,241	0,3676	0,0024	0,0026	0,5158	0,3292	0,0036	0,0017	0,5052	0,8672	0,1716	0,4547	0,0007	0,0005	1,0056	1,02	0,9251	1,2615	0,9651	100	100	100	100	100
0,498	0,7696	0,0501	0,2569	0,1021	0,0301	0,0139	0,0558	0,0153	0,0059	0,4755	0,8663	0,1919	0,4651	0,0001	0,0002	1,1213	1,036	0,8768	1,4537	0,8034	100	100	100	100	100
0,4155	0,7696	0,1492	0,312	0,0057	0,0041	0,1538	0,162	0,0002	0,0002	0,9956	1	0,4581	0,5974	3,28E-05	5,56E-05	1,0459	0,9861	0,9354	1,3696	0,936	100	100	100	100	100
0,1004	0,5869	0,0293	0,2265	0,0846	0,0258	0,583	0,3536	6,90E-05	9,26E-05	0,0213	0,2565	0,3233	0,5527	0,0022	0,0013	1,1753	0,8858	0,7884	1,6804	1,059	100	100	100	100	100
0,529	0,7696	0,0809	0,2611	0,0179	0,0079	0,0965	0,13	0,0031	0,0015	0,9458	0,9978	0,3268	0,5527	0,0003	0,0002	1,0403	0,9854	0,8931	1,3414	0,8903	100	100	100	100	100
0,0928	0,5869	0,0378	0,2361	0,0082	0,0051	0,0869	0,13	2,98E-06	7,57E-06	0,7597	0,9936	0,6758	0,6569	2,33E-06	9,45E-06	1,0917	0,968	0,9419	1,4224	0,9621	100	100	100	100	100
0,4299	0,7696	0,1218	0,2989	0,0482	0,0161	0,0971	0,13	0,002	0,0012	0,8892	0,9976	0,5043	0,5974	0,0003	0,0003	1,0734	0,9703	0,8818	1,6069	0,8664	100	100	100	100	100
0,4862	0,7696	0,0448	0,2522	0,0443	0,0152	0,016	0,0558	0,0019	0,0011	0,721	0,9927	0,347	0,5578	8,55E-05	0,0001	1,0846	1,0141	0,8906	1,6788	0,8494	100	100	100	100	100
0,9088	0,7781	0,0357	0,2361	0,8786	0,1715	0,0907	0,13	0,8295	0,1915	0,2272	0,6529	0,0183	0,206	0,1388	0,049	1,0663	1,0609	0,8547	1,0822	0,9438	100	100	100	100	100
0,8337	0,7761	0,3695	0,3975	0,0173	0,0078	0,1697	0,1676	0,0027	0,0014	0,5879	0,9456	0,5911	0,6119	0,0002	0,0002	1,0177	1,0049	0,9587	1,3654	0,9165	100	100	100	100	100
0,0808	0,5869	0,0137	0,2265	0,2185	0,0549	0,0205	0,0625	0,0134	0,0054	0,1823	0,6208	0,0979	0,3718	0,0061	0,0032	1,1402	0,9993	0,8343	1,3922	0,9586	100	100	100	100	100
0,5224	0,7696	0,0301	0,2265	0,0364	0,0132	0,053	0,1021	0,0086	0,0037	0,8563	0,9936	0,1489	0,4456	0,0004	0,0003	1,0667	1,0086	0,8545	1,3926	0,8733	100	100	100	100	100
0,4923	0,7696	0,3465	0,3904	0,0091	0,0053	0,8825	0,4464	0,0141	0,0055	0,3405	0,7597	0,1675	0,4547	0,0051	0,0028	0,9455	1,0165	0,863	1,3996	0,9652	100	100	100	100	100
0,7486	0,7761	0,9538	0,5779	0,0099	0,0054	0,2762	0,2135	0,0012	0,0008	0,3263	0,7597	0,845	0,6839	7,33E-05	0,0001	0,9781	1,0034	0,9824	1,5979	0,8763	100	100	100	100	100
0,6247	0,7696	0,017	0,2265	0,1403	0,0386	0,0592	0,1049	0,3076	0,0813	0,7294	0,9927	0,0455	0,2929	0,0021	0,0013	1,0212	1,0698	0,8397	1,2114	0,8208	100	100	100	100	100
0,6886	0,7761	0,0844	0,2611	0,0486	0,0161	0,0929	0,13	0,0066	0,0029	0,7535	0,9936	0,1222	0,4123	0,0003	0,0003	1,0667	1,0214	0,8781	1,3563	0,8984	100	100	100	100	100
0,8538	0,7761	0,1526	0,312	0,0131	0,0065	0,2272	0,1875	0,0008	0,0006	0,8446	0,9936	0,3013	0,5527	0,0001	0,0001	1,0165	1,0016	0,9003	1,5245	0,9182	100	100	100	100	100
0,8817	0,7761	0,319	0,3883	0,0223	0,0092	0,9432	0,4706	0,0138	0,0055	0,4277	0,8268	0,3166	0,5527	0,0221	0,0093	1,0948	1,0986	0,9631	0,7773	1,1099	100	100	100	100	100
0,3503	0,7686	0,0572	0,2611	0,8815	0,1715	0,1193	0,1437	0,5418	0,1323	0,6414	0,9835	0,3248	0,5527	0,0483	0,0189	1,0442	0,9825	0,8775	1,0723	0,8491	100	100	100	100	100
0,2513	0,7686	0,3155	0,3883	0,1098	0,0318	0,2178	0,1819	0,0087	0,0037	0,91	0,9978	0,8272	0,6839	0,011	0,0052	1,0836	0,9529	0,972	1,2644	0,9562	100	100	100	100	100
0,0584	0,5869	0,3809	0,4048	0,7235	0,1494	0,1205	0,1437	0,0906	0,0277	0,1087	0,5048	0,3785	0,5611	0,0188	0,0082	1,1954	0,9085	1,0615	1,2372	0,8586	100	100	100	100	100
								1	0,2219	1	1	1	0,7269	1	0,2641	1	1	1	1	1	0	0	17	0	0
0,1169	0,5869	0,1881	0,334	0,2613	0,0635	0,2967	0,2278	0,5314	0,131	0,8732	0,9936	0,6422	0,6391	0,9973	0,2641	1,3003	0,8863	0,9613	1,0063	1,0279	100	83	83	83	100
0,098	0,5869	0,6722	0,5202	0,5742	0,1232	0,7667	0,4187	0,0129	0,0052	0,3412	0,7597	0,1673	0,4547	0,7497	0,2108	1,1242	0,8583	1,0502	1,1948	1,1488	100	100	100	100	100
0,4637	0,7696	0,5309	0,4684	0,77	0,156	0,348	0,253	0,1917	0,0547	0,6792	0,9895	0,9491	0,7164	0,1034	0,038	1,0262	0,6817	0,7453	1,0165	0,6492	50	67	50	83	50
0,926	0,7833	0,2159	0,3597	0,2531	0,0622	0,1795	0,1714	0,0769	0,0239	0,8286	0,9936	0,1743	0,4547	0,8147	0,225	1,2094	1,1707	0,942	0,9488	0,9181	100	100	100	100	100
0,5783	0,7696	0,1858	0,334	0,0101	0,0054	0,1008	0,1302	0,0009	0,0006	0,3747	0,7946	0,5407	0,6027	2,57E-05	5,02E-05	1,0453	0,9949	0,9315	1,5132	0,8604	100	100	100	100	100
0,9655	0,7903	0,1463	0,312	0,8452	0,1682	0,5642	0,346	0,9284	0,2087	0,8149	0,9936	0,3406	0,5578	0,4164	0,1271	1,0575	1,0871	0,8507	1,1156	1,1384	100	100	100	100	100
0,3881	0,7696	0,3471	0,3904	0,0288	0,0112	0,0585	0,1049	0,0028	0,0015	0,1097	0,5048	0,9887	0,7269	5,51E-05	8,58E-05	1,1116	1,0111	1,0051	1,6776	0,8186	100	100	100	100	100
0,4717	0,7696	0,2434	0,3676	0,0388	0,0138	0,9915	0,4795	0,2718	0,0726	0,4913	0,8663	0,287	0,5355	0,1434	0,0504	0,9924	1,0883	0,8239	1,5089	1,3725	100	100	100	100	100
0,5538	0,7696	0,3933	0,4059	0,0062	0,0043	0,1987	0,1784	0,0086	0,0037	0,4831	0,8663	0,7321	0,6783	0,0791	0,0295	1,2412	1,1423	1,09	0,6356	0,9711	100	100	100	100	100
0,315	0,7686	0,6375	0,5092	0,9228	0,1784	0,8734	0,4439	0,2037	0,0571	0,6587	0,9895	0,4542	0,5974	0,9194	0,2482	1,3158	0,8521	1,0325	1,1245	1,1455	100	100	100	100	100
0,6024	0,7696	0,6184	0,4979	0,344	0,08	0,9634	0,4726	0,018	0,0067	0,38	0,7946	0,9982	0,7269	0,108	0,0393	1,1063	1,0313	1,0715	0,6095	0,8582	100	100	100	100	100
0,534	0,7696	0,435	0,4169	0,5101	0,1117	0,3172	0,2401	0,9898	0,2206	0,7279	0,9927	0,8539	0,6839	0,587	0,1739	1,1189	0,9769	0,9483	0,9698	0,9112	100	100	100	100	100
0,4195	0,7696	0,5683	0,4855	0,6326	0,1337	0,5026	0,3265	0,7056	0,1682	0,866	0,9936	0,7747	0,6817	0,7945	0,2214	0,8981	0,7293	0,7692	0,7867	0,7622	83	50	67	83	50
0,0389	0,5869	0,851	0,5501	0,4055	0,0934	0,5277	0,3329	0,1447	0,042	0,6117	0,954	0,0264	0,2456	0,7203	0,2064	1,611	0,442	1,7781	1,8826	1,5906	100	100	100	100	100

Super Pathway	Sub Pathway	Chemical ID	CAS	RI	Mass
Amino acid	Serine family (phosphoglycerate derived)	100000096	463-003;463-00-3;	1085	146,1
Amino acid	Serine family (phosphoglycerate derived)	100000055	1113-60-6;3369-79-7;	1425,1	221
Amino acid	Serine family (phosphoglycerate derived)	800	52-90-4;56-89-3;	1560,1	218
Amino acid	Serine family (phosphoglycerate derived)	340	56-40-6;	1166	101,9
Amino acid	Serine family (phosphoglycerate derived)	100001851	97-14-3;	1012	148
Amino acid	Serine family (phosphoglycerate derived)	100000286	66638-22-0;	1423	174
Amino acid	Serine family (phosphoglycerate derived)	503	56-45-1;	1389,1	204
Amino acid	Aromatic amino acid metabolism (PEP derived)	460	63-91-2;	2056	166,1
Amino acid	Aromatic amino acid metabolism (PEP derived)	100000293	138-59-0;	1758,1	204
Amino acid	Aromatic amino acid metabolism (PEP derived)	565	73-22-3;	2445	205,1
Amino acid	Aromatic amino acid metabolism (PEP derived)	815	60-18-4;	1516	182,1
Amino acid	Aspartate family (OAA derived)	811	56-41-7;	1147,6	115,9
Amino acid	Aspartate family (OAA derived)	917	70-47-3;	1617,5	188
Amino acid	Aspartate family (OAA derived)	234	56-84-8;	1529,7	232
Amino acid	Aspartate family (OAA derived)	244	56-41-7;107-95-9;	1451,8	174
Amino acid	Aspartate family (OAA derived)	100001645	6232-19-5;	1402	140,9
Amino acid	Aspartate family (OAA derived)	100000458	672-15-1;	1471	218,1
Amino acid	Aspartate family (OAA derived)	407	56-87-1;	1836,7	317,2
Amino acid	Aspartate family (OAA derived)	415	63-68-3;	1530,7	176
Amino acid	Aspartate family (OAA derived)	1083	65-82-7;	1805	190,1
Amino acid	Aspartate family (OAA derived)	1025	4043-87-2;	1396,1	156
Amino acid	Aspartate family (OAA derived)	197	979-92-0;	1480	193,2
Amino acid	Aspartate family (OAA derived)	564	72-19-5;	1412,3	218,1
Amino acid	Glutamate family (alpha-ketoglutarate derived)	1128	1492-24-6;	1215,7	130
Amino acid	Glutamate family (alpha-ketoglutarate derived)	100001081	616-45-5 ;	1190,9	142
Amino acid	Glutamate family (alpha-ketoglutarate derived)	1113	3025-96-5;	1510,2	158
Amino acid	Glutamate family (alpha-ketoglutarate derived)	100001507	502-85-2;	1277	233,1
Amino acid	Glutamate family (alpha-ketoglutarate derived)	231	1119-34-2;	650	175,2
Amino acid	Glutamate family (alpha-ketoglutarate derived)	391	372-75-8;	715	176,1
Amino acid	Glutamate family (alpha-ketoglutarate derived)	141	56-12-2;	1539,7	304,1
Amino acid	Glutamate family (alpha-ketoglutarate derived)	561	56-86-0;	1611,9	246
Amino acid	Glutamate family (alpha-ketoglutarate derived)	563	56-85-9;	684	147,2
Amino acid	Glutamate family (alpha-ketoglutarate derived)	355	5934-29-2;	757	154,1
Amino acid	Glutamate family (alpha-ketoglutarate derived)	100001253	2490-97-3;	1204	189,1
Amino acid	Glutamate family (alpha-ketoglutarate derived)	100000285	6205-08-9;	875	175,2
Amino acid	Glutamate family (alpha-ketoglutarate derived)	100001334	1074-79-9;	2184	158,1
Amino acid	Glutamate family (alpha-ketoglutarate derived)	192	18233-70-0;	895	131,1
Amino acid	Glutamate family (alpha-ketoglutarate derived)	444	3184-13-2;	1763,8	141,9
Amino acid	Glutamate family (alpha-ketoglutarate derived)	480	147-85-3;	796	116,1
Amino acid	Glutamate family (alpha-ketoglutarate derived)	1001	51-35-4;	1537	140
Amino acid	Glutamate family (alpha-ketoglutarate derived)	100002537	1187-99-1;	1658	333,1
Amino acid	Branched Chain Amino Acids (OAA derived)	376	73-32-5;	1614	132,1
Amino acid	Branched Chain Amino Acids (pyruvate derived)	397	61-90-5;	1674	132,2
Amino acid	Branched Chain Amino Acids (pyruvate derived)	566	72-18-4;	1040	118,1
Amino acid	Amines and polyamines	212	2457-80-9;	2427	298,1
Amino acid	Amines and polyamines	100000103	2482-00-0;	1526	174
Amino acid	Amines and polyamines	49	110-60-1;	1705,8	174
Amino acid	Amines and polyamines	50	124-20-9;	1998,9	144
Amino acid	Glutathione metabolism	1021	98-79-3;	1446	130,1
Amino acid	Glutathione metabolism	100001437	13081-14-6;	821	427,1
Amino acid	Glutathione metabolism	448	103239-24-3;	1535	307,3
Carbohydrate	Glycolysis	100000143	96-26-4;62147-49-3;	1349	306,1

Carbohydrate	Glycolysis	572	50-99-7;	1866,8	217,1
Carbohydrate	Glycolysis	291	103192-55-8;	2042,7	387,2
Carbohydrate	Glycolysis	1052	600-19-1;	1360,7	189
Carbohydrate	Glycolysis	823	127-17-3;	1130,6	217
Carbohydrate	TCA cycle	93	305-72-6;328-50-7;22202-68-2;	1779	419,1
Carbohydrate	TCA cycle	1124	77-92-9;	1763,4	273,1
Carbohydrate	TCA cycle	330	100-17-8;	1382,1	245
Carbohydrate	TCA cycle	409	6915-15-7;	1502	233
Carbohydrate	TCA cycle	252	110-15-6;	1348	247
Carbohydrate	Calvin cycle and pentose phosphate	19	18265-46-8;108321-05-7;	1928,4	315,1
Carbohydrate	Calvin cycle and pentose phosphate	100001628	2646-35-7;	2070	691,35
Carbohydrate	Amino sugar and nucleotide sugar	828	28697-53-2;	1631,6	217
Carbohydrate	Amino sugar and nucleotide sugar	100000012	7643-75-6;	1687,5	307,1
Carbohydrate	Amino sugar and nucleotide sugar	100001937	7643-75-7;	1736	292,1
Carbohydrate	Amino sugar and nucleotide sugar	100000846	149-32-6;	1517,5	217
Carbohydrate	Amino sugar and nucleotide sugar	100001320	88759-55-1;	1546,9	292,1
Carbohydrate	Amino sugar and nucleotide sugar	918	2438-80-4;	1682,2	204
Carbohydrate	Amino sugar and nucleotide sugar	338	527-07-1;	1879,4	333
Carbohydrate	Amino sugar and nucleotide sugar	289	66-84-2;	1833	203
Carbohydrate	Amino sugar and nucleotide sugar	100000281	7512-17-6;	1903,3	245
Carbohydrate	Amino sugar and nucleotide sugar	100000406	488-81-3;	1692,4	217
Carbohydrate	Amino sugar and nucleotide sugar	914	50-69-1;	1652	204
Carbohydrate	Amino sugar and nucleotide sugar	100000147	488-84-6;	1662	306,1
Carbohydrate	Amino sugar and nucleotide sugar	100001419	2418-52-2;	1513	217,1
Carbohydrate	Amino sugar and nucleotide sugar	1096	117756-22-6;	1986	243,1
Carbohydrate	Amino sugar and nucleotide sugar	100001627	73686-31-7;	1722	292
Carbohydrate	Amino sugar and nucleotide sugar	826	609-06-3;	1723,9	204
Carbohydrate	Amino sugar and nucleotide sugar	100000421	551-84-8;	1668	306,1
Carbohydrate	Inositol metabolism	370	106032-59-1;	2057,8	318,1
Carbohydrate	Inositol metabolism	100001004		2072,9	318,1
Carbohydrate	Inositol metabolism	363	87-89-8;	1924,9	217
Carbohydrate	Sucrose, glucose, fructose metabolism	100000095	103404-70-2;	1942	373,2
Carbohydrate	Sucrose, glucose, fructose metabolism	878	57-48-7;	1758	217
Carbohydrate	Sucrose, glucose, fructose metabolism	100000908	16908-86-4;	2226,1	433,3
Carbohydrate	Sucrose, glucose, fructose metabolism	287	59-23-4;	1793,8	203,9
Carbohydrate	Sucrose, glucose, fructose metabolism	100000873	91510-62-2;	1890	204
Carbohydrate	Sucrose, glucose, fructose metabolism	100000277	69-65-8;	1839	319,1
Carbohydrate	Sucrose, glucose, fructose metabolism	294	70442-25-0;104872-94-8;	2004,6	387,1
Carbohydrate	Sucrose, glucose, fructose metabolism	879	17629-30-0;	997	503,2
Carbohydrate	Sucrose, glucose, fructose metabolism	920	10030-85-0;	1669,4	204
Carbohydrate	Sucrose, glucose, fructose metabolism	935	57-50-1;	875	341,2
Carbohydrate	Sucrose, glucose, fructose metabolism	915	6138-23-4;	2174,9	361,2
Carbohydrate	Sucrose, glucose, fructose metabolism	100003887		1062	503,2
Lipids	Free fatty acid	100002070	40951-21-1;	1576	247
Lipids	Free fatty acid	100001180	2507-55-3 ;	5345	243,3
Lipids	Free fatty acid	100001579	764-67-0;	5508	271,3
Lipids	Free fatty acid	1239	629-22-1;	5705	299,4
Lipids	Free fatty acid	1231	2091-39-6;	5722	307,3
Lipids	Free fatty acid	100001739	17046-59-2 ;	5600	305,4
Lipids	Free fatty acid	100001335		5955	309,4
Lipids	Free fatty acid	180	60-33-3;	5533	279,3
Lipids	Free fatty acid	100001337		5450	277,3
Lipids	Free fatty acid	818	141-82-2;26522-22-85-0;	606	103,2
Lipids	Oxylipins	100002196		5270	295,2
Lipids	Oxylipins	179	263399-34-4;	5177	313,3
Lipids	Glycerolipids	100001040	2277-28-3;	6226	279,2
Lipids	Glycerolipids	1254	56-81-5;	1311	205
Lipids	Phospholipids	100001395		5574	520,4
Lipids	Phospholipids	100001570		5725	476,3
Lipids	Phospholipids	100001263	17364-16-8;	5671	496,4

Lipids	Phospholipids	100001567		5940	452,3
Lipids	Phospholipids	100001655		5573	571,3
Lipids	Phospholipids	100001271	19420-57-6;	5844	524,4
Lipids	Phospholipids	100001557		5544	520,4
Lipids	Phospholipids	100001776		5650	476,4
Lipids	Phospholipids	100000258	29849-82-9;	1719,7	357,1
Lipids	Phospholipids	1026	1071-23-4;	1737,8	299,1
Lipids	Choline metabolism	267	72556-74-2;	695	184,1
Lipids	Choline metabolism	420	141-43-5;	1304	174,1
Lipids	Sterols	100001033	83-46-5;	2380,8	357,4
Lipids	Sterols	100001269	474-62-4;	2353	343,4
Cofactors, Prosthetic Groups, Electron Carriers	CoA metabolism	1024	137-08-6;	2218	220,1
Cofactors, Prosthetic Groups, Electron Carriers	Nicotinate and nicotinamide metabolism	1312	1094-61-7;	886	335,1
Cofactors, Prosthetic Groups, Electron Carriers	Nicotinate and nicotinamide metabolism	100001310	1341-23-7;	955	255,1
Cofactors, Prosthetic Groups, Electron Carriers	Nicotinate and nicotinamide metabolism	100004035	34441-14-0;	2121,4	186
Cofactors, Prosthetic Groups, Electron Carriers	Nicotinate and nicotinamide metabolism	567	59-67-6;	1334,1	180
Cofactors, Prosthetic Groups, Electron Carriers	Nicotinate and nicotinamide metabolism	100001316	2625-49-2;	1105	256
Cofactors, Prosthetic Groups, Electron Carriers	Oxidative phosphorylation	100001805	7023-27-0;	1221	240,9
Cofactors, Prosthetic Groups, Electron Carriers	Oxidative phosphorylation	461	7664-38-2 ;	1307,7	298,9
Cofactors, Prosthetic Groups, Electron Carriers	Riboflavin and FAD metabolism	500	83-88-5;	3111	377,2
Cofactors, Prosthetic Groups, Electron Carriers	Riboflavin and FAD metabolism	100000251	130-40-5;	2395	455,1
Cofactors, Prosthetic Groups, Electron Carriers	Quinone metabolism	468	84-80-0;	2407	596,5
Cofactors, Prosthetic Groups, Electron Carriers	Ascorbate metabolism	233	134-03-2;	1850,1	332,1
Cofactors, Prosthetic Groups, Electron Carriers	Ascorbate metabolism	301	490-83-5;	1800	245,1
Cofactors, Prosthetic Groups, Electron Carriers	Ascorbate metabolism	100001022	70753-61-6;	1560,7	292,1
Cofactors, Prosthetic Groups, Electron Carriers	Tocopherol metabolism	1105	59-02-9;10191-41-0;	2305,4	502,5
Cofactors, Prosthetic Groups, Electron Carriers	Vitamin B metabolism (B6 or B12)	491	65-22-5;	1210	168,1
Cofactors, Prosthetic Groups, Electron Carriers	Vitamin B metabolism (B6 or B12)	100001121	82-82-6 ;	2210	182,1
Cofactors, Prosthetic Groups, Electron Carriers	Chlorophyll and heme metabolism	100001689	15664-29-6;	6053	593,4
Cofactors, Prosthetic Groups, Electron Carriers	Chlorophyll and heme metabolism	100002535	150-86-7;	1964,7	143,1
Nucleotide	Purine metabolism	211	16373-93-6;	1632	252,1
Nucleotide	Purine metabolism	880	73-24-5;	1003	136,1
Nucleotide	Purine metabolism	798	58-61-7;	1650	268,1
Nucleotide	Purine metabolism	100001694	130-49-4;	1497	346,1
Nucleotide	Purine metabolism	100001449	84-21-9;	1268	346,1
Nucleotide	Purine metabolism	72	37063-35-7;	1484	330,2
Nucleotide	Purine metabolism	1303	19046-78-7;	569	462,1
Nucleotide	Purine metabolism	1002	97-59-6;	1541,3	314,1
Nucleotide	Purine metabolism	172	73-40-5;	1022	152,1
Nucleotide	Purine metabolism	1099	118-00-3;	1675	282,1
Nucleotide	Purine metabolism	74	15718-49-7;	1620	346
Nucleotide	Purine metabolism	361	58-63-9;	1600	267,2
Nucleotide	Purine metabolism	1134	69-93-2;120K5305;	1928	441,2
Nucleotide	Purine metabolism	100000299	146-80-5;	1075	283,1
Nucleotide	Pyrimidine metabolism	827	65-46-3;	1065	244
Nucleotide	Pyrimidine metabolism	73	15718-51-1;	912	306,2
Nucleotide	Pyrimidine metabolism	821	1445-07-4;	1180	245
Nucleotide	Pyrimidine metabolism	825	66-22-8;	1370,4	241
Nucleotide	Pyrimidine metabolism	535	58-96-8;	1430	243,1
Peptide	gamma-glutamyl	331	1116-22-9;	1036	277,1
Peptide	Dipeptide	100003641	686-43-1;	1330	175,1
Peptide	Dipeptide	100001891		2011	203,1
Peptide	Dipeptide	100001890	1638-60-4;	2150	203
Peptide	Dipeptide	100001884	3303-45-5;	1513	189
Peptide	Dipeptide	100003156		2170	246,2
Peptide	Dipeptide	100000803	13433-09-5;	2538	281,1
Peptide	Dipeptide	100001790	19461-38-2;	2080	189,1
Peptide	Dipeptide	100001258	869-19-2;	2236	189,1
Peptide	Dipeptide	100001265	658-79-7;	1728	239,1
Peptide	Dipeptide	100000487	1963-21-9;	1572	175,1

Peptide	Dipeptide	100001707	42537-99-5;	2678	245,1
Peptide	Dipeptide	100003173	22951-98-0;	4059	277,3
Peptide	Dipeptide	100001708	26462-22-6;	2856	245,1
Peptide	Dipeptide	100001709	36077-41-5;	2800	245,1
Peptide	Dipeptide	100003639	20556-16-5;	1073,3	233,1
Peptide	Dipeptide	100003645	3918-92-1;	3560	263,2
Peptide	Dipeptide	100003650	3918-94-3;	2684	215,2
Peptide	Dipeptide	100003166	510-791-9560;	982,3	245,2
Peptide	Dipeptide	100001710	3303-31-9;	3012	245,1
Peptide	Dipeptide	100003182	32949-40-9;	977	245,2
Peptide	Dipeptide	100003590	3918-90-9;	2570	265,1
Peptide	Dipeptide	100003185	686-50-0;	1800	189,2
Peptide	Dipeptide	100003636	27493-61-4;	1278,6	189,1
Peptide	Dipeptide	100003154	1188-24-5;	1620	288,3
Peptide	Dipeptide	100003168	42516-53-0;	1095	259,2
Peptide	Dipeptide	100003169	868-28-0;	1732	189,2
Peptide	Dipeptide	100003174	6403-14-1;	1798	217,2
Peptide	Dipeptide	100003203		1968	231,3
Peptide	Dipeptide	100001125	16875-27-7;	2500	267,2
Peptide	Dipeptide	100003158	3062-14-4;	2310	247,2
Peptide	Dipeptide	100003163	24787-73-3;	1686	203,2
Peptide	Dipeptide	100003179	7298-84-2;	1760	203,2
Peptide	Dipeptide	100003181	14608-81-2;	1418	246,2
Peptide	Dipeptide	100003184		1716	261,2
Peptide	Dipeptide	100003190	6209-12-7;	1519,7	219,2
Peptide	Dipeptide	100001161	3062-07-5;	1346	247,1
Peptide	Dipeptide	100003196	7369-79-1;	1546	260,2
Peptide	Dipeptide	100003201	510-791-9560;	2021	219,2
Peptide	Dipeptide	100003202	6665-16-3;	2106	219,2
Peptide	Dipeptide	100003253		2348	253,2
Peptide	Dipeptide	100003204	50299-12-2;	2204	233,2
Peptide	Dipeptide	100003156		2170	246,2
Peptide	Dipeptide	100003191		1621,9	233,2
Peptide	Dipeptide	100003569	145314-87-0;	1517,3	232,2
Peptide	Dipeptide	100003522	51782-06-0;	1501	205,1
Peptide	Dipeptide	100003534	99032-17-4;	1574,5	219,2
Peptide	Dipeptide	100003640	42854-54-6;	1102	246,2
Peptide	Dipeptide	100003643	22677-62-9;	1601,8	244,2
Peptide	Dipeptide	100003159		2070,6	260,2
Peptide	Dipeptide	100003160		2180	260,2
Peptide	Dipeptide	100003203		1968	231,3
Hormone metabolism	Auxin metabolism	100000916	771-51-7;	3917	157,1
Secondary metabolism	Amine derived	1133	60-19-5;	1503	138,1
Secondary metabolism	Benzenoids	100002610	21056-52-0;	3026	302
Secondary metabolism	Fatty acid and sugar derivatives	100000881	526-99-8;	1899,3	333,1
Secondary metabolism	Flavonoids	100002229	480-18-2;	2210	303,2
Secondary metabolism	Flavonoids	2073	520;	2285,5	559,3
Secondary metabolism	Flavonoids	100002271	480-10-4;	3085	447,2
Secondary metabolism	Flavonoids	100000929	522-12-3;	2987	447,1
Secondary metabolism	Flavonoids	100003115	482-39-3;	3340	431,2
Secondary metabolism	Glucosinolate	100002816	4478-93-7;	3334,3	178,1
Secondary metabolism	Glucosinolate	100003717		2036,7	356,1
Secondary metabolism	Glucosinolate	100002821	505-44-2;	2833,4	164,1
Secondary metabolism	Glucosinolate	100003716	289711-21-3;	2359,9	485,2
Secondary metabolism	Glucosinolate	100003835	21411-41-5;	1273	436
Secondary metabolism	Phenylpropanoids	100001522	458-35-5;	1849	324,1
Secondary metabolism	Phenylpropanoids	100000869	530-59-6;	1996,2	368,2
Secondary metabolism	Phenylpropanoids	100003495	487-36-5;	4167	357,3
Secondary metabolism	Phenylpropanoids	100003494	27003-73-2;	3863	359,1
Xenobiotics	Chemicals	100000856	6850-28-8;	1525	306,2

Cluster and Heat Map

SAMPLE_NAME	MC-00062	MC-00063	MC-00064	MC-00065	MC-00066	MC-00067	MC-00080	MC-00081	MC-00082	MC-00083	MC-00084	MC-00085	MC-00086	MC-00087	MC-00088	MC-00089	MC-00090	MC-00091	MC-00068	MC-00069	MC-00070	MC-00071	MC-00072	MC-00073	MC-00074	MC-00075	MC-00076	MC-00077	MC-00078	MC-00079
CLIENT IDENTIFIER	T0 1	T0 2	T0 3	T0 4	T0 5	T0 6	T06M 1	T06M 2	T06M 3	T06M 4	T06M 5	T06M 6	T24M 1	T24M 2	T24M 3	T24M 4	T24M 5	T24M 6	T06N 1	T06N 2	T06N 3	T06N 4	T06N 5	T06N 6	T24N-1	T24N-2	T24N-3	T24N-4	T24N-5	T24N-6
SAMPLE ID	1351451	1351461	1351459	1351444	1351435	1351443	1351446	1351450	1351466	1351441	1351462	1351467	1351445	1351474	1351449	1351469	1351457	1351436	1351438	1351442	1351454	1351470	1351460	1351437	1351453	1351465	1351452	1351458	1351473	1351468
PARENT SAMPLE ID	1345070	1345071	1345072	1345073	1345074	1345075	1345088	1345089	1345090	1345091	1345092	1345093	1345094	1345095	1345096	1345097	1345098	1345099	1345076	1345077	1345078	1345079	1345080	1345081	1345082	1345083	1345084	1345085	1345086	1345087
FRESH WT MG	100	100	100	100	100	100	100	100	100	100	100	100	100	100	100	100	100	100	100	100	100	100	100	100	100	100	100	100	100	100
TIME HR POST	0	0	0	0	0	0	6	6	6	6	6	6	24	24	24	24	24	24	6	6	6	6	6	6	24	24	24	24	24	24
TREATMENT	PRE	PRE	PRE	PRE	PRE	PRE	MOCK	MOCK	MOCK	MOCK	MOCK	MOCK	MOCK	MOCK	MOCK	MOCK	MOCK	MOCK	300 PPM	300 PPM	300 PPM	300 PPM	300 PPM	300 PPM	300 PPM	300 PPM	300 PPM	300 PPM	300 PPM	300 PPM
TRT GROUP	PRE	PRE	PRE	PRE	PRE	PRE	MOCK 06	MOCK 06	MOCK 06	MOCK 06	MOCK 06	MOCK 06	MOCK 24	MOCK 24	MOCK 24	MOCK 24	MOCK 24	MOCK 24	NOX 06	NOX 06	NOX 06	NOX 06	NOX 06	NOX 06	NOX 24	NOX 24	NOX 24	NOX 24	NOX 24	NOX 24

Cluster Order | chemical_name | IPMC-00062 | IPMC-00063 | IPMC-00064 | IPMC-00065 | IPMC-00066 | IPMC-00067 | IPMC-00080 | IPMC-00081 | IPMC-00082 | IPMC-00083 | IPMC-00084 | IPMC-00085 | IPMC-00086 | IPMC-00087 | IPMC-00088 | IPMC-00089 | IPMC-00090 | IPMC-00091 | IPMC-00068 | IPMC-00069 | IPMC-00070 | IPMC-00071 | IPMC-00072 | IPMC-00073 | IPMC-00074 | IPMC-00075 | IPMC-00076 | IPMC-00077 | IPMC-00078 | IPMC-00079 | LIB ID | COMP ID | PLATFORM | SUPER PATHWAY | SUB PATHWAY | PATHWAY SORT

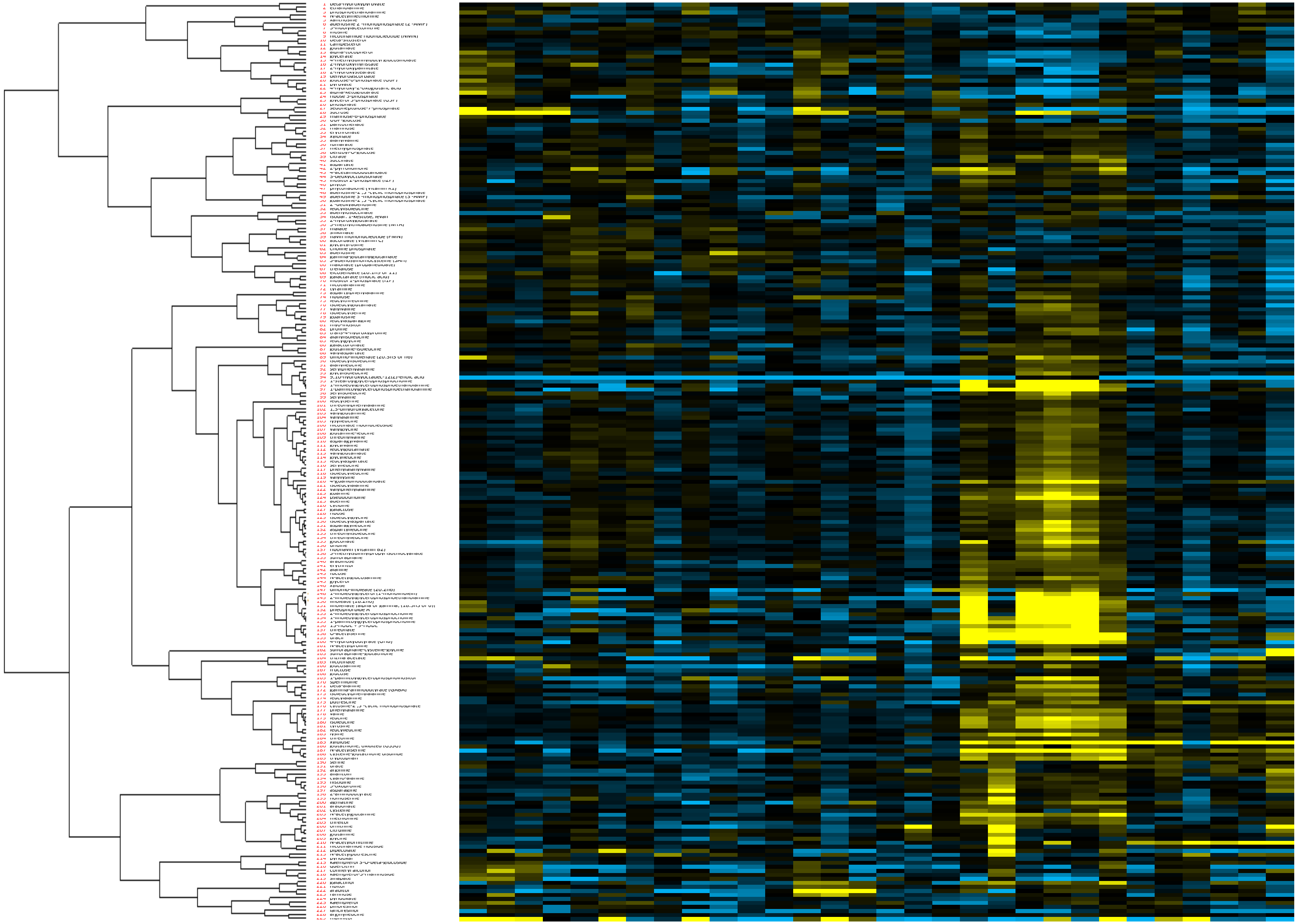

SAMPLE_NAME	IPMC-00062	MC-00063	MC-00064	MC-00065	MC-00066	MC-00067	PMC-00080	MC-00081	MC-00082	MC-00083	MC-00084	MC-00085
CLIENT IDENTIFIER	T0 1	T0 2	T0 3	T0 4	T0 5	T0 6	T06M 1	T06M 2	T06M 3	T06M 4	T06M 5	T06M 6
SAMPLE ID	1351451	1351461	1351459	1351444	1351435	1351443	1351446	1351450	1351466	1351441	1351462	1351467
PARENT SAMPLE ID	1345070	1345071	1345072	1345073	1345074	1345075	1345088	1345089	1345090	1345091	1345092	1345093
FRESH WT MG	100	100	100	100	100	100	100	100	100	100	100	100
TIME HR POST	0	0	0	0	0	0	6	6	6	6	6	6
TREATMENT	PRE	PRE	PRE	PRE	PRE	PRE	MOCK	MOCK	MOCK	MOCK	MOCK	MOCK
TRT GROUP	PRE	PRE	PRE	PRE	PRE	PRE	MOCK 06	MOCK 06	MOCK 06	MOCK 06	MOCK 06	MOCK 06

SAMPLE_NAME	MC-00086	MC-00087	MC-00088	MC-00089	MC-00090	MC-00091	PMC-00068	MC-00069	MC-00070	MC-00071	MC-00072	MC-00073
CLIENT IDENTIFIER	T24M 1	T24M 2	T24M 3	T24M 4	T24M 5	T24M 6	T06N 1	T06N 2	T06N 3	T06N 4	T06N 5	T06N 6
SAMPLE ID	1351445	1351474	1351449	1351469	1351457	1351436	1351438	1351442	1351454	1351470	1351460	1351437
PARENT SAMPLE ID	1345094	1345095	1345096	1345097	1345098	1345099	1345076	1345077	1345078	1345079	1345080	1345081
FRESH WT MG	100	100	100	100	100	100	100	100	100	100	100	100
TIME HR POST	24	24	24	24	24	24	6	6	6	6	6	6
TREATMENT	MOCK	MOCK	MOCK	MOCK	MOCK	MOCK	300 PPM	300 PPM	300 PPM	300 PPM	300 PPM	300 PPM
TRT GROUP	MOCK 24	MOCK 24	MOCK 24	MOCK 24	MOCK 24	MOCK 24	NOX 06	NOX 06	NOX 06	NOX 06	NOX 06	NOX 06

SAMPLE_NAME	MC-00074	MC-00075	MC-00076	MC-00077	MC-00078	MC-00079
CLIENT IDENTIFIER	T24N-1	T24N-2	T24N-3	T24N-4	T24N-5	T24N-6
SAMPLE ID	1351453	1351465	1351452	1351458	1351473	1351468
PARENT SAMPLE ID	1345082	1345083	1345084	1345085	1345086	1345087
FRESH WT MG	100	100	100	100	100	100
TIME HR POST	24	24	24	24	24	24
TREATMENT	300 PPM	300 PPM	300 PPM	300 PPM	300 PPM	300 PPM
TRT GROUP	NOX 24	NOX 24	NOX 24	NOX 24	NOX 24	NOX 24

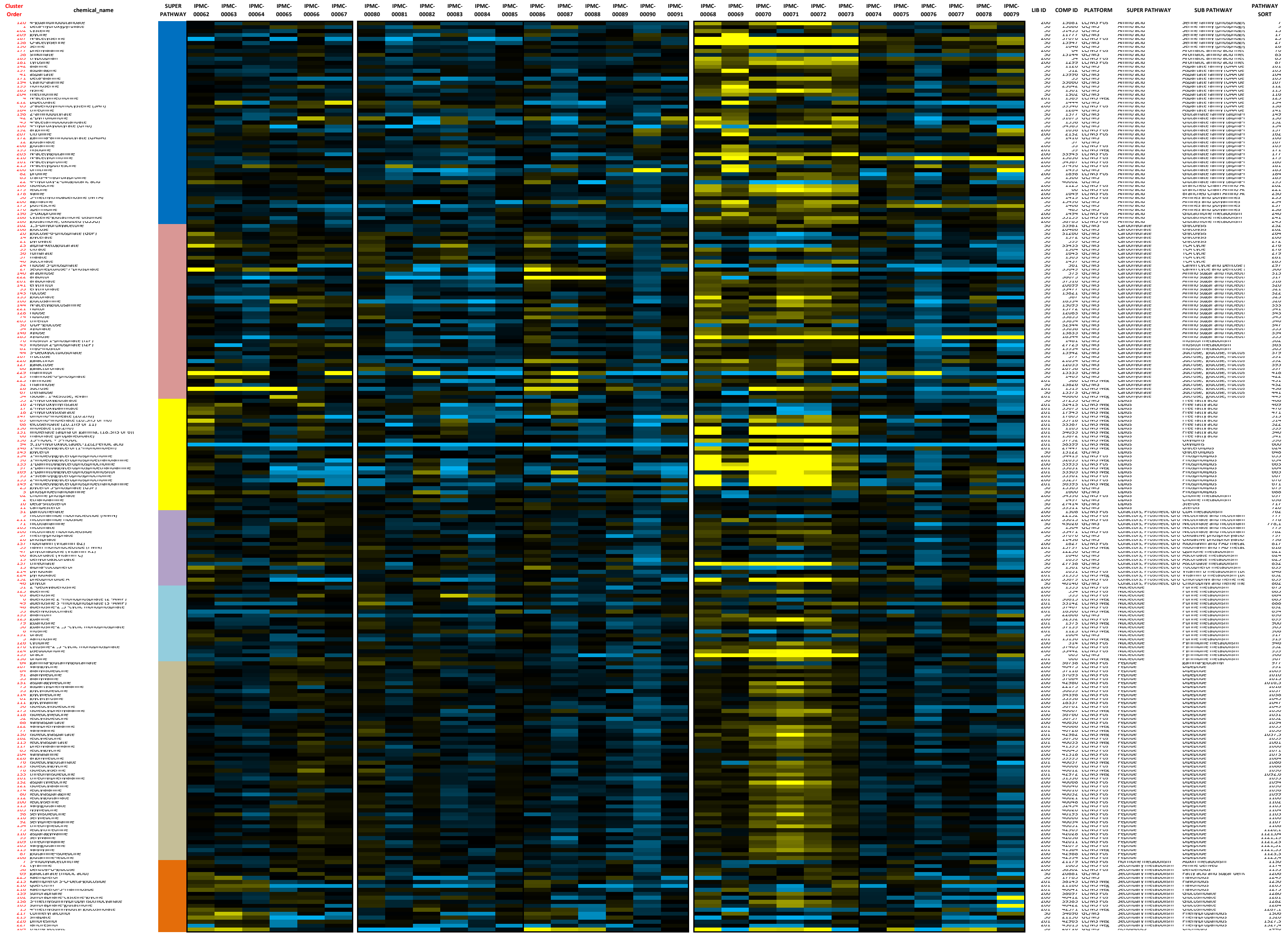

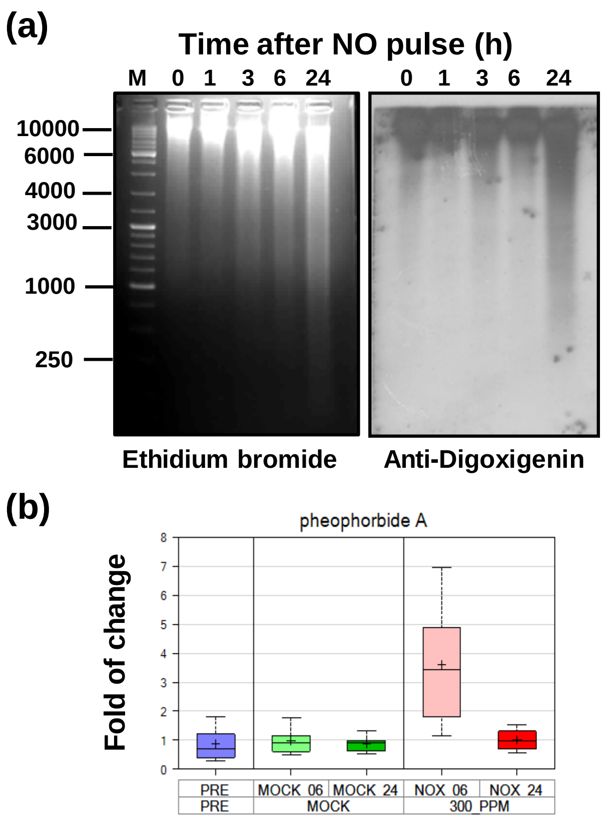

Supplementary Figure S1. NO triggers DNA and chlorophyll degradation. (a) Genomic DNA isolated from NO-exposed plants at the indicated times after application of NO pulse. Genomic DNA was run in duplicate in 2% agarose gels and either ethidium bromide-stained (15 μg) or blotted (5 μg) onto positively charged nylon membranes. DNA digested with *Msp*I was digoxigenin (DIG)-labelled and use as probe for Southern blot with anti-DIG antibody coupled to alkaline phosphatase to amplify the signal. DNA ladder marker (M) in base pairs is shown in the left side. (b) y-axis shows Fold of Change as Group Means Ratios in Scaled Imputed Data for pheophorbide a. + represents mean value; — represents median value; boxes indicate the limits of upper and lower quartile; and error bars represent the maximum and minimum of distribution.
